# Burden of allergic rhinitis in the United Kingdom

**DOI:** 10.3389/falgy.2025.1676574

**Published:** 2025-11-04

**Authors:** Michael Jones, Hilary Shepherd, Diane Hatziioanou, Daphne Martin, Chisomo Mutafya, Ulf Bohman, Susan Hodgson, Rachael Williams

**Affiliations:** 1 Clinical Research Group, Thermo Fisher Scientific, Toronto, ON, Canada; 2 Clinical Practice Research Datalink, London, United Kingdom; 3 Specialty Diagnostics Group, Thermo Fisher Scientific, Uppsala, Sweden

**Keywords:** allergic rhinitis, healthcare resource use, GP visits, hospitalizations, prescriptions, asthma, United Kingdom

## Abstract

**Introduction:**

Allergic rhinitis (AR) is a systemic respiratory condition that is associated with a considerable humanistic burden and is frequently underdiagnosed. Despite the known effects of AR on individual patient well-being, the wider impact of AR on the UK healthcare system remains poorly defined. We aimed to compare healthcare resource use (HCRU) posed by this disease across different age groups between patients who were diagnosed in primary care only vs. those who have a secondary care diagnosis.

**Methods:**

In this retrospective, observational study, patients with an AR record (AR diagnosis) and patients with a record of presenting with AR symptoms but no previous AR diagnosis (AR presentation) in the UK between 2009 and 2019 were defined from primary care and secondary care databases. Patients in the AR diagnosis cohort were further categorized based on whether they had a diagnostic code in primary care only, or any relevant diagnostic code(s) in secondary care for allergist or Ear, Nose, and Throat (ENT) services referrals. Key outcomes included specialist referrals, general practitioner (GP) visits, respiratory-related hospitalizations, GP-prescribed AR-related prescriptions, and coincident asthma.

**Results:**

A total of 3,344,716 patients were defined as presenting signs of AR and 677,771 patients were defined as having an AR diagnosis between 2009 and 2019. Only 11.7% of the AR presentation group received ≥1 referral to an allergist or ENT, and most patients in the AR diagnosis group received a diagnosis in primary care only (89.3%). Compared to their HCRU before diagnosis, patients diagnosed with AR experienced an increase in mean GP visits [7.5–10.0 per patient per year (PPPY)], respiratory-related hospitalizations (5.5–7.1 PPPY), and AR-related medications (mean 8.8–15.0 PPPY). Patients with at least one diagnostic code in secondary care generally reported higher HCRU post-diagnosis than those in primary care. The incidence rate of asthma was lower after AR diagnosis compared to before, with a shorter interval between the onset of asthma and the diagnosis of AR.

**Conclusion:**

Patients with AR impose a greater burden on the UK healthcare system following their diagnosis, especially those who require follow-up from respiratory specialists.

## Introduction

Allergic rhinitis (AR) is a systemic condition that mainly affects the upper airways and is triggered by inhalant allergens such as pollen, mold spores, pet dander, and dust mites (1, 2). The pathogenesis of AR arises from an immunoglobulin E (IgE)-mediated allergic response to mediators such as histamine, leukotrienes, and prostaglandins, and is a member of the atopic triad along with asthma and atopic dermatitis (1, 3). These conditions often co-exist, sharing a common mechanism involving allergic sensitization and a predominance of IgE-mediated immune response, with patients typically presenting with atopic dermatitis first before progressing to AR and asthma (3, 4). Patients with AR have been shown to have decreased general quality-of-life and experience negative impacts on regular activities, health, and well-being (5, 6). Despite this, guidelines from the National Institute of Care Excellence (NICE) recommend that primary caregivers in the United Kingdom (UK) offer symptomatic care as first-line treatment, and refer only patients with more serious symptoms to Ear, Nose, and Throat (ENT) specialists (7). Possibly due in part to the perceived low impact of the condition, many patients with AR opt to self-treat using over-the-counter (OTC) medications, even when diagnosed with other coincident respiratory conditions (8). This reliance on OTC treatment may in turn compel many patients with AR to not seek professional support or receive a formal diagnosis, which could result in underdiagnosis for this population. Underdiagnosis may also be exacerbated by a lack of awareness of the condition by many healthcare professionals, as studies in other European countries have found that only half of the patients who had IgE-confirmed AR were actually identified as having the condition by their healthcare providers (9).

The reliance of secondary care for the management of AR may also put a strain on the UK healthcare system via the need for referrals. The UK National Health Service (NHS) has listed healthcare wait times as a significant concern, and many patients have experienced substantial delays for treatment and referrals (10). While the NHS aims for 65% of patients to start treatment within 18 weeks of referral, this target is frequently missed and waiting lists have grown considerably since the start of the coronavirus disease 2019 (COVID-19) pandemic (10). Referrals to secondary care across the country are also often rejected due to capacity constraints and changes in patient behavior, meaning that many patients with AR may be receiving sub-optimal care due to a lack of clinical resources via the current management pathway (10).

The full scope of this burden is also obscured by a lack of recent epidemiological data on the prevalence of AR in the UK. Physician groups have reported the prevalence of AR to be up to 15% in children and adolescents, and 26% in adults in the UK, but these estimates were based on older literature and no recent studies have been published exploring the prevalence of AR in the UK using real-world primary care data (11–13). Likewise, information exploring the specific effects of AR on the UK healthcare system is relatively sparse and may be affected by various factors. Beyond differences for patients who receive care from ENT specialists, the age at diagnosis may also substantially impact the healthcare resource use (HCRU) of individuals with AR, as many patients develop symptoms during their youth and different management strategies are recommended for children, adolescents, and adults (5, 7). Pregnant women are also managed differently due to the potential for oral drugs to interfere with fetal development, and testing is typically not offered during pregnancy (14, 15).

In this study, we aimed to define patients with AR and probable AR based on symptom presentation in the UK using real-world healthcare data. We compared HCRU [general practitioner (GP) visits, hospital visits, and medicines prescribed] in these two groups and stratified results by patients who were diagnosed in primary care only vs. those who have a secondary care diagnosis. We also examined rates of referrals to ENT specialists by age group and incidence of asthma by year to provide a greater understanding of the healthcare burden on systems and patients of diagnosed and potentially under-diagnosed AR.

## Methods

In this retrospective, observational study, patients with an AR record (AR diagnosis), and patients with a record of presenting with AR symptoms but no previous AR diagnosis (AR presentation) were drawn as two cohorts. GP data from the Clinical Practice Research Datalink (CPRD) Aurum database, a UK primary care data source, was used to define the AR presentation cohort, with the AR diagnosis cohort additionally linked to secondary care Hospital Episode Statistics (HES) Admitted Patient Care (APC) and Outpatient (OP) databases (16–18). The source population comprised all research acceptable patients with at least one day of registration between January 1st, 2009, and December 31st, 2019, and who were eligible for linkage to patient level Index of Multiple Deprivation (IMD), HES APC, and HES OP (17–19).

**Figure 1:**
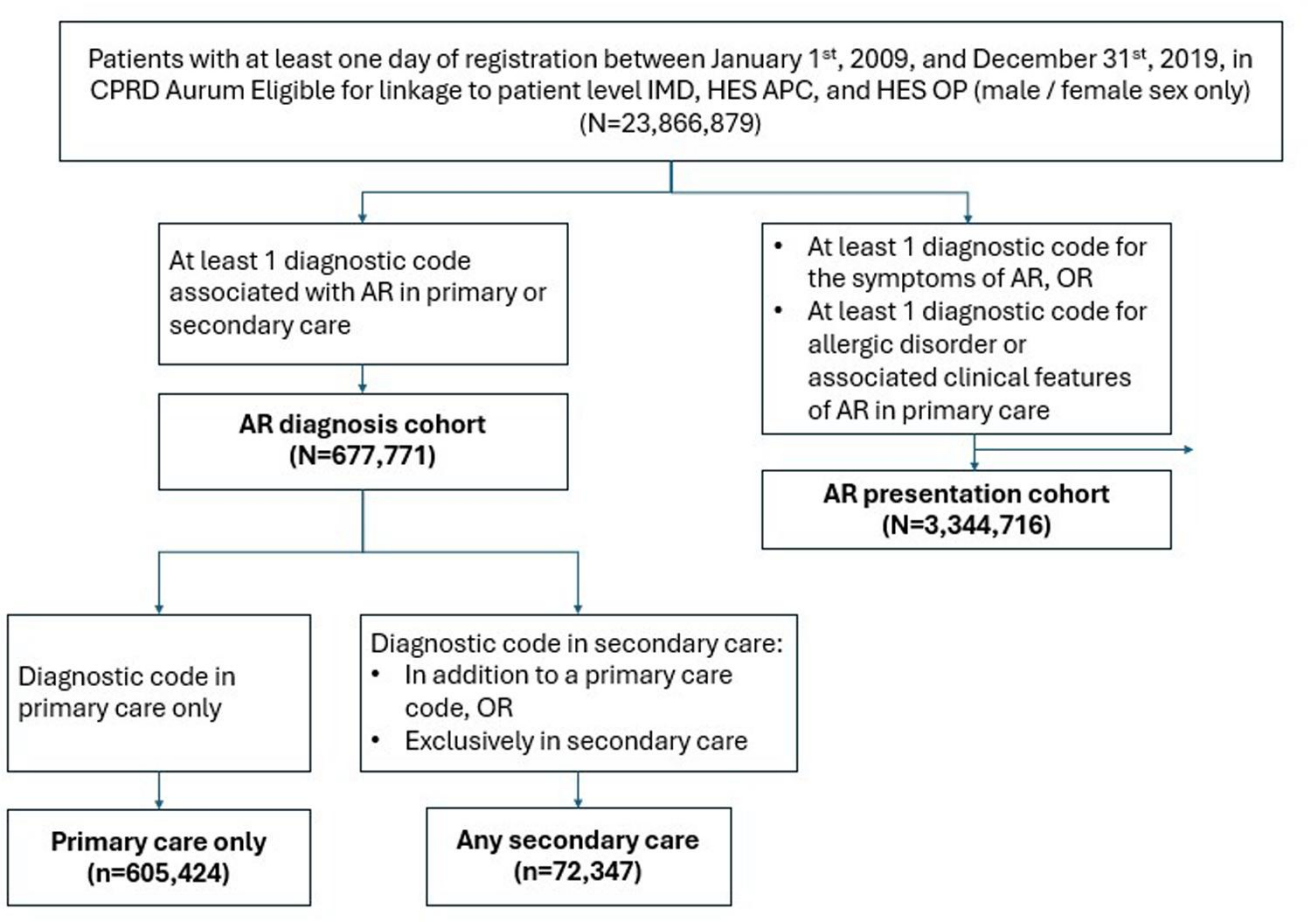
Cohort flow diagram.

The inclusion criteria for the AR diagnosis cohort were at least one diagnostic code associated with AR in primary or secondary care. The AR presentation cohort was defined in primary care only in patients who had rhinitis symptoms but no previous record of an AR diagnosis event. To be included, patients needed at least one diagnostic code for the symptoms of AR, one or more diagnostic codes for allergic disorder, or one or more diagnostic codes for the associated clinical features of AR in primary care (Figure 1; see Supplementary Tables S1A,B, S2A,B, S3 for a full list of codes). For both cohorts, where there were multiple event dates, the earliest was considered the index date. Patients were excluded from the AR presentation cohort if they ever had a record of AR prior to their presentation index date. This included looking at time prior to study start and prior to patient registration at the GP practice to confidently exclude prevalent cases. If patients from the AR presentation cohort met the AR case definition following an AR presentation, they remained in the presentation cohort until the day before they met the AR definition and then joined the AR cohort. All codelists were developed by searching for relevant terms and Systematized Nomenclature of Medicine—Clinical Terms (SNOMED-CT) categories in the CPRD Code Browser. Feasibility counts showed that approximately 2.6 million patients had AR presentation and were eligible for linkage to HES APC or HES OP data, which was considered sufficient to enable a robust assessment of the stated objectives. All eligible participants in the database during the study period were included, as respiratory allergies are generally lifelong (20). Patients in the AR diagnosis cohort were further categorized based on whether they had a diagnostic code in primary care only (the “primary care only” group), or any relevant diagnostic code(s) in secondary care either in addition to a primary care code or exclusively in secondary care (the “any secondary care” group).

The date range for outcome analysis was terminated in December 2019 to avoid the confounding effects of the COVID-19 pandemic. The start of follow-up was defined as the latest of patient registration at the GP and study start (January 1st, 2009). Patients were followed up until the earliest of end of patient registration, date that data was last received from the GP practice (last data collection date from the GP practice), study end date, and the CPRD derived death date (21). Patients were excluded if their start of follow-up was after their end of follow-up date. The index date was the date of the first ever recorded code; for patients who had different dates for their rhinitis symptoms or allergic disorder/clinical features of AR in the AR presentation cohort, the index date was taken to be the earliest date. Medical codes for allergy disorders included general allergy codes, pollen, mold, food allergies, animal allergies, IgE-mediated allergies, non-IgE-mediated allergies, non-drug allergy, seasonal allergy, and family history of allergy (See Supplementary Table S2A for a complete list). Medical events with no associated date were excluded, as the timing of these events could not be established. Only events occurring within each patient's follow-up period were considered.

Baseline demographic characteristics, stratified by AR diagnosis or AR presentation status, included age, sex, and patient level deprivation. For all patients, date of birth was calculated using the year of birth and, where available (for those aged under 16 years), the month of birth. July was assumed if the month was missing, and the 1st day of the month was imputed for all patients. Quintiles of patient level IMD data were also used to provide a proxy deprivation score for every patient. HCRU outcomes included the number of allergy specialist referrals for the AR presentation group, as well as GP visits, respiratory-related hospital visits, and AR-related prescriptions for the AR diagnosis group. GP visits were counted as the number of primary care consultations per patient annually. These visits included telephone and face-to-face consultations, nursing home, residential home, and home visits, emergency, urgent, and routine appointments, online communications (such as telemedicine via web camera and e-mail), and consultations via Short Message Service (SMS) text messages (22). For referral rates to allergy specialists and primary care consultations, counts excluded all events occurring before the AR presentation/diagnosis date or after a patient's end of follow-up (see Supplementary Table S4 for a complete list of codes used to define referrals). Referrals were defined either by searching for a medical code relating to referrals in the GP record or by counting inpatient hospitalizations and outpatient visits with a specialty label of “Ear Nose and Throat Service” or “Allergy service”. For GP outcomes, patients whose primary care events were not associated with a consultation record within their follow-up period were excluded. Respiratory-related inpatient hospital visits were also analyzed for the AR diagnosis cohort, and every relevant event within a patient's follow-up time from HES APC or HES OP was counted. Hospitalizations relating to respiratory conditions were defined using the International Classification of Disease-10th revision (ICD-10) codes (the full list can be found in Supplementary Table S5). AR-related prescriptions (including oral antihistamines, intranasal corticosteroids, and nasal anticholinergics; see Supplementary Table S6 for a complete list) were examined for both the AR diagnosis and AR presentation cohorts, where any prescription that occurred on the same day as AR diagnosis or outside follow-up was excluded. The incidence of asthma was also measured for the AR diagnosis group and considered the first ever occurrence of asthma in a patient's medical record before or after AR diagnosis (the list of asthma event codes can be found in Supplementary Table S7). Only one asthma occurrence was counted per patient. The mean interval (in days) between asthma diagnosis and AR diagnosis was also calculated. While AR events must have occurred within a patient's follow-up time, asthma events that occurred before the start of follow-up were also included to account for events that occurred at a previous GP practice.

HCRU outcomes in the AR diagnosis cohort were also explored separately for patients who had a diagnosis of AR in primary care only vs. those who had a record of any diagnosis in secondary care. Outcomes were also investigated by age at index date (either AR presentation or AR diagnosis), comparing pediatric (0–12 years), adolescent (13–18 years, from the generally accepted age of puberty onset to the legal age definition of an adult), and adult patients (>18 years) (23).

Data was analyzed with the latest installed version of Stata SE (17.0) and Microsoft Excel. HCRU outcomes were calculated as mean events per patient per year (PPPY), or incidence rates [number of patients or events per specified patient-year (PY)]. Simple descriptive statistics were used to summarize the data, and confidence intervals were not calculated.

## Results

We defined 677,771 patients as having an AR diagnosis between 2009 and 2019, of whom 605,424 (89.3%) had an AR record in primary care only, and 72,347 (10.7%) had an AR record in secondary care (Table 1). Additionally, 3,344,716 patients with AR presentation were defined over the same period, and 138,556 were included in both the AR presentation and AR diagnosis cohorts. Baseline demographic characteristics were broadly similar between all study groups and were largely consistent with the general UK population (24). A slightly higher proportion of patients were from the most deprived quintile than the least deprived, compared to the distribution across the UK (25). Most included patients in each cohort were adults >18 years old, with only 7% of the AR diagnosis cohort and 4.5% of the AR presentation cohort being adolescents aged 13–18 years old at start of follow-up.

**Table 1:** Baseline demographic characteristics of the study populations.

Characteristic	AR presentation	AR diagnosis	AR diagnosis by care group	
			AR record in primary care only	AR record in secondary care
N	3,344,716	677,771	605,424	72,347
Mean age at start of follow-up (SD)	31.0 (25.2)	28.9 (21.8)	28.7 (21.8)	30.8 (21.8)
Age at start of follow-up				
0–12 years	31.3%	30.2%	30.7%	26.6%
13–18 years	4.5%	7.0%	7.1%	5.8%
>18 years	64.2%	62.8%	62.2%	67.7%
Sex				
Male	46.0%	47.9%	48.1%	46.8%
Female	54.0%	52.1%	51.9%	53.2%
Patient deprivation level				
1 (least deprived)	19.1%	17.6%	17.6%	18.0%
2	19.5%	18.0%	17.9%	19.0%
3	19.3%	18.7%	18.7%	18.7%
4	20.8%	22.6%	22.7%	21.5%
5 (most deprived)	21.4%	23.1%	23.1%	22.9%

### Referrals to allergy specialists

Referrals to allergy specialists following AR presentation were infrequent, with only 11.7% of patients receiving ≥1 referrals between 2009 and 2019, representing a rate of 11,282 patients per 100,000 PYs of follow-up (total follow-up duration: 9,741,662 years). Referral rates were largely similar across age groups and remained under 15%: 9.5% of children aged 0–12 years (referral rate: 12,665 per 100,000 PYs), 7.7% of adolescents aged 13–18 years (10,856 per 100,000 PYs), and 13.0% of adults aged >18 years (10,887 per 100,000 PYs).

### GP visits and respiratory-related hospital visits

**Figure 2:**
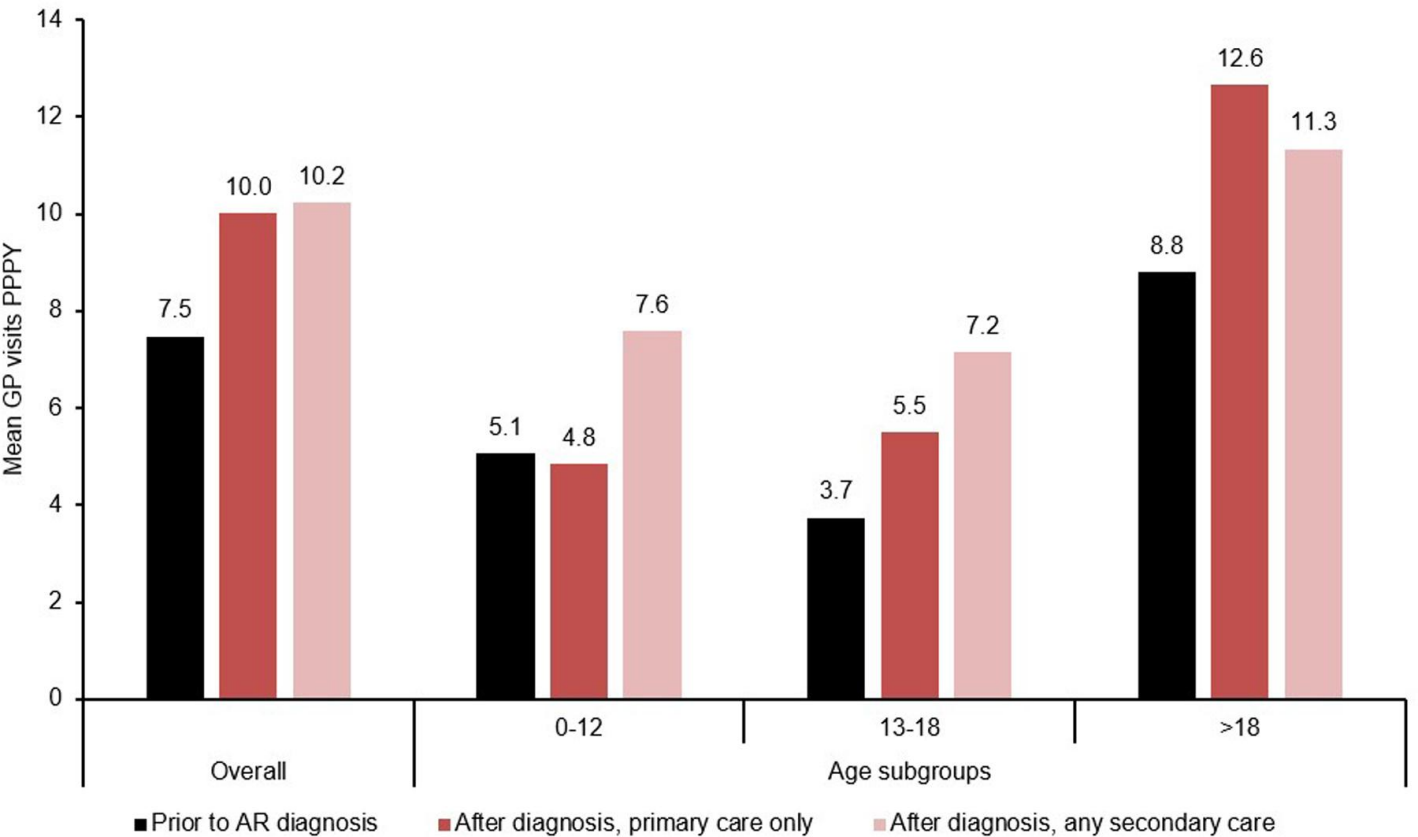
GP visits by AR diagnosis status.

Patients with an AR diagnosis (from any source) visited their GPs more frequently after receiving their diagnosis compared with before, increasing from a mean of 7.5 visits PPPY pre-diagnosis to a mean of 10.0 visits PPPY post-diagnosis, representing a 34% increase (Table 2). The rate of GP visits were similar for patients with a primary care diagnosis only vs. those with an AR record in secondary care (10.0 PPPY and 10.2 PPPY, respectively; Table 2 and Figure 2). However, the increase from pre-diagnosis levels seems to have been driven by adolescent and adult patients; children ≤12 years old who received a primary care diagnosis only reported similar mean GP attendance rates compared to the full pre-diagnosis group (5.1 PPPY prior to diagnosis vs. 4.8 PPPY post-diagnosis, respectively, Table 2 and Figure 2).

**Table 2:** Healthcare visits in patients with an AR diagnosis, by care group.

Age group at index	Timeframe	GP visits		Respiratory-related hospital visits			
		Patients with ≥1 visit	Mean visits PPPY	Patients with ≥1 hospitalization	Total follow-up time (years)	Mean visits PPPY	Rate of hospitalizations per PY
Overall	Prior to diagnosis	640,338	7.5	131,869	1,158,440	5.5	0.62
	After diagnosis	657,912	10.0	173,658	1,504,857	7.1	0.82
	After diagnosis (primary only)	587,390	10.0	124,003	1,082,524	6.3	0.72
	After diagnosis (any secondary)	70,522	10.2	49,665	422,333	9.3	1.09
0–12 years	Prior to diagnosis	163,433	5.1	29,693	246,138	3.7	0.45
	After diagnosis	164,886	5.1	32,635	276,848	4.5	0.53
	After diagnosis (primary only)	149,562	4.8	21,672	185,768	3.7	0.43
	After diagnosis (any secondary)	15,324	7.6	10,963	91,080	6.1	0.73
13–18 years	Prior to diagnosis	56,835	3.7	6,849	65,209	4.2	0.44
	After diagnosis	58,595	5.6	10,315	95,201	5.1	0.55
	After diagnosis (primary only)	53,957	5.5	7,344	68,061	4.2	0.45
	After diagnosis (any secondary)	4,638	7.2	2,971	27,140	7.2	0.79
>18 years	Prior to diagnosis	420,070	8.8	95,327	847,093	6.1	0.68
	After diagnosis	434,431	12.5	130,708	1,132,808	8.0	0.92
	After diagnosis (primary only)	383,871	12.7	94,987	828,695	7.0	0.81
	After diagnosis (any secondary)	50,560	11.3	35,721	304,113	10.4	1.22

Hospitalization incidence rate represents the total number of hospital visits/follow-up years among patients with ≥1 event hospital visit.

An overall increase in respiratory-related hospital visits was also observed following the diagnosis of AR, with the mean number of visits PPPY increasing from 5.5 pre-diagnosis to 7.1 post-diagnosis for the whole AR diagnosis cohort: this increase was driven primarily by patients with any record in secondary care (9.3 PPPY, 60% increase from full cohort pre-baseline values) with a similar trend observed across each age group (Table 2). The proportion of patients reporting ≥1 hospital visit also increased from 19% pre-diagnosis to 26% post-diagnosis, with a higher proportion of patients in the any secondary care group reporting ≥1 hospital visit post-diagnosis compared to the primary care only group (69% vs. 20% respectively). A similar trend was observed across all age groups (Table 2).

### AR-related prescriptions

**Figure 3:**
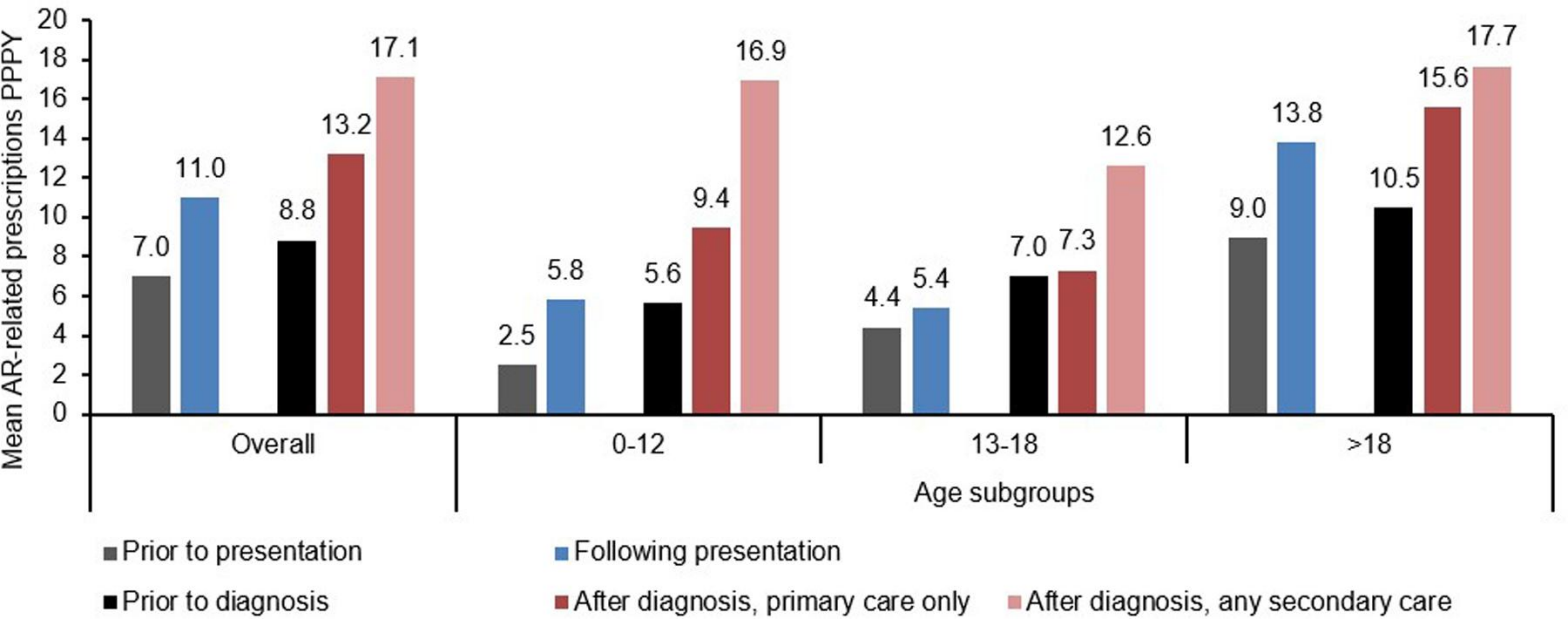
Average number of AR-related prescriptions prior and following presentation and diagnosis.

Overall, prescriptions PPPY increased after diagnosis of AR relative to pre-diagnosis levels (Table 3). This pattern was stronger for adults or children compared to adolescents, for whom the mean prescriptions PPPY only increased by 9% post-diagnosis (from 7.0–9.6 PPPY; Table 3). AR patients who received any secondary care diagnosis were given more prescriptions from GPs over the course of their follow-up compared to patients who received a primary care diagnosis only, with a 98% increase from the full group levels in prescription rate after any secondary care diagnosis vs. a 53% increase after primary care diagnosis only. The proportion of patients with ≥1 AR-related prescription from their GP also increased from 45% before diagnosis to 63% after diagnosis (Table 3), and an overall 57% increase in mean prescriptions PPPY was observed comparing individuals before the presentation of AR-related symptoms/conditions vs. afterwards, with increases ranging from a 23% increase for adolescent patients to a 132% increase for pediatric patients (Figure 3). Comparing patients in the primary care only group vs. the any secondary care group, the proportion of patients receiving ≥1 AR-related prescription after diagnosis was comparable (63% and 59%, respectively; Table 3). However, the total rate of prescriptions (total prescriptions among patients with ≥1 AR-related prescription per 100,000 PYs of their respective follow-up) was 30% higher for the any secondary care group vs. the primary care only group, with this increase varying substantially across age groups from 11% for adults to 90% for pediatric patients (Table 3).

**Table 3:** AR-related prescriptions in patients with an AR diagnosis and AR presentation, by care group.

Age group at index	Timeframe	Patients with ≥1 prescription	Total follow-up time (years)	Mean prescriptions PPPY	Rate of prescribing per PY
Overall	Prior to presentation	749,792	5,957,667	7.0	0.88
	Following presentation	1,313,493	10,352,535	11.0	1.39
	Prior to diagnosis	307,735	2,659,815	8.8	1.02
	After diagnosis	425,661	3,595,061	15.0	1.61
	After diagnosis (primary only)	383,337	3,238,050	13.2	1.56
	After diagnosis (any secondary)	42,324	357,011	17.1	2.03
0–12 years	Prior to presentation	207,792	1,394,904	2.5	0.38
	Following presentation	410,844	2,853,723	5.8	0.83
	Prior to diagnosis	85,675	722,169	5.6	0.67
	After diagnosis	114,761	984,885	12.6	1.19
	After diagnosis (primary only)	102,381	884,019	9.4	1.09
	After diagnosis (any secondary)	12,380	100,867	16.9	2.08
13–18 years	Prior to presentation	29,299	258,325	4.4	0.50
	Following presentation	45,709	380,945	5.4	0.65
	Prior to diagnosis	27,033	249,403	7.0	0.76
	After diagnosis	37,429	330,457	9.6	0.87
	After diagnosis (primary only)	34,501	303,905	7.3	0.83
	After diagnosis (any secondary)	2,928	26,552	12.6	1.39
>18 years	Prior to presentation	512,701	4,304,438	9.0	1.07
	Following presentation	856,940	7,117,867	13.8	1.66
	Prior to diagnosis	195,027	1,688,244	10.5	1.21
	After diagnosis	273,471	2,279,719	16.6	1.89
	After diagnosis (primary only)	246,455	2,050,126	15.6	1.87
	After diagnosis (any secondary)	27,016	229,593	17.7	2.08

Rate of prescribing represents the total number of AR-related prescriptions/follow-up years among patients with ≥1 AR-related prescription.

In the AR presentation group, an increase in AR-related prescriptions was also observed following the onset of AR presentation, with the trend conserved across all age groups (Table 3).

### Onset of coincident asthma

**Figure 4:**
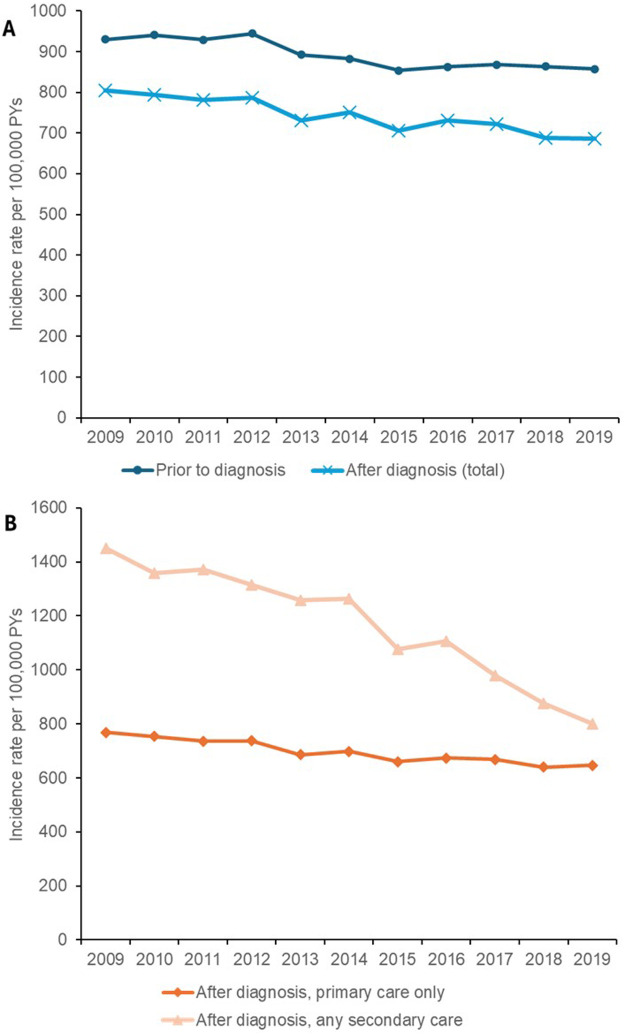
Coincident asthma for patients diagnosed with AR.

**Figure 5:**
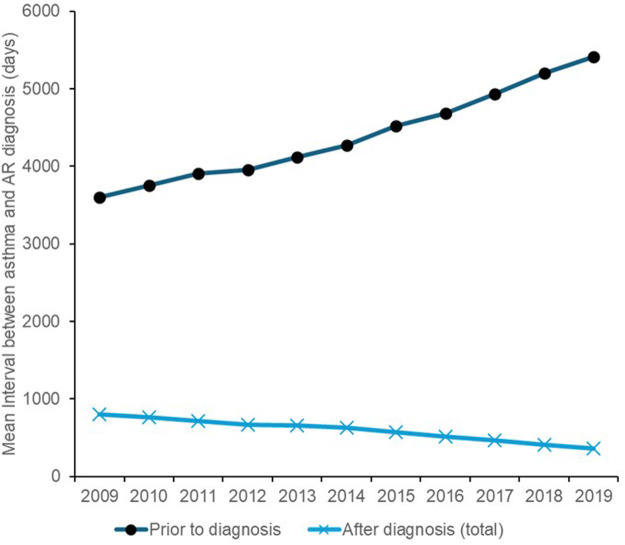
Time interval between asthma and AR diagnosis.

Across the full study follow-up period, the incidence rate of asthma was lower after AR diagnosis compared to before, dropping from a mean rate of 895 new patients per 100,000 PYs pre-diagnosis to 747 new patients per 100,000 PYs post-diagnosis. The annual incidence rate of asthma in patients with AR remained stable from 2009–2019 for patients prior to diagnosis (Figure 4A) or among those who only received a diagnosis in primary care, but this rate considerably declined over time in patients who received any secondary care diagnosis of AR, dropping from 1,450 new cases per 100,000 PYs in 2009–800 new cases per 100,000 PYs in 2019 (Figure 4B). The mean interval between the index dates of asthma and AR was also substantially shorter for patients who already had an AR diagnosis (559 days or 1.5 years) compared to those who were not yet diagnosed (4,333 days or 11.9 years), with the mean duration increasing over time from 2009–2019 for the pre-AR diagnosis group compared to the post-AR diagnosis group (Figure 5) which decreased over time. Post-AR diagnosis, the interval between the onset of asthma and AR diagnosis was also noted to be an average of 162 days shorter for patients in the any secondary care group (mean interval of 478 days) compared to those in the primary care only group (mean interval of 640 days).

## Discussion

### Impact of AR on HCRU

In this real-world evidence study of patients with AR in the UK, 677,771 patients were defined with AR between 2009 and 2019, the majority of whom (89.3%) were recorded in primary care only. Overall, people with AR visited GPs and hospitals more frequently, and received more frequent prescriptions for AR-related medications, after their AR diagnosis compared to before. HCRU was also consistently higher for patients who had any diagnosis code in a secondary care source (HES APC or HES OP) compared to those who only had a diagnosis of AR in primary care. The overall increase in HCRU post-diagnosis vs. pre-diagnosis levels suggests that patients with AR can incur a quantifiable clinical burden to the UK healthcare system, and the increased HCRU for patients in the secondary care group aligns with current NICE guidance for referring patients with more severe AR to an ENT specialist (7). The underlying reasons for the greater HCRU observed in patients with secondary care diagnoses could not be determined due to the preliminary and exploratory nature of this study. This question warrants further investigation in future studies to better understand the drivers of increased HCRU among patients with AR. The frequency of GP attendance was higher for all patients in this analysis (ranging from 7.5 PPPY to 10.2 PPPY, depending on diagnosis status and location of diagnostic code) compared to a previous study of general GP attendance in the UK, which reported 5.16 visits PPPY in 2013 (26). While this increase may reflect the increased burden of disease for patients with AR, it should be noted that this earlier study used the CPRD General Practice Online Database (GOLD) rather than the CPRD Aurum data, and the different GP software systems as well as the coding systems, geographical coverage, population size, and data recency that underlie these different databases complicates any direct comparisons (26–28). The increase in GP attendance after diagnosis was primarily driven by adolescents and adults, while children aged 0–12 years had approximately similar GP attendance rates before and after diagnosis when the diagnosis was recorded in primary care only. This may be partly because children already have a high baseline for regular GP attendance and routinely visit their GP for reasons unrelated to a specific condition such as vaccinations (29).

For both hospitalizations and AR-related prescriptions, although the mean number of events per patient increased following diagnosis of AR, the rates of individuals with ≥1 hospitalization or ≥1 prescription only marginally increased post-diagnosis. These data suggest that a subset of patients with the most severe disease are driving the increase in the total number of events. These rates were broadly similar between the primary care and secondary care diagnosis groups, and between age groups, suggesting that this high-burden subgroup is not characterized by these variables alone. Furthermore, analysis of the prescription data may be confounded by a general decline in overall prescribing from 2009–2019 (data not shown), which may reflect either a data bias or an increase in OTC medications for respiratory symptom management (30). Such OTC use could result from underdiagnosis of AR (i.e., if patients never receive a formal diagnosis and therefore manage symptoms themselves), or from patient preference for faster relief (e.g., choosing OTC topical nasal decongestants over intranasal corticosteroids, which have a slower onset of action) (31, 32). Additionally, prescription data from GPs might be underreported in the CPRD Aurum database; in a previous study of a specialty treatment administered for symptoms associated with a chronic inflammatory disease, inconsistencies were observed between the number of prescriptions captured by CPRD Aurum and the actual number received by users (33). Moreover, medicines routinely prescribed in hospitals are not captured in the available HES APC dataset, which may affect data recording for the subset of patients with more serious conditions. However, if these trends are driven by a high severity patient group, further research will be required to elucidate the specific disease characteristics of this population (e.g., comorbidities and sensitization to specific allergens). It should also be noted that prescriptions may have been misclassified in this study, as some recorded as AR-related could have been issued for other conditions with similar symptoms, such as non-allergic rhinitis, which are often misdiagnosed in primary care (34) as the indication for prescription is not included in the data.

Finally, case definitions of AR and AR presentation were tested with both a broad and restrictive set of inclusion criteria. The restrictive definitions required patients to have at least one record of a relevant prescription (e.g., leukotriene receptor antagonists, nasal anticholinergics, nasal corticosteroids) in the AR diagnosis cohort and at least one treatment code for symptoms of AR (e.g., nasal decongestants, antihistamines) in the AR presentation cohort. However, since the indications for prescriptions could not be determined, restrictive definitions resulted in lower sample sizes than expected (652,369 for AR presentation and 333,491 for AR diagnosis), the broader case definition for both cohorts was favored to better capture the expected undiagnosed AR cohort.

During our study period of 2009–2019, over 3.3 million patients were defined that presented with signs and symptoms of AR but did not have a diagnosis, representing a population 4.9-fold larger than patients who received a diagnosis of AR over the same period. In conjunction with the low referral rates to allergy specialists following AR presentation, these data suggest that AR may be substantially underdiagnosed in the UK. A survey of GPs in the UK in 2005 found that fewer than 25% of GPs met the full consensus standards for the diagnosis and management of AR, and our results suggest that further improvements to the identification and management of AR may still be necessary for primary care providers in the UK (35). However, long waiting times for appropriate AR diagnosis may also contribute to the low percentage of AR diagnoses, with the NHS reporting an average wait of 13–18 weeks after referral to an allergy clinic (36). Misinformation about AR is another concern, as patients may gather information from unvetted online sources such as social media and video platforms. For example, one case involved widespread advertising by a UK clinic promoting unapproved hay fever injections for the treatment for AR (37). This prompted the Medicines and Healthcare Products Regulatory Agency (MHRA) and the Committee of Advertising Practice (CAP) to warn UK organizations to stop advertising the product and remove all related content from social media (37). Furthermore, one study found that 36% of YouTube videos on AR contained misleading information (38).

AR and asthma often co-exist due to shared pathophysiological mechanisms as part of the atopic triad, with earlier studies estimating a 40% prevalence of asthma among patients with AR in the UK (3, 39). In this study, the overall incidence of asthma was lower after AR diagnosis compared to before. AR treatment has been shown to reduce the development of asthma, with one Italian randomized open trial reporting a reduced risk of asthma after 3 years of treatment with coseasonal sublingual immunotherapy in children with AR, compared to control subjects (40). These findings underscore the common mechanisms of AR and asthma and support the idea that treating one condition may help prevent the other (40). Immunotherapy for either condition may also modify the patient's immune response to the other (40). However, the lower post-diagnosis incidence of asthma may also reflect that many patients had already been diagnosed with asthma prior to their AR diagnosis, leaving fewer patients at risk of developing asthma afterwards and hence being counted as new cases. This study did not report the mean age of AR diagnosis, hence, given the long waiting times for AR diagnosis in the UK, it is possible that most asthma cases were already diagnosed by then, contributing to the perceived lower incidence after AR diagnosis compared with before (36). In addition, asthma incidence has been decreasing over time in the UK, with one analysis across England, Wales, South-East Scotland, and Northern England showing lower rates in 2019 than in 2005 (41). Among all age groups, children aged <10 years had the most pronounced decline (41). This may reflect that asthma symptoms in young children are increasingly diagnosed as “pre-school wheeze”, leading to under-reporting, and that clinicians are keen to avoid over-diagnosis (41). We also observed, in this *post-hoc* analysis, lower asthma incidence in the primary care group than in the secondary care group, which may suggest that earlier diagnosis and management of AR in primary care could reduce the likelihood of developing asthma, while undiagnosed AR, which may be more likely to be identified in hospital settings, is associated with a higher risk of asthma. However, as asthma was defined using primary care medical codes only, it is possible that using HES APC data would have resulted in the definition of more asthma cases (most likely, the more severe cases). Interestingly, a substantial decrease in the annual incidence of asthma was observed for patients who had any secondary care diagnosis code. This trend may be partially driven by our methodology, where asthma events that occurred before a patient's follow-up start date were included in the analysis to capture relevant events recorded at previous GP practices, which may have led to inflated asthma event counts in earlier years. However, allergy specialists and ENTs generally have more sophisticated means for the diagnosis and treatment of allergic disorders, and this trend may reflect gradual changes in these practices (e.g., an increasing adoption of blood-based IgE allergen testing) that help detect patients with allergies more reliably and prevent their asthma from triggering in the first place (42). Exploring the specific drivers of this decrease will require further research.

An exploratory analysis of the CPRD Aurum database (primary care only, as coverage of the linkages to HES APC or HES OP did not extend to 2023 at the time of analysis) found 27,202 patients matching the AR diagnosis cohort criteria and 149,504 patients matching the AR presentation cohort criteria in 2023, suggesting that this discrepancy may remain an ongoing problem after the COVID-19 pandemic. Interestingly, the distribution of patient-level deprivation in both cohorts was slightly more even in 2023 and more in line with the expected distribution in the UK, which may suggest that AR has been diagnosed in a more equitable manner since the end of the pandemic (25). The date range for HCRU outcomes analyses was terminated in December 2019 to avoid the confounding effects of the COVID-19 pandemic.

This study had several limitations. As with all research using electronic health records, the quality of the data can vary between contributing healthcare providers and over time, as data are collected for the purposes of patient care, not for secondary use in research. In addition, CPRD Aurum represents data from England only. Therefore, generalizability of the findings to other countries, including other developed nations, may be limited, particularly where the patient population and healthcare practices differ. Data on HCRU in AR are sparse in both the UK and internationally, making it difficult to generalize the study results. Furthermore, a clear definition of patients with AR and those who present with AR was difficult to achieve as symptoms and diagnoses can be extremely heterogenous. Nonspecific medical codes, which can refer to many symptoms or conditions, were common (especially for AR symptoms) and may compromise the specificity and sensitivity of the patient cohorts. Uncertainty regarding the indication for prescriptions led to the decision not to use prescriptions as part of the cohort inclusion criteria in this *post-hoc* analysis. In future studies, researchers could limit prescriptions to those within a defined time window around a diagnosis code to minimize this risk, but this approach may be too restrictive, especially for chronic conditions, where drugs are often prescribed years after the initial diagnosis. Another limitation for this study is that patients with milder forms of AR may seek medical advice and treatment at local pharmacies, which is not captured in the CPRD data. Additionally, as the observation window for events was curtailed to 2019, patients diagnosed with AR in 2019 may have subsequently developed asthma after the end of the observation period, especially given the often-lengthy interval between asthma and AR development. This, in addition to the possibility that some patients may already have had prevalent asthma and therefore could not be counted as incident cases, could explain the lower recording of incident asthma in later years. Finally, the quality of referral recording was unknown, and referrals made outside the NHS (i.e., through private healthcare) were not captured.

Potential sources of bias should also be considered. Misclassification bias was possible due to the variability in the specificity of the codes used to define AR diagnosis. While some codes were highly specific and more likely to capture true occurrences of AR (e.g., SNOMED-CT ConceptID 61582004 for “Allergic Rhinitis”), others were less specific (e.g., SNOMED-CR ConceptID 367498001 “Hay Fever”). An inclusion criterion for an AR-specific prescription was originally tested for both cohorts, but this was ultimately removed from the study due to the substantial annual decrease observed for GP prescriptions over time. This change may have mitigated misclassification bias to some extent, as prescriptions are not linked to specific indications in CPRD, but the broadened population may also have increased the likelihood of including people without a true AR diagnosis. It is also possible that some patients with AR did not have a formal diagnosis code recorded, even if they were receiving treatment, and were therefore mistakenly classified into the AR presentation cohort only. Surveillance bias is another potential limitation: patients with more frequent GP visits may be more likely to be diagnosed with AR due to increased clinical contact, which could inflate the observed HCRU after diagnosis.

## Conclusions

UK patients who have AR have decreased general quality-of-life and impose a burden on the UK healthcare system through increased GP usage, hospital visits, and medication usage. This usage may be driven by a subgroup of patients with more severe disease, based on the presence of a diagnosis code in hospital data, but further research is required to investigate the specific drivers of this trend. The study also found that asthma incidence was lower after AR diagnosis than before, suggesting that earlier diagnosis and management of AR may help reduce the occurrence and exacerbation of asthma. A considerable proportion of patients with AR presentation may be underdiagnosed, and improvements to the clinical management of these patients in the UK, including allergen avoidance programs with more proactive diagnosing, has the potential to decrease AR morbidity and offset this burden. In addition, referral timing can impact the timeliness of diagnosis and treatment and improving allergy testing and diagnosis at the primary care level may help reduce the severity of allergic disease and the incidence of asthma. More specific coding in primary care would also aid in defining this broad patient group and mitigate the risk of misclassification bias in future research.

## Data Availability

The datasets presented in this article are not readily available because raw data cannot be provided outside the Clinical Practice Research Datalink (CPRD) without suitable contracting terms in place. Requests to access the datasets should be directed to hilary.shepherd@mhra.gov.uk.

## References

[1] Akhouri S House SA. Allergic rhinitis. In: Statpearls. Treasure Island (FL): StatPearls Publishing (2025). [Published online July 16, 2023].

[2] Cleveland Clinic. Allergic Rhinitis (Hay Fever) (2023). Available online at: https://my.clevelandclinic.org/health/diseases/8622-allergic-rhinitis-hay-fever (Accessed September 21; Accessed June 8, 2025).

[3] Medical News Today. What to Know about the Triad of Asthma, Eczema, and Allergies (2021). Available online at: https://www.medicalnewstoday.com/articles/asthma-triad#summary (Accessed November 30; Accessed June 8, 2025),

[4] Association NE. The Atopic March: How Eczema Can Lead to Allergies and Asthma (2022). Available online at: https://nationaleczema.org/blog/science-atopic-march/ (Accessed September 28; Accessed June 8, 2025).

[5] Blaiss MS Hammerby E Robinson S Kennedy-Martin T Buchs S. The burden of allergic rhinitis and allergic rhinoconjunctivitis on adolescents: a literature review. Ann Allergy Asthma Immunol. (2018) 121(1):43–52 e3. 10.1016/j.anai.2018.03.028 29626629

[6] Wong QYA Lim JJ Ng JY Malipeddi P Lim YYE Sio YY The burden of allergic rhinitis is undermanaged in a large proportion of Chinese young adults from Singapore. World Allergy Organ J. (2024) 17(9):100954. 10.1016/j.waojou.2024.100954 39228765 PMC11367507

[7] National Institute for Care Excellence. Allergic Rhinitis: Scenario Management. Sheffield: Clarity Informatics Limited (trading as Agilio Software | Primary Care) (2024). Available online at: https://cks.nice.org.uk/topics/allergic-rhinitis/management/management/ (Accessed September 26, 2024).

[8] Price DB Smith PK Harvey RJ Carney AS Kritikos V Bosnic-Anticevich SZ Real-life treatment of rhinitis in Australia: a historical cohort study of prescription and over-the-counter therapies for patients with and without additional respiratory disease. Pragmat Obs Res. (2018) 9:43–54. 10.2147/POR.S153266 30147391 PMC6101013

[9] Grønhøj Larsen C Gyldenløve M Linneberg A. Allergic rhinitis is often undiagnosed and untreated: results from a general population study of Danish adults. Clin Respir J. (2013) 7(4):354–8. 10.1111/crj.12015 23362970

[10] British Medical Association. Nhs Backlog Data Analysis (2025). Available online at: https://www.bma.org.uk/advice-and-support/nhs-delivery-and-workforce/pressures/nhs-backlog-data-analysis (Accessed April 10).

[11] Bauchau V Durham SR. Prevalence and rate of diagnosis of allergic rhinitis in Europe. Eur Respir J. (2004) 24(5):758–64. 10.1183/09031936.04.00013904 15516669

[12] Licari A Magri P De Silvestri A Giannetti A Indolfi C Mori F Epidemiology of allergic rhinitis in children: a systematic review and meta-analysis. J Allergy Clin Immunol Pract. (2023) 11(8):2547–56. 10.1016/j.jaip.2023.05.016 37236349

[13] Scadding GK Kariyawasam HH Scadding G Mirakian R Buckley RJ Dixon T Bsaci guideline for the diagnosis and management of allergic and non-allergic rhinitis (revised edition 2017; first edition 2007). Clin Exp Allergy. (2017) 47(7):856–89. 10.1111/cea.12953 30239057

[14] Mazzotta P Loebstein R Koren G. Treating allergic rhinitis in pregnancy. Safety considerations. Drug Saf. (1999) 20(4):361–75. 10.2165/00002018-199920040-00005 10230583

[15] Pali-Schöll I Namazy J Jensen-Jarolim E. Allergic diseases and asthma in pregnancy, a secondary publication. World Allergy Organ J. (2017) 10(1):10. 10.1186/s40413-017-0141-8 28286601 PMC5333384

[16] [Dataset] Clinical Practice Research Datalink. Cprd Aurum June 2024 Dataset (2024). 10.48329/f5ek-pk69. Available online at: https://www.cprd.com/doi/cprd-aurum-june-2024-dataset (Accessed September 26, 2024).

[17] [Dataset] Clinical Practice Research Datalink. Cprd Aurum Hes Apc January 2022 (2022). 10.48329/vagx-9d96. Available online at: https://www.cprd.com/cprd-aurum-hes-apc-january-2022 (Accessed September 26, 2024).

[18] [Dataset] Clinical Practice Research Datalink. Cprd Aurum Hes op August 2021 (2021). 10.48329/7hm3-gt75HES. Available online at: https://www.cprd.com/cprd-aurum-hes-op-august-2021 (Accessed September 26, 2024).

[19] [Dataset] Clinical Practice Research Datalink. Cprd Aurum Small Area Data (Patient) January 2022 (2022). 10.48329/aytt-h222. Available online at: https://www.cprd.com/cprd-aurum-small-area-data-patient-january-2022 (Accessed September 26, 2024).

[20] deShazo RD Kemp SF. Patient Education: Allergic Rhinitis (Beyond the Basics) (2024). Available online at: https://www.uptodate.com/contents/allergic-rhinitis-beyond-the-basics (Accessed March 6, 2024; Accessed June 24, 2025).

[21] Harshfield A Abel GA Barclay S Payne RA. Do Gps accurately record date of death? A UK observational analysis. BMJ Support Palliat Care. (2020) 10(3):e24. 10.1136/bmjspcare-2018-001514 29950293

[22] Watt T Sullivan R Aggarwal A. Primary care and cancer: an analysis of the impact and inequalities of the COVID-19 pandemic on patient pathways. BMJ Open. (2022) 12(3):e059374. 10.1136/bmjopen-2021-059374 35332047 PMC8948073

[23] National Health Service. Early or Delayed Puberty (2022). Available online at: https://www.nhs.uk/conditions/early-or-delayed-puberty/ (Accessed September 6; Accessed June 24, 2025).

[24] Gov.Uk. Male and Female Populations (2023). Available online at: https://www.ethnicity-facts-figures.service.gov.uk/uk-population-by-ethnicity/demographics/male-and-female-populations/latest/ (Accessed August 2; Accessed June 8, 2025).

[25] . Ministry of Housing Communities and Local Government. English Indices of Deprivation 2019 (2019). Available online at: https://www.gov.uk/government/statistics/english-indices-of-deprivation-2019 (Accessed September 26; Accessed June 24, 2025).

[26] Hobbs FDR Bankhead C Mukhtar T Stevens S Perera-Salazar R Holt T Clinical workload in UK primary care: a retrospective analysis of 100 million consultations in England, 2007–14. Lancet. (2016) 387(10035):2323–30. 10.1016/S0140-6736(16)00620-6 27059888 PMC4899422

[27] Jick S Vasilakis-Scaramozza C Persson R Neasham D Kafatos G Hagberg KW. Use of the Cprd aurum database: insights gained from new data quality assessments. Clin Epidemiol. (2023) 15:1219–22. 10.2147/clep.S434832 38126004 PMC10732313

[28] Medicines & Healthcare products Regulatory Agency. Cprd Aurum Frequently Asked Questions (Faqs) (2023). (Accessed December 21; Accessed June 9, 2025).

[29] National Health Service. Nhs Vaccinations and When to Have Them (2023). Available online at: https://www.nhs.uk/vaccinations/nhs-vaccinations-and-when-to-have-them/ (Accessed August 9; Accessed June 9, 2025).

[30] BioSpace. Global Allergic Rhinitis Drugs Market 2019–2023| Increasing Demand for Over-the-Counter Medicines to Boost Growth | Technavio (2018). Available online at: https://www.biospace.com/global-allergic-rhinitis-drugs-market-2019-2023-increasing-demand-for-over-the-counter-medicines-to-boost-growth-technavio (Accessed December 14; Accessed June 8, 2025).

[31] Sarbacker GB. Updates in the management of seasonal allergic rhinitis. Updated July 14 (2024). Available online at: https://www.uspharmacist.com/article/updates-in-the-management-of-seasonal-allergic-rhinitis (Accessed September 26, 2024).

[32] Lipworth B Newton J Ram B Small I Schwarze J. An algorithm recommendation for the pharmacological management of allergic rhinitis in the UK: a consensus statement from an expert panel. NPJ Prim Care Respir Med. (2017) 27(1):3. 10.1038/s41533-016-0001-y 28115736 PMC5434768

[33] Persson R Jick S. Incomplete capture of apremilast in clinical practice research datalink aurum: an example of exposure misclassification of specialty treatments in United Kingdom general practice databases. Pharmacoepidemiol Drug Saf. (2024) 33(1):e5707. 10.1002/pds.5707 37786242

[34] Allergy & Asthma Associates of Southern California. Nonallergic Rhinitis and Vasomotor Rhinitis (2025). Available online at: https://www.socalallergy.com/nonallergic-rhinitis-and-vasomotor-rhinitis#:∼:text=Patients%20with%20VMR%20may%20have,well%20as%20for%20treatment%20options (Accessed September 12, 2025).

[35] Ryan D Grant-Casey J Scadding G Pereira S Pinnock H Sheikh A. Management of allergic rhinitis in UK primary care: baseline audit. Prim Care Respir J. (2005) 14(4):204–9. 10.1016/j.pcrj.2005.03.009 16701726 PMC6743577

[36] National Health Service Imperial College Healthcare. Allergy (2025). Available online at: https://www.imperial.nhs.uk/our-services/allergy#:∼:text=Before%20your%20appointment,What%20to%20bring (Accessed September 11, 2025).

[37] Library of Congress. United Kingdom: Social Media Advertisements for ‘Hay Fever Injections’ Violate Advertising Code (2022). Available online at: https://www.loc.gov/item/global-legal-monitor/2022-11-08/united-kingdom-social-media-advertisements-for-hay-fever-injections-violate-advertising-code/ (Accessed September 11, 2025).

[38] Remvig CL Diers CS Meteran H Thomsen SF Sigsgaard T Høj S YouTube as a source of (mis)Information on allergic rhinitis. Ann Allergy Asthma Immunol. (2022) 129(5):612–7. 10.1016/j.anai.2022.06.031 35843519

[39] Scadding GK Williams A. The burden of allergic rhinitis as reported by UK patients compared with their doctors. Updated June (2024). Available online at: https://www.rhinologyjournal.com/Abstract.php?id=689 (Accessed September 26, 2024). 18575009

[40] Novembre E Galli E Landi F Caffarelli C Pifferi M De Marco E Coseasonal sublingual immunotherapy reduces the development of asthma in children with allergic rhinoconjunctivitis. J Allergy Clin Immunol. (2004) 114(4):851–7. 10.1016/j.jaci.2004.07.012 15480326

[41] Whittaker H Kramer Fiala Machado A Hatam S Cook S Scully S Evans HTT Incidence and prevalence of asthma, chronic obstructive pulmonary disease and interstitial lung disease between 2004 and 2023: harmonised analyses of longitudinal cohorts across England, Wales, south-east Scotland and Northern Ireland. Thorax. (2025) 80(7):466–77. 10.1136/thorax-2024-222699 40199588 PMC12322415

[42] Wise SK Damask C Roland LT Ebert C Levy JM Lin S International consensus statement on allergy and rhinology: allergic rhinitis—2023. Int Forum Allergy Rhinol. (2023) 13(4):293–859. 10.1002/alr.23090 36878860

