# Supplementary material for: Burden of allergic rhinitis in the United Kingdom
Source: Front Allergy. 2025 Nov 4;6:1676574. doi: 10.3389/falgy.2025.1676574 (PMC12631609; doi:10.3389/falgy.2025.1676574)
Supplement: Supplementary file 1 [file Table1.docx]

MedCodeId Observations OriginalReadCode CleansedReadCode Term SnomedCTConceptId SnomedCTDescriptionId EmisCodeCategoryId

102311013 2000000 H17 H17..00 Allergic rhinitis 61582004 102311013 32

8031201000006110 100 ^ESCTAC803120 Acute exacerbation of asthma co-occurrent with allergic rhinitis 99031000119107 3040730014 32

8038931000006119 6 ^ESCTSE803893 Severe persistent asthma co-occurrent with allergic rhinitis 124991000119109 3038312015 32

8038941000006112 3 ^ESCTMO803894 Moderate persistent asthma co-occurrent with allergic rhinitis 125001000119103 3043951015 32

8038961000006111 90 ^ESCTMI803896 Mild persistent asthma co-occurrent with allergic rhinitis 125011000119100 3040894010 32

8038971000006116 80 ^ESCTIN803897 Intermittent asthma co-occurrent with allergic rhinitis 125021000119107 3041241019 32

9314961000006112 10 ^ESCTAC931496 Acute severe exacerbation of asthma co-occurrent with allergic rhinitis 10674711000119105 3043983014 32

9315111000006113 2 ^ESCTAC931511 Acute severe exacerbation of severe persistent asthma co-occurrent with allergic rhinitis 10675551000119104 3043762011 32

9315141000006112 1 ^ESCTSE931514 Severe persistent asthma controlled co-occurrent with allergic rhinitis 10675631000119109 3083256016 32

9315231000006113 10 ^ESCTAC931523 Acute severe exacerbation of mild persistent allergic asthma co-occurrent with allergic rhinitis 10675991000119100 3043192011 32

9315281000006114 100 ^ESCTMI931528 Mild persistent asthma controlled co-occurrent with allergic rhinitis 10676111000119102 3083630015 32

9315321000006115 2 ^ESCTMI931532 Mild persistent asthma uncontrolled co-occurrent with allergic rhinitis 10676231000119102 3083628017 32

9315391000006118 7 ^ESCTAC931539 Acute severe exacerbation of moderate persistent asthma co-occurrent with allergic rhinitis 10676511000119109 3043960011 32

9315441000006119 1 ^ESCTMO931544 Moderate persistent asthma uncontrolled co-occurrent with allergic rhinitis 10676671000119102 3083625019 32

7374141000006112 3 ^ESCTHO737414 House dust mite rhinitis 449729000 2913168016 15

224001000000114 300000 H17-1 H17..11 Perennial allergic rhinitis 446096008 2883237016 32

492457012 1000 ESCTSE4 Seasonal allergic rhinitis 367498001 1956781000006116 32

301824017 2000 Hyu21 Hyu2100 [X]Other allergic rhinitis 61582004 102311013 32

3500471000006116 30 ^ESCTAR350047 AR - Allergic rhinitis 61582004 1232224017 32

301823011 70000 Hyu20 Hyu2000 Seasonal allergic rhinitis 367498001 492457012 32

481104016 700000 H170 H170.00 Allergic rhinitis due to pollens 21719001 481104016 32

5035141000006112 300 ^ESCTPE503514 Perennial allergic rhinitis with seasonal variation 232353008 348111014 31

3994111000006116 4 ^ESCTAL399411 Allergic rhinitis due to grass pollen 91926002 3300292019 31

3994121000006112 2 ^ESCTAL399412 Allergic rhinitis caused by grass pollen 91926002 3300291014 31

3994131000006110 4 ^ESCTAL399413 Allergic rhinitis due to tree pollen 91927006 3300386011 31

3994161000006118 1 ^ESCTAL399416 Allergic rhinitis caused by weed pollen 91928001 3300288014 31

7099681000006119 1 ^ESCTAL709968 Allergic rhinitis due to animal dander 429195002 2692979010 15

7374131000006119 20 ^ESCTAL737413 Allergic rhinitis due to house dust mite 449729000 2913105016 15

8048311000006110 1 ^ESCTAL804831 Allergic rhinitis caused by animal hair and dander 156051000119109 3295017017 15

301268012 400000 H17z H17z.00 Allergic rhinitis NOS 61582004 102311013 32

348109017 500000 H171-4 H171.14 Allergic rhinitis due to pollen 21719001 36446019 32

396097015 30000 H171 H171.00 Allergic rhinitis due to other allergens 61582004 102311013 32

396098013 1000000 H172 H172.00 Allergic rhinitis due to allergen 61582004 499054013 32

2845671000006110 400 ^ESCTAL284567 Allergic rhinitis caused by pollens 21719001 3291353015 32

817341000006110 3000000 H170-1 H170.11 Hay fever - pollens 21719001 481104016 32
