# Supplementary material for: Burden of allergic rhinitis in the United Kingdom
Source: Front Allergy. 2025 Nov 4;6:1676574. doi: 10.3389/falgy.2025.1676574 (PMC12631609; doi:10.3389/falgy.2025.1676574)
Supplement: Supplementary file 2 [file Table2.docx]

icdcode id description modifier version

J30.1 J301 Allergic rhinitis due to pollen 201604

J30.2 J302 Other seasonal allergic rhinitis 201604

J30.3 J303 Other allergic rhinitis 201604

J30.4 J304 "Allergic rhinitis, unspecified" 201604

J45.0 J450 Predominantly allergic asthma 201604
