# Supplementary material for: Burden of allergic rhinitis in the United Kingdom
Source: Front Allergy. 2025 Nov 4;6:1676574. doi: 10.3389/falgy.2025.1676574 (PMC12631609; doi:10.3389/falgy.2025.1676574)
Supplement: Supplementary file 3 [file Table3.docx]

MedCodeId Observations OriginalReadCode CleansedReadCode Term SnomedCTConceptId SnomedCTDescriptionId EmisCodeCategoryId

2622113011 900000 SN530 SN53000 Allergic reaction 419076005 2577368014 15

789841000006110 10000 SN53-1 SN53.11 Allergic condition 473011001 2955675016 15

899941000006119 10000 SN53-99 SN53.99 Allergic reaction NOS 419076005 899941000006119 15

991171000006116 20 SN530-99 SN53099 Allergic reaction NOS 419076005 991171000006116 15

4637271000006115 300 ^ESCTAL463727 Allergic disorder initial assessment 170837001 264799016 1

4637291000006119 60 ^ESCTAL463729 Allergic disorder - symptom change 170839003 264801018 1

4637331000006114 20 ^ESCTAL463733 Allergic disorder treatment started 170842009 264805010 1

5169211000006118 200 ^ESCTAC516921 Acute allergic reaction 241929008 362147011 15

5192461000006112 8 ^ESCTAL519246 Allergic disorder monitoring status 243865006 364700014 31

5887221000006111 200 ^ESCTAL588722 Allergic cough 300959008 442163014 31

6920141000006110 2 ^ESCTAL692014 Allergic reaction to pollen 418364006 2577419013 15

6920171000006119 8 ^ESCTAL692017 Allergic reaction to grass pollen 418367004 2577417010 15

6921481000006117 1 ^ESCTAL692148 Allergic reaction to animal 418448002 2577434019 15

6921491000006119 1 ^ESCTAL692149 Allergic reaction caused by animal 418448002 3296990014 15

6929331000006112 2 ^ESCTAL692933 Allergic reaction to tree pollen 418943003 2577420019 15

6979571000006119 1 ^ESCTAT697957 Atopic IgE-mediated allergic disorder 421871004 2616600019 15

6983251000006118 10 ^ESCTIG698325 IgE-mediated allergic disorder 422076005 2616598013 15

6988151000006116 20 ^ESCTNO698815 Non-IgE-mediated allergic disorder 422339003 2616599017 15

7480561000006113 8 ^ESCTAL748056 Allergic process 472964009 2955500018 31

12086611000006115 3 ^ESCT1208661 IgE-mediated allergic process 769260004 3690603015 31

250146014 30000 12R 12R..00 FH: Allergy 160469004 250146014 7

251846018 1000000 14M 14M..00 H/O: non-drug allergy 161611007 251846018 15

442106016 60000 ESCTAL2 Allergy to pollen 300910009 442106016 15

1495072016 50000 H1710 H171000 Allergy to animal 717234006 3308773018 15

2577418017 40000 ESCTAL3 Allergy to grass pollen 418689008 2577418017 15

2577421015 10000 ESCTAL4 Allergy to tree pollen 419263009 2577421015 15

280451000006113 20000 EMISALLERGY Non-drug allergy 280451000006109 280451000006113 15

634501000000119 20000 H1711 H171100 Dog allergy 419271008 634501000000119 15

4532011000006112 30 ^ESCTFA453201 Family history of allergy 160469004 250147017 7

4532021000006116 2 ^ESCTFA453202 Family history: Allergy 160469004 2666806012 7

5035071000006117 600 ^ESCTAL503507 Allergy to animal dander 232347008 3305274012 15

5035091000006116 4 ^ESCTDA503509 Dander allergy 232347008 3305275013 15

5035121000006117 10000 ^ESCTAL503512 Allergy to house dust mite 232350006 3326799014 15

5886651000006119 80 ^ESCTAL588665 Allergy to animal hair 300911008 442107013 15

6345421000006110 3 ^ESCTVE634542 Verification of allergy status 370860007 1209589013 31

6516721000006117 200 ^ESCTDU651672 Dust allergy 390952000 1484982010 15

6762281000006114 10000 ^ESCTAL676228 Allergy 408439002 2163976014 37

6931201000006110 20 ^ESCTAL693120 Allergy to horse dander 419063004 2578542018 15

6933641000006111 2 ^ESCTAL693364 Allergy to weed pollen 419210001 2577424011 15

6934671000006117 7000 ^ESCTAL693467 Allergy to dog dander 419271008 2578488010 15

6937881000006114 20 ^ESCTAL693788 Allergy to mould 419474003 2578545016 15

6937891000006112 1 ^ESCTAL693789 Allergy to mold 419474003 2578544017 15

6979581000006116 2 ^ESCTAT697958 Atopic allergy 421871004 2622184011 15

7291301000006110 2 ^ESCTSE729130 Seasonal allergy 444316004 2841284012 15

7496401000006110 2000 ^ESCTAL749640 Allergy 609328004 2958622019 15

7584221000006117 3 ^ESCTCA758422 Cattle dander allergy 703935005 3011423015 15

12704901000006119 600 ^ESCT1270490 Allergy to cat dander 232346004 2838980018 15

13791691000006113 200 ^ESCT1379169 Allergy to house dust 232349006 3750284012 15

13791701000006113 500 ^ESCT1379170 Allergy to dust mite protein 232350006 3743586019 15

13821591000006119 50 ^ESCT1382159 Allergy to dust 390952000 3750252017 15

14751451000006114 20 ^ESCT1475145 Allergy to bird protein 1208804002 4971122016 15

14751521000006118 3000 ^ESCT1475152 Allergy to domestic cat protein 1208807009 4971136014 15

14751531000006115 200 ^ESCT1475153 Cat allergy 1208807009 4971133018 15

14751551000006110 1000 ^ESCT1475155 Allergy to domestic dog protein 1208808004 4971150016 15

14751571000006117 80 ^ESCT1475157 Dog allergy 1208808004 4971153019 15
