# Supplementary material for: Burden of allergic rhinitis in the United Kingdom
Source: Front Allergy. 2025 Nov 4;6:1676574. doi: 10.3389/falgy.2025.1676574 (PMC12631609; doi:10.3389/falgy.2025.1676574)
Supplement: Supplementary file 4 [file Table4.docx]

MedCodeId Observations OriginalReadCode CleansedReadCode Term SnomedCTConceptId SnomedCTDescriptionId EmisCodeCategoryId

1483199016 60000 H330-1 H330.11 Allergic asthma 389145006 1483199016 32

3514928013 2 ^ESCT1165693 Uncomplicated allergic asthma 735588005 3514928013 32

2884311000006118 7 ^ESCTAL288431 Allergic eczema 24079001 40427011 32

5028411000006110 9000 ^ESCTSE502841 Seasonal allergic conjunctivitis 231855007 347431016 32

5028431000006116 500 ^ESCTPE502843 Perennial allergic conjunctivitis 231856008 347432011 31

7015991000006110 20 ^ESCTNO701599 Non-IgE mediated allergic asthma 423889005 2644387016 32

7030311000006117 700 ^ESCTIG703031 IgE-mediated allergic asthma 424643009 2644386013 32

7030341000006118 1 ^ESCTIG703034 IgE mediated allergic asthma 424643009 2648328017 32

7488441000006110 10000 ^ESCTAL748844 Allergic conjunctivitis 473460002 2957165016 32

13997031000006117 300 ^ESCT1399703 Intermittent allergic asthma 10674991000119104 3777826016 32
