# Supplementary material for: Burden of allergic rhinitis in the United Kingdom
Source: Front Allergy. 2025 Nov 4;6:1676574. doi: 10.3389/falgy.2025.1676574 (PMC12631609; doi:10.3389/falgy.2025.1676574)
Supplement: Supplementary file 5 [file Table5.docx]

MedCodeId Observations OriginalReadCode CleansedReadCode Term SnomedCTConceptId SnomedCTDescriptionId EmisCodeCategoryId

5259491000006115 90 ^ESCTCL525949 Clearing throat - hawking 248589007 371069017 31

4077821000006111 300 ^ESCTCO407782 Congestion of throat 102617004 252336014 31

2694881000006114 30 ^ESCTTH269488 Throat clearing 12239004 21076011 31

253191016 70000 1C5 1C5..00 Sneezing symptoms 162367006 253191016 27

253194012 10000 1C5Z 1C5Z.00 Sneezing symptom 162367006 2575641015 27

317323015 20000 R04z3 R04z300 Sneezing 76067001 126342013 27

503186018 5000 1C52 1C52.00 Sneezes 76067001 503186018 27

113345017 800000 H1y1z-2 H1y1z12 Nasal congestion 68235000 113345017 32

136509019 300000 H1y1z-3 H1y1z13 Sinus congestion 82297005 136509019 32

961831000006111 3000 EMISCRH1 Rhinorrhea/nasal congestion 961831000006107 961831000006111 27

3838761000006113 7000 ^ESCTCO383876 Congestion of nasal sinus 82297005 136506014 32

5532911000006115 4000 ^ESCTCO553291 Complaining of nasal congestion 272034001 2669552013 27

407076010 100000 1C82-1 1C82.11 C/O nasal congestion 272034001 407076010 27

142764012 1000000 H120 H120.00 Chronic rhinitis 86094006 142764012 32

255579018 30000 2D22 2D22.00 O/E - rhinorrhoea 164184005 255579018 27

301187012 100000 H120z H120z00 Chronic rhinitis NOS 86094006 142764012 32

2161421018 100000 1C83-3 1C83.13 Rhinorrhoea 64531003 2161421018 32

162421000006116 200000 H00-6 H00..16 Acute rhinitis 82272006 136468011 32

531231000006113 400000 1C84 1C84.00 C/O - postnasal drip 162382001 253217011 27

3732451000006116 2000 ^ESCTPO373245 Postnasal drip 75803007 125902019 27

3732491000006110 100 ^ESCTPO373249 Postnasal catarrh 75803007 503125017 27

4548241000006116 20000 ^ESCTCO454824 Complaining of postnasal drip 162382001 2667946014 27

125901014 80000 2D26 2D26.00 Postnasal discharge 75803007 125901014 27

5571551000006118 200 ^ESCTSN557155 Sniffles 275280004 411243015 31

1777356018 100000 Q31y5-2 Q31y512 Snuffles 397746007 1777356018 32

3549411000006113 8 ^ESCTNA354941 Nasal catarrh 64531003 2164102016 32

5270691000006112 200 ^ESCTSI527069 Sinus catarrh 249369003 372078013 31

9300010 10000 1BA9 1BA9.00 Sinus headache 4969004 9300010 27

142901000006114 70000 1B1G0 1B1G000 Sinus headache 4969004 9300010 27

217741000000112 300000 1BA5-1 1BA5.11 Sinus headache 4969004 9300010 27

1819481000006119 2000 EMISNQNA10 Nasal sinus problem 301202006 442459011 27

2578701000006119 2 ^ESCTSI257870 Sinus pain 4969004 9301014 27

5892621000006114 400 ^ESCTMA589262 Maxillary sinus pain 301356002 442639017 31

5892631000006112 400 ^ESCTFR589263 Frontal sinus pain 301357006 442640015 31

253225013 500000 1C92 1C92.00 Has a sore throat 162388002 253225013 27

253227017 200000 1C9Z 1C9Z.00 Sore throat symptom NOS 267102003 398001015 27

398001015 8000000 1C9 1C9..00 Sore throat symptom 267102003 398001015 27

411476019 20000 H121-1 H121.11 Sore throat - chronic 275488008 411476019 27

411477011 50000 1C93 1C93.00 Persistent sore throat 275488008 411477011 27

139761000006116 2000000 H02-1 H02..11 Sore throat 162397003 2164213014 27

2500691000006110 20 ^ESCTCH250069 Chronic sore throat 140004 1285015 32

4548281000006110 1 ^ESCTSO454828 Sore throat present 162388002 1494838019 27

82824016 20000000 171 171..00 Cough 49727002 82824016 27

113213012 200000 171A 171A.00 Chronic cough 68154008 113213012 27

252351017 600000 1713 1713.00 Productive cough -clear sputum 161923004 252351017 27

252352012 2000000 1714 1714.00 Productive cough -green sputum 161924005 252352012 27

252353019 800000 1715 1715.00 Productive cough-yellow sputum 161925006 252353019 27

252366019 300000 171Z 171Z.00 Cough symptom NOS 272039006 407081018 27

252406018 900000 173B 173B.00 Nocturnal cough / wheeze 161947006 252406018 27

317403015 2000000 R062 R062.00 [D]Cough 49727002 82824016 27

397882011 900000 1716 1716.00 Productive cough 28743005 48123018 27

423230012 1000000 171B 171B.00 Persistent cough 284523002 423230012 27

407081018 5000000 171-1 171..11 C/O - cough 272039006 407081018 27

442503015 5000 171H 171H.00 Difficulty in coughing up sputum 301245004 442503015 27

459738011 5000 171E 171E.00 Unexplained cough 315246003 459738011 27

598431000006113 200000 1716-1 1716.11 Coughing up phlegm 28743005 48123018 27

961781000006119 1000 EMISCCO10 Cough 961781000006103 961781000006119 27

1780331000006117 20000 EMISNQDA11 Daytime cough 1780331000006101 1780331000006117 27

5243651000006110 200 ^ESCTPA524365 Painful cough 247410004 369436013 31

5468241000006113 200 ^ESCTCO546824 Coughing 263731006 392016010 31

5533011000006112 70000 ^ESCTCO553301 Complaining of cough 272039006 2669603018 27

5887221000006111 200 ^ESCTAL588722 Allergic cough 300959008 442163014 31

1805221000006118 2000 EMISNQHA45 Has an itchy nose 1805221000006102 1805221000006118 27

253084017 100000 1B86 1B86.00 Has an itchy eye 162288000 253084017 27

3715481000006112 20 ^ESCTIT371548 Itching of eye 74776002 124184013 31

13715891000006111 3 ^ESCT1371589 Itching of both eyes 16227811000119102 3772834019 31

3639421000006110 300 ^ESCTIR363942 Irritation of nose 70076002 116383010 27

253220015 100000 1C8Z 1C8Z.00 Nasal symptom 249307003 2470117011 27

371996016 400000 1C8-1 1C8..11 Nasal symptoms 249307003 371996016 27

397996018 200000 1C8 1C8..00 Nasal symptoms OS 249306007 371995017 27

442455017 2000 ESCTNA1 Nasal problem 301199001 442455017 27

681561000006119 100000 H00-4 H00..14 Nasal catarrh - acute 64531003 2161421018 32

2808671000006111 20 ^ESCTNA280867 Nasal mucosa oedematous 19452008 479522018 31

2808681000006114 1 ^ESCTNA280868 Nasal mucosa edematous 19452008 479521013 31

3736841000006116 5 ^ESCTNA373684 Nasal mucus 76044003 126305018 31

5298791000006113 3 ^ESCTNA529879 Nasal pressure 251365002 374536010 31

397988011 100000 1B87-3 1B87.13 Watery eyes 420103007 2576579010 27

397991011 100000 1B87 1B87.00 Watery eye 420103007 2576579010 27

407074013 40000 1B87-2 1B87.12 C/O - watering eyes 272032002 407074013 27

5532881000006115 1000 ^ESCTCO553288 Complaining of watering eyes 272032002 2669551018 27

6948271000006110 20 ^ESCTWA694827 Watering eye 420103007 2579717017 27

298926010 50000 F4Kz4 F4Kz400 Red eye 75705005 503096013 32

397987018 200000 1B85 1B85.00 Has a red eye 267093002 397987018 27

183471000006110 100000 F4Kz4-1 F4Kz411 Red eye NOS 75705005 503096013 32

183481000006113 300000 1B85-1 1B85.11 Red eye 703630003 3009212011 27

9478871000006115 20 ^ESCTBI947887 Bilateral red eyes 12241791000119109 3332280013 31

253247013 60000 1CB5 1CB5.00 Throat irritation 162400007 253247013 27
