# Supplementary material for: Burden of allergic rhinitis in the United Kingdom
Source: Front Allergy. 2025 Nov 4;6:1676574. doi: 10.3389/falgy.2025.1676574 (PMC12631609; doi:10.3389/falgy.2025.1676574)
Supplement: Supplementary file 6 [file Table6.docx]

MedCodeId Observations OriginalReadCode CleansedReadCode Term SnomedCTConceptId SnomedCTDescriptionId EmisCodeCategoryId

4724551000006113 100000 ^ESCTRE472455 Referral to ENT service 183544005 283642016 40

5940191000006118 20 ^ESCTAD594019 Admission to ENT department 305417004 447836015 31

283573011 50000 8H3E 8H3E.00 Non-urgent ENT admission 183496005 283573011 40

285215012 8000000 9N1L 9N1L.00 Seen in ENT clinic 185223006 285215012 37

740871000006115 20000 8HMJ 8HMJ.00 Listed for ENT admission 183785003 283978012 40

1696151000006117 10000 EMISNQRE97 Referral to ENT nurse specialist 1696151000006101 1696151000006117 40

5959081000006113 500 ^ESCTDI595908 Discharge from ENT service 306657002 449754016 31

14430871000006115 20000 ^ESCT1443087 Referral to paediatric ENT (ear, nose and throat) service 1144648000 4543047016 32

283643014 7000000 8H53 8H53.00 ENT referral 183544005 283643014 40

284086016 300000 8HV2 8HV2.00 Private referral to ENT surgeon 183880002 284086016 40

449244019 10000 ESCTRE6 Referral to clinical allergist 306272008 449244019 40

451834015 50000 8H5R 8H5R.00 Referral to ear, nose and throat surgeon 308480005 451834015 40

2203181000000110 30000 8T02 8T02.00 Referral to paediatric ear, nose and throat service 850231000000105 2203181000000110 32

14430861000006110 5000 ^ESCT1443086 Referral to paediatric ear, nose and throat service 1144648000 4542705017 32

448999010 222676 8Hld 8Hld.00 Referral to clinical allergy service 306111000 448999010 40

5938661000006118 12 ^ESCTAD593866 Admission by ENT surgeon 305302002 447683016 31

5949081000006110 9 ^ESCTRE594908 Referral by ENT surgeon 306007009 448734014 31

5949091000006113 4 ^ESCTRE594909 Referral from ENT surgeon 306007009 448735010 31

5956671000006111 176 ^ESCTDI595667 Discharge by ENT surgeon 306471003 449505014 31
