# Supplementary material for: Burden of allergic rhinitis in the United Kingdom
Source: Front Allergy. 2025 Nov 4;6:1676574. doi: 10.3389/falgy.2025.1676574 (PMC12631609; doi:10.3389/falgy.2025.1676574)
Supplement: Supplementary file 7 [file Table7.docx]

J00-J06 Acute upper respiratory infections

J30-J39 Other diseases of upper respiratory tract

J40-J4A Chronic lower respiratory diseases

J64 Unspecified pneumoconiosis

J66 Airway disease due to specific organic dust

J67 Hypersensitivity pneumonitis due to organic dust

J68 Respiratory conditions due to inhalation of chemicals, gases, fumes and vapors

J70.8 Respiratory conditions due to other specified external agents

J70.9 Respiratory conditions due to unspecified external agent

J80 Acute respiratory distress syndrome

J96-J99 Other diseases of the respiratory system

R04 Hemorrhage from respiratory passages

R05 Cough

R06 Abnormalities of breathing

R07 Pain in throat and chest

T78.2 Anaphylactic shock, unspecified

T78.3 Angioneurotic edema

T78.4 Other and unspecified allergy
