# Supplementary material for: Burden of allergic rhinitis in the United Kingdom
Source: Front Allergy. 2025 Nov 4;6:1676574. doi: 10.3389/falgy.2025.1676574 (PMC12631609; doi:10.3389/falgy.2025.1676574)
Supplement: Supplementary file 8 [file Table8.docx]

ProdCodeId DMDCode TermfromEMIS ProductName drugsubstancename substancestrength formulation routeofadministration bnfcode DrugIssues

1558341000033116 218111000001106 Zirtek 10mg tablets (UCB Pharma Ltd) Zirtek 10mg tablets Cetirizine hydrochloride 10.000mg Tablet Oral 400000

2752841000033112 513911000001105 Cetirocol 10mg tablets (Teva UK Ltd) Cetirocol 10mg tablets Cetirizine hydrochloride 10.000mg Tablet Oral 70

2864141000033115 88211000001106 Zirtek Allergy 10mg tablets (UCB Pharma Ltd) Zirtek Allergy 10mg tablets Cetirizine hydrochloride 10.000mg Tablet Oral 200000

2905541000033116 4502411000001100 Zirtek Allergy 1mg/ml oral solution (UCB Pharma Ltd) Zirtek Allergy 1mg/ml oral solution Cetirizine hydrochloride 1.000mg/1.000ml Oral solution Oral 20000

2968441000033112 692911000001107 Piriteze Allergy 10mg tablets (Haleon UK Ltd) Piriteze Allergy 10mg tablets Cetirizine hydrochloride 10.000mg Tablet Oral 5000

3077741000033112 7735711000001104 Piriteze Allergy 1mg/ml syrup (Haleon UK Ltd) Piriteze Allergy 1mg/ml syrup Cetirizine hydrochloride 1.000mg/1.000ml Oral solution Oral 5000

3078241000033117 4494011000001105 Benadryl Allergy 1mg/ml oral solution (McNeil Products Ltd) Benadryl Allergy 1mg/ml oral solution Cetirizine hydrochloride 1.000mg/1.000ml Oral solution Oral 4000

4460241000033118 11493011000001100 Pollenshield Hayfever Relief 10mg tablets (Accord-UK Ltd) Pollenshield Hayfever Relief 10mg tablets Cetirizine hydrochloride 10.000mg Tablet Oral 100

4460341000033111 12430011000001100 Pollenshield Hayfever 10mg tablets (Accord-UK Ltd) Pollenshield Hayfever 10mg tablets Cetirizine hydrochloride 10.000mg Tablet Oral 70

8887341000033119 20344111000001109 Benadryl Allergy Liquid Release 10mg capsules (McNeil Products Ltd) Benadryl Allergy Liquid Release 10mg capsules Cetirizine hydrochloride 10.000mg Capsule Oral 1000

2644241000033110 3603411000001100 Xyzal 5mg tablets (UCB Pharma Ltd) Xyzal 5mg tablets Levocetirizine dihydrochloride 5.000mg Tablet Oral 80000

4187441000033115 11760111000001104 Xyzal 0.5mg/ml oral solution (UCB Pharma Ltd) Xyzal 0.5mg/ml oral solution Levocetirizine dihydrochloride 500.000microgram/1.000ml Oral solution Oral 1000

96341000033114 3232111000001105 Avomine 25mg tablets (Manx Healthcare Ltd) Avomine 25mg tablets Promethazine teoclate 25.000mg Tablet Oral 50000

1071241000033117 134411000001102 Phenergan 5mg/5ml elixir (Sanofi Consumer Healthcare) Phenergan 5mg/5ml elixir Promethazine hydrochloride 1.000mg/1.000ml Oral solution Oral 200000

1079441000033117 547211000001103 Phenergan 10mg tablets (Sanofi Consumer Healthcare) Phenergan 10mg tablets Promethazine hydrochloride 10.000mg Tablet Oral 90000

1079541000033116 351811000001108 Phenergan 25mg tablets (Sanofi Consumer Healthcare) Phenergan 25mg tablets Promethazine hydrochloride 25.000mg Tablet Oral 200000

1080741000033112 227211000001108 Phenergan Nightime 25mg tablets (Sanofi Consumer Healthcare) Phenergan Nightime 25mg tablets Promethazine hydrochloride 25.000mg Tablet Oral 4000

1367741000033112 4214611000001101 Sominex 20mg tablets (Teva UK Ltd) Sominex 20mg tablets Promethazine hydrochloride 20.000mg Tablet Oral 1000

14052341000033112 40822911000001101 Promethazine hydrochloride 25mg tablets (Imported) Promethazine hydrochloride 25mg tablets Promethazine hydrochloride 25.000mg Tablet 20

1427441000033118 58911000001105 Telfast 120mg tablets (Sanofi) Telfast 120mg tablets Fexofenadine hydrochloride 120.000mg Tablet Oral 200000

1427541000033117 880711000001100 Telfast 180mg tablets (Sanofi) Telfast 180mg tablets Fexofenadine hydrochloride 180.000mg Tablet Oral 100000

2956841000033117 4722611000001106 Telfast 30mg tablets (Sanofi) Telfast 30mg tablets Fexofenadine hydrochloride 30.000mg Tablet Oral 6000

284641000033118 3327811000001109 Clarityn 5mg/5ml syrup (Schering-Plough Ltd) Clarityn 5mg/5ml syrup Loratadine 1.000mg/1.000ml Oral solution Oral 90000

3934541000033117 8969911000001107 Hay-Rite Allergy 10mg tablets (Teva UK Ltd) Hay-Rite Allergy 10mg tablets Loratadine 10.000mg Tablet Oral 5

11899641000033114 20984011000001105 Clarityn Rapide Allergy 10mg tablets (Bayer Plc) Clarityn Rapide Allergy 10mg tablets Loratadine 10.000mg Oral lyophilisate Oral 5000

2644141000033115 42192511000001103 Levocetirizine 5mg tablets Levocetirizine 5mg tablets Levocetirizine dihydrochloride 5.000mg Tablet Oral 3040100 1000000

4187341000033114 11763111000001106 Levocetirizine 500micrograms/ml oral solution sugar free Levocetirizine 500micrograms/ml oral solution sugar free Levocetirizine dihydrochloride 500.000microgram/1.000ml Oral solution Oral 3040100 10000

230241000033118 37082911000001103 Cetirizine 1mg/ml oral solution sugar free Cetirizine 1mg/ml oral solution sugar free Cetirizine hydrochloride 1.000mg/1.000ml Oral solution Oral 3040100 4000000

235541000033112 42186511000001101 Cetirizine 10mg tablets Cetirizine 10mg tablets Cetirizine hydrochloride 10.000mg Tablet Oral 3040100 30000000

8887241000033112 20344911000001107 Cetirizine 10mg capsules Cetirizine 10mg capsules Cetirizine hydrochloride 10.000mg Capsule Oral 3040100 60000

1114141000033111 38753911000001106 Promethazine 5mg/5ml oral solution sugar free Promethazine 5mg/5ml oral solution sugar free Promethazine hydrochloride 1.000mg/1.000ml Oral solution Oral 3040100 400000

1134541000033111 42040511000001104 Promethazine hydrochloride 10mg tablets Promethazine hydrochloride 10mg tablets Promethazine hydrochloride 10.000mg Tablet Oral 3040100 900000

1134641000033112 42040711000001109 Promethazine hydrochloride 25mg tablets Promethazine hydrochloride 25mg tablets Promethazine hydrochloride 25.000mg Tablet Oral 3040100 4000000

2922241000033119 42040611000001100 Promethazine hydrochloride 20mg tablets Promethazine hydrochloride 20mg tablets Promethazine hydrochloride 20.000mg Tablet Oral 3040100 50000

572741000033119 42189111000001102 Fexofenadine 120mg tablets Fexofenadine 120mg tablets Fexofenadine hydrochloride 120.000mg Tablet Oral 3040100 6000000

572941000033116 42189211000001108 Fexofenadine 180mg tablets Fexofenadine 180mg tablets Fexofenadine hydrochloride 180.000mg Tablet Oral 3040100 8000000

2956741000033110 42189311000001100 Fexofenadine 30mg tablets Fexofenadine 30mg tablets Fexofenadine hydrochloride 30.000mg Tablet Oral 3040100 200000

13735441000033111 15774311000001100 Fexofenadine 180mg/5ml oral suspension Fexofenadine 180mg/5ml oral suspension Fexofenadine hydrochloride 36.000mg/1.000ml Oral suspension Oral 3040100 600

849641000033117 38896711000001106 Loratadine 5mg/5ml oral solution Loratadine 5mg/5ml oral solution Loratadine 1.000mg/1.000ml Oral solution Oral 3040100 2000000

852541000033112 42192711000001108 Loratadine 10mg tablets Loratadine 10mg tablets Loratadine 10.000mg Tablet Oral 3040100 10000000

11899541000033113 21014411000001100 Loratadine 10mg oral lyophilisates sugar free Loratadine 10mg oral lyophilisates sugar free Loratadine 10.000mg Oral lyophilisate Oral 3040100 3000

13000541000033113 36904111000001102 Loratadine 5mg/5ml oral solution sugar free Loratadine 5mg/5ml oral solution sugar free Loratadine 1.000mg/1.000ml Oral solution Oral 3040100 20000

3109641000033112 42040811000001101 Promethazine teoclate 25mg tablets Promethazine teoclate 25mg tablets Promethazine teoclate 25.000mg Tablet Oral 4060000 100000

157441000033118 35911911000001102 Brompheniramine 2mg/5ml oral solution Brompheniramine 2mg/5ml oral solution Brompheniramine maleate 400.000microgram/1.000ml Oral solution Oral 3040100 100000

442941000033111 15811000001101 Dimotane 2mg/5ml elixir (Mercury Pharma Group Ltd) Dimotane 2mg/5ml elixir Brompheniramine maleate 400.000microgram/1.000ml Oral solution Oral 70000

450941000033113 53011000001104 Dimotane Plus oral solution (Mercury Pharma Group Ltd) Dimotane Plus oral solution Brompheniramine maleate/ Pseudoephedrine hydrochloride 800.000microgram/1.000ml + 6.000mg/1.000ml Oral solution Oral 200000

456341000033111 107111000001105 Dimotane Plus Paediatric elixir (Mercury Pharma Group Ltd) Dimotane Plus Paediatric elixir Brompheniramine maleate/ Pseudoephedrine hydrochloride 400.000microgram/1.000ml + 3.000mg/1.000ml Oral solution Oral 200000

2261441000033117 42187611000001100 Desloratadine 5mg tablets Desloratadine 5mg tablets Desloratadine 5.000mg Tablet Oral 3040100 4000000

2261541000033116 69711000001105 Neoclarityn 5mg tablets (Organon Pharma (UK) Ltd) Neoclarityn 5mg tablets Desloratadine 5.000mg Tablet Oral 400000

2753041000033114 282711000001107 Neoclarityn 2.5mg/5ml syrup (Schering-Plough Ltd) Neoclarityn 2.5mg/5ml syrup Desloratadine 500.000microgram/1.000ml Oral solution Oral 20000

4811041000033113 16098011000001104 Neoclarityn 2.5mg/5ml oral solution (Organon Pharma (UK) Ltd) Neoclarityn 2.5mg/5ml oral solution Desloratadine 500.000microgram/1.000ml Oral solution Oral 8000

5129441000033113 3344611000001102 Desloratadine 2.5mg/5ml oral solution Desloratadine 2.5mg/5ml oral solution Desloratadine 500.000microgram/1.000ml Oral solution Oral 3040100 20000

5316841000033117 16101011000001100 Desloratadine 2.5mg/5ml oral solution sugar free Desloratadine 2.5mg/5ml oral solution sugar free Desloratadine 500.000microgram/1.000ml Oral solution Oral 3040100 60000

7740441000033110 20343311000001109 Midetorin 2.5mg/5ml oral solution (Actavis UK Ltd) Midetorin 2.5mg/5ml oral solution Desloratadine 500.000microgram/1.000ml Oral solution Oral 20

272841000033117 39699811000001103 Clemastine 500micrograms/5ml oral solution sugar free Clemastine 500micrograms/5ml oral solution sugar free Clemastine hydrogen fumarate 100.000microgram/1.000ml Oral solution Oral 3040100 30000

285641000033119 39688911000001101 Clemastine 1mg tablets Clemastine 1mg tablets Clemastine hydrogen fumarate 1.000mg Tablet Oral 3040100 70000

1404541000033117 3650911000001108 Tavegil 500micrograms/5ml elixir (Novartis Consumer Health UK Ltd) Tavegil 500micrograms/5ml elixir Clemastine hydrogen fumarate 100.000microgram/1.000ml Oral solution Oral 10000

1408941000033117 3650211000001104 Tavegil 1mg tablets (Thornton & Ross Ltd) Tavegil 1mg tablets Clemastine hydrogen fumarate 1.000mg Tablet Oral 20000

3841000033110 42184011000001104 Acrivastine 8mg capsules Acrivastine 8mg capsules Acrivastine 8.000mg Capsule Oral 3040100 500000

98541000033115 35905911000001102 Azatadine 500micrograms/5ml oral solution Azatadine 500micrograms/5ml oral solution Azatadine maleate 100.000microgram/1.000ml Oral solution Oral 3040100 3000

394641000033112 42187511000001104 Cyproheptadine 4mg tablets Cyproheptadine 4mg tablets Cyproheptadine hydrochloride 4.000mg Tablet Oral 3040100 40000

462741000033116 42188011000001108 Diphenhydramine 25mg tablets Diphenhydramine 25mg tablets Diphenhydramine hydrochloride 25.000mg Tablet Oral 3040100 100000

741541000033119 36057911000001106 Hydroxyzine 10mg/5ml oral solution Hydroxyzine 10mg/5ml oral solution Hydroxyzine hydrochloride 2.000mg/1.000ml Oral solution Oral 3040100 100000

745241000033119 42191311000001100 Hydroxyzine 10mg tablets Hydroxyzine 10mg tablets Hydroxyzine hydrochloride 10.000mg Tablet Oral 3040100 800000

745341000033112 42191411000001107 Hydroxyzine 25mg tablets Hydroxyzine 25mg tablets Hydroxyzine hydrochloride 25.000mg Tablet Oral 3040100 2000000

797541000033117 39696911000001104 Ketotifen 1mg capsules Ketotifen 1mg capsules Ketotifen fumarate 1.000mg Capsule Oral 3040100 9000

798341000033111 4030311000001100 Ketotifen 1mg/5ml oral solution sugar free Ketotifen 1mg/5ml oral solution sugar free Ketotifen fumarate 200.000microgram/1.000ml Oral solution Oral 3040100 40000

803841000033110 39697111000001104 Ketotifen 1mg tablets Ketotifen 1mg tablets Ketotifen fumarate 1.000mg Tablet Oral 3040100 40000

1417441000033112 4558711000001101 Terfenadine 120mg tablets Terfenadine 120mg tablets Terfenadine 120.000mg Tablet Oral 3040100 100000

1426941000033114 42196711000001107 Terfenadine 60mg tablets Terfenadine 60mg tablets Terfenadine 60.000mg Tablet Oral 3040100 1000000

1656841000033117 39108011000001100 Mizolastine 10mg modified-release tablets Mizolastine 10mg modified-release tablets Mizolastine 10.000mg Modified-release tablet Oral 3040100 300000

2189941000033111 42188111000001109 Diphenhydramine 50mg tablets Diphenhydramine 50mg tablets Diphenhydramine hydrochloride 50.000mg Tablet Oral 3040100 80000

2929541000033114 35931511000001104 Promethazine 25mg/1ml solution for injection ampoules Promethazine 25mg/1ml solution for injection ampoules Promethazine hydrochloride 25.000mg/1.000ml Solution for injection Intravenous/ Intramuscular 3040100 900

3085841000033117 36137711000001100 Chlorphenamine 10mg/1ml solution for injection ampoules Chlorphenamine 10mg/1ml solution for injection ampoules Chlorphenamine maleate 10.000mg/1.000ml Solution for injection Intravenous/ Intramuscular/ Subcutaneous 3040100 10000

3085941000033113 37083011000001106 Chlorphenamine 2mg/5ml oral solution Chlorphenamine 2mg/5ml oral solution Chlorphenamine maleate 400.000microgram/1.000ml Oral solution Oral 3040100 3000000

3086041000033115 37083111000001107 Chlorphenamine 2mg/5ml oral solution sugar free Chlorphenamine 2mg/5ml oral solution sugar free Chlorphenamine maleate 400.000microgram/1.000ml Oral solution Oral 3040100 500000

3086141000033116 39708211000001102 Chlorphenamine 4mg tablets Chlorphenamine 4mg tablets Chlorphenamine maleate 4.000mg Tablet Oral 3040100 5000000

3100141000033110 35897011000001101 Alimemazine 7.5mg/5ml oral solution Alimemazine 7.5mg/5ml oral solution Alimemazine tartrate 1.500mg/1.000ml Oral solution Oral 3040100 200000

3100241000033115 35896911000001100 Alimemazine 30mg/5ml oral solution Alimemazine 30mg/5ml oral solution Alimemazine tartrate 6.000mg/1.000ml Oral solution Oral 3040100 90000

3100341000033113 42184211000001109 Alimemazine 10mg tablets Alimemazine 10mg tablets Alimemazine tartrate 10.000mg Tablet Oral 3040100 300000

4020341000033111 36069711000001107 Diphenhydramine 10mg/5ml oral solution Diphenhydramine 10mg/5ml oral solution Diphenhydramine hydrochloride 2.000mg/1.000ml Oral solution Oral 3040100 600

5101141000033110 15859211000001108 Rupatadine 10mg tablets Rupatadine 10mg tablets Rupatadine fumarate 10.000mg Tablet Oral 3040100 20000

6161541000033115 18759311000001105 Bilastine 20mg tablets Bilastine 20mg tablets Bilastine 20.000mg Tablet Oral 3040100 3000

10268041000033113 42195511000001109 Pseudoephedrine 60mg / Acrivastine 8mg capsules Pseudoephedrine 60mg / Acrivastine 8mg capsules Acrivastine/ Pseudoephedrine hydrochloride 8.000mg + 60.000mg Capsule Oral 3040100 5000

12880341000033112 12635811000001103 Hydroxyzine 2.5mg/5ml oral solution Hydroxyzine 2.5mg/5ml oral solution Hydroxyzine hydrochloride 500.000microgram/1.000ml Oral solution Oral 3040100 200

12987641000033114 36786911000001101 Rupatadine 1mg/ml oral solution Rupatadine 1mg/ml oral solution Rupatadine fumarate 1.000mg/1.000ml Oral solution Oral 3040100 30

13133141000033113 35577711000001105 Hydroxyzine 10mg/5ml oral suspension Hydroxyzine 10mg/5ml oral suspension Hydroxyzine hydrochloride 2.000mg/1.000ml Oral suspension Oral 3040100 400

13738841000033117 39046011000001102 Bilastine 10mg orodispersible tablets sugar free Bilastine 10mg orodispersible tablets sugar free Bilastine 10.000mg Orodispersible tablet Oral 3040100 10

13739041000033116 39046111000001101 Bilastine 2.5mg/ml oral solution sugar free Bilastine 2.5mg/ml oral solution sugar free Bilastine 2.500mg/1.000ml Oral solution Oral 3040100 1

13779241000033114 39355711000001107 Alimemazine 30mg/5ml oral solution sugar free Alimemazine 30mg/5ml oral solution sugar free Alimemazine tartrate 6.000mg/1.000ml Oral solution Oral 3040100 200

13779341000033116 39355811000001104 Alimemazine 7.5mg/5ml oral solution sugar free Alimemazine 7.5mg/5ml oral solution sugar free Alimemazine tartrate 1.500mg/1.000ml Oral solution Oral 3040100 600

13841641000033114 39608811000001107 Alimemazine 10mg/5ml oral solution sugar free Alimemazine 10mg/5ml oral solution sugar free Alimemazine tartrate 2.000mg/1.000ml Oral solution Oral 3040100 300

821141000033112 42192411000001102 Levocabastine 0.5mg/ml eye drops Levocabastine 0.5mg/ml eye drops Levocabastine hydrochloride 500.000microgram/1.000ml Eye drops Ocular 11040200 10000

823641000033118 3875811000001101 Levocabastine 0.05% nasal spray Levocabastine 0.05% nasal spray Levocabastine hydrochloride 500.000microgram/1.000ml Spray Nasal 12020100 4000

828941000033117 3772711000001103 Livostin 0.5mg/ml eye drops (Novartis Ophthalmics) Livostin 0.5mg/ml eye drops Levocabastine hydrochloride 500.000microgram/1.000ml Eye drops Ocular 7000

833241000033119 3872111000001101 Livostin 0.05% nasal spray (Novartis Pharmaceuticals UK Ltd) Livostin 0.05% nasal spray Levocabastine hydrochloride 500.000microgram/1.000ml Spray Nasal 3000

3154741000033112 3772511000001108 Livostin Direct 0.5mg/ml eye drops (McNeil Products Ltd) Livostin Direct 0.5mg/ml eye drops Levocabastine hydrochloride 500.000microgram/1.000ml Eye drops Ocular 400

3307641000033112 3871711000001107 Livostin Direct 0.05% nasal spray (McNeil Products Ltd) Livostin Direct 0.05% nasal spray Levocabastine hydrochloride 500.000microgram/1.000ml Spray Nasal 90

1142341000033111 35933511000001100 Pseudoephedrine 30mg/5ml oral solution Pseudoephedrine 30mg/5ml oral solution Pseudoephedrine hydrochloride 6.000mg/1.000ml Oral solution Oral 3100000 599995

1142541000033116 3462411000001108 Pseudoephedrine 30mg/5ml oral solution sugar free Pseudoephedrine 30mg/5ml oral solution sugar free Pseudoephedrine hydrochloride 6.000mg/1.000ml Oral solution Oral 3100000 293888

1142941000033110 42195711000001104 Pseudoephedrine hydrochloride 60mg tablets Pseudoephedrine hydrochloride 60mg tablets Pseudoephedrine hydrochloride 60.000mg Tablet/ Oral Tablet Oral 3100000 956607

3246641000033113 3462311000001101 Pseudoephedrine 30mg/5ml / Triprolidine 1.25mg/5ml oral solution Pseudoephedrine 30mg/5ml / Triprolidine 1.25mg/5ml oral solution Pseudoephedrine hydrochloride/ Triprolidine hydrochloride 6.000mg/1.000ml + 250.000microgram/1.000ml Oral solution Oral 3100000 2355

3978241000033112 3462211000001109 Pseudoephedrine 30mg/5ml / Chlorphenamine 2mg/5ml oral solution sugar free Pseudoephedrine 30mg/5ml / Chlorphenamine 2mg/5ml oral solution sugar free Chlorphenamine maleate/ Pseudoephedrine hydrochloride 400.000microgram/1.000ml + 6.000mg/1.000ml Oral solution Oral 3100000 73138

4434541000033117 4018811000001101 Pholcodine 1.5mg/5ml / Promethazine 1.5mg/5ml oral solution sugar free Pholcodine 1.5mg/5ml / Promethazine 1.5mg/5ml oral solution sugar free Pholcodine/ Promethazine hydrochloride 300.000microgram/1.000ml + 300.000microgram/1.000ml Oral solution Oral 3090200 445

4561541000033115 42195611000001104 Pseudoephedrine hydrochloride 60mg / Triprolidine 2.5mg tablets Pseudoephedrine hydrochloride 60mg / Triprolidine 2.5mg tablets Pseudoephedrine hydrochloride/ Triprolidine hydrochloride 60.000mg + 2.500mg Tablet/ Oral Tablet Oral 3100000 5187

5008441000033115 35933411000001104 Pseudoephedrine 120mg modified-release tablets Pseudoephedrine 120mg modified-release tablets Pseudoephedrine hydrochloride 120.000mg Modified-release tablet Oral 3100000 2053

5263741000033114 4504111000001102 Diphenhydramine 10mg/5ml / Dextromethorphan 6.65mg/5ml oral solution sugar free Diphenhydramine 10mg/5ml / Dextromethorphan 6.65mg/5ml oral solution sugar free Dextromethorphan hydrobromide/ Diphenhydramine hydrochloride 1.330mg/1.000ml + 2.000mg/1.000ml Oral solution Oral 3090200 530

10267941000033110 875411000001108 Benadryl Allergy Relief Plus Decongestant capsules (McNeil Products Ltd) Benadryl Allergy Relief Plus Decongestant capsules Acrivastine/ Pseudoephedrine hydrochloride 8.000mg + 60.000mg Capsule/ Oral capsule Oral 5346

12910041000033116 3766111000001109 Ephedrine hydrochloride 15mg / Chlorphenamine 10mg tablets Ephedrine hydrochloride 15mg / Chlorphenamine 10mg tablets Chlorphenamine maleate/ Ephedrine hydrochloride 10.000mg + 15.000mg Tablet/ Oral Tablet Oral 3100000 363

13395041000033112 39690411000001104 Paracetamol 500mg / Diphenhydramine 25mg tablets Paracetamol 500mg / Diphenhydramine 25mg tablets Diphenhydramine hydrochloride/ Paracetamol 25.000mg + 500.000mg Tablet/ Oral Tablet Oral 4070100 64

13745641000033114 38957011000001104 Ketotifen 1mg/5ml oral solution sugar free (Special Order) Ketotifen 1mg/5ml oral solution sugar free Ketotifen fumarate 200.000microgram/1.000ml Oral solution 1

13745841000033110 39000311000001104 Ketotifen 1mg/5ml oral solution sugar free (Imported) Ketotifen 1mg/5ml oral solution sugar free Ketotifen fumarate 200.000microgram/1.000ml Oral solution 1

ProdCodeId DMDCode TermfromEMIS ProductName drugsubstancename substancestrength formulation routeofadministration bnfcode DrugIssues

21441000033117 4935611000001108 Afrazine 0.05% nasal spray (Schering-Plough Ltd) Afrazine 0.05% nasal spray Oxymetazoline hydrochloride 500.000microgram/1.000ml Spray Nasal 100

1024941000033110 42193611000001109 Oxymetazoline 0.05% nasal spray Oxymetazoline 0.05% nasal spray Oxymetazoline hydrochloride 500.000microgram/1.000ml Spray Nasal 12020200 2000

530141000033116 42188811000001102 Ephedrine 0.5% nasal drops Ephedrine 0.5% nasal drops Ephedrine hydrochloride 5.000mg/1.000ml Nasal drops Nasal 12020200 300000

530241000033111 42188911000001107 Ephedrine 1% nasal drops Ephedrine 1% nasal drops Ephedrine hydrochloride 10.000mg/1.000ml Nasal drops Nasal 12020200 90000

530341000033118 8793711000001100 Ephedrine 0.25% nasal drops Ephedrine 0.25% nasal drops Ephedrine hydrochloride 2.500mg/1.000ml Nasal drops Nasal 12020200 30000

6468041000033119 19698311000001103 Glucose 50% in glycerol nasal drops Glucose 50% in glycerol nasal drops Glucose 500.000mg/1.000ml Nasal drops Nasal 12020200 300

2983741000033114 5356411000001104 Tubilux 0.9% nasal drops (Tubilux Pharma Ltd) Tubilux 0.9% nasal drops Sodium chloride 9.000mg/1.000ml Nasal drops 1000

3314641000033115 9432811000001101 Sodium chloride 0.9% nasal drops (Orbis Consumer Products Ltd) Sodium chloride 0.9% nasal drops Sodium chloride 9.000mg/1.000ml Nasal drops 20000

3314741000033112 9433411000001107 Sodium chloride 0.9% nasal drops (RX Farma) Sodium chloride 0.9% nasal drops Sodium chloride 9.000mg/1.000ml Nasal drops 10000

3328941000033111 9500711000001106 Sodium chloride 0.9% nasal drops (Almus Pharmaceuticals Ltd) Sodium chloride 0.9% nasal drops Sodium chloride 9.000mg/1.000ml Nasal drops 5000

3339441000033113 9536811000001108 Sodium chloride 0.9% nasal drops (Actavis UK Ltd) Sodium chloride 0.9% nasal drops Sodium chloride 9.000mg/1.000ml Nasal drops 100

5439741000033116 35925311000001105 Sodium chloride 0.9% irrigation solution aerosol spray Sodium chloride 0.9% irrigation solution aerosol spray Sodium chloride 9.000mg/1.000ml Spray 60000

5439641000033113 7825011000001101 Stericlens sodium chloride 0.9% irrigation solution aerosol spray (Crest Medical Ltd) Stericlens sodium chloride 0.9% irrigation solution aerosol spray Sodium chloride 9.000mg/1.000ml Spray 50000

13030341000033114 36935511000001104 Respi-Clear 7% inhalation solution 4ml vials (Essential-Healthcare Ltd) Respi-Clear 7% inhalation solution 4ml vials Sodium chloride 70.000mg/1.000ml 800

13301241000033117 37756911000001107 Resp-Ease 3% inhalation solution 4ml ampoules (Venture Healthcare Ltd) Resp-Ease 3% inhalation solution 4ml ampoules Sodium chloride 30.000mg/1.000ml 100

13301341000033110 37757211000001101 Resp-Ease 6% inhalation solution 4ml ampoules (Venture Healthcare Ltd) Resp-Ease 6% inhalation solution 4ml ampoules Sodium chloride 60.000mg/1.000ml 50

12677241000033116 35874611000001106 PulmoClear 7% inhalation solution 4ml vials (TriOn Pharma Ltd) PulmoClear 7% inhalation solution 4ml vials Sodium chloride 70.000mg/1.000ml 2000

12677041000033112 35874211000001109 PulmoClear 3% inhalation solution 4ml vials (TriOn Pharma Ltd) PulmoClear 3% inhalation solution 4ml vials Sodium chloride 30.000mg/1.000ml 800

12660341000033116 35790111000001103 Salineb 0.9% inhalation solution 2.5ml vials (Sai-Meds Ltd) Salineb 0.9% inhalation solution 2.5ml vials Sodium chloride 9.000mg/1.000ml 3000

12512841000033115 35403611000001107 Sodium chloride 3% inhalation solution 4ml vials Sodium chloride 3% inhalation solution 4ml vials Sodium chloride 30.000mg/1.000ml 2000

12437841000033117 34917011000001101 Respi-Clear 0.9% inhalation solution 2.5ml vials (Essential-Healthcare Ltd) Respi-Clear 0.9% inhalation solution 2.5ml vials Sodium chloride 9.000mg/1.000ml 7000

12382841000033119 34713911000001100 Hydra-Neb 0.9% inhalation solution 2.5ml vials (Venture Healthcare Ltd) Hydra-Neb 0.9% inhalation solution 2.5ml vials Sodium chloride 9.000mg/1.000ml 1000

11414241000033116 32459311000001108 Resp-Ease 7% inhalation solution 4ml vials (Venture Healthcare Ltd) Resp-Ease 7% inhalation solution 4ml vials Sodium chloride 70.000mg/1.000ml 9000

10252241000033113 34713711000001102 Sodium chloride 0.9% inhalation solution 2.5ml vials Sodium chloride 0.9% inhalation solution 2.5ml vials Sodium chloride 9.000mg/1.000ml 20000

6522841000033113 19857311000001100 Mandanol 0.9% nasal drops (M & A Pharmachem Ltd) Mandanol 0.9% nasal drops Sodium chloride 9.000mg/1.000ml Nasal drops 800

6006641000033115 18100111000001100 MucoClear 3% inhalation solution 4ml ampoules (Pari Medical Ltd) MucoClear 3% inhalation solution 4ml ampoules Sodium chloride 30.000mg/1.000ml 20000

6006541000033116 18110811000001109 Sodium chloride 3% inhalation solution 4ml ampoules Sodium chloride 3% inhalation solution 4ml ampoules Sodium chloride 30.000mg/1.000ml 10000

5989341000033113 20595511000001105 Sodium chloride 7% inhalation solution 4ml vials Sodium chloride 7% inhalation solution 4ml vials Sodium chloride 70.000mg/1.000ml 60000

5820241000033111 20546711000001103 Nebusal 7% inhalation solution 4ml vials (Accord-UK Ltd) Nebusal 7% inhalation solution 4ml vials Sodium chloride 70.000mg/1.000ml 40000

5442141000033113 14063511000001108 Sodium chloride 6% inhalation solution 4ml ampoules Sodium chloride 6% inhalation solution 4ml ampoules Sodium chloride 60.000mg/1.000ml 10000

4597641000033114 14037711000001104 MucoClear 6% inhalation solution 4ml ampoules (Pari Medical Ltd) MucoClear 6% inhalation solution 4ml ampoules Sodium chloride 60.000mg/1.000ml 20000

13606041000033113 38681911000001107 PulmoClear 0.9% inhalation solution 2.5ml vials (TriOn Pharma Ltd) PulmoClear 0.9% inhalation solution 2.5ml vials Sodium chloride 9.000mg/1.000ml 2000

13909741000033116 39967111000001103 Bronchlear 3% inhalation solution 4ml vials (Essential-Healthcare Ltd) Bronchlear 3% inhalation solution 4ml vials Sodium chloride 30.000mg/1.000ml 20

14110041000033110 40999011000001108 Knoxzy 0.9% inhalation solution 2.5ml vials (Biovantic Pharma Ltd) Knoxzy 0.9% inhalation solution 2.5ml vials Sodium chloride 9.000mg/1.000ml 10

1382141000033113 34174811000001109 Saline 0.9% nebuliser liquid 2.5ml Steri-Neb unit dose ampoules (Teva UK Ltd) Saline 0.9% nebuliser liquid 2.5ml Steri-Neb unit dose ampoules Sodium chloride 9.000mg/1.000ml Nebuliser liquid Inhalation 100000

1751641000033115 34175011000001104 Saline Steripoules nebuliser liquid 2.5ml unit dose ampoules (Galen Ltd) Saline Steripoules nebuliser liquid 2.5ml unit dose ampoules Sodium chloride 9.000mg/1.000ml Nebuliser liquid Inhalation 200000

3929941000033112 20138811000001102 Sodium chloride 7% nebuliser liquid 5ml bottles Sodium chloride 7% nebuliser liquid 5ml bottles Sodium chloride 70.000mg/1.000ml Nebuliser liquid Inhalation 12020200 4000

4042841000033118 20137411000001103 Sodium chloride 5% nebuliser liquid 20ml vials Sodium chloride 5% nebuliser liquid 20ml vials Sodium chloride 50.000mg/1.000ml Nebuliser liquid Inhalation 100

1819241000033111 8394711000001102 Saline nebuliser liquid 20ml unit dose Steripoule vials (Galen Ltd) Saline nebuliser liquid 20ml unit dose Steripoule vials Sodium chloride 9.000mg/1.000ml Nebuliser liquid Inhalation 30000

5378441000033113 8967711000001102 Sodium chloride 7% nebuliser liquid Sodium chloride 7% nebuliser liquid Sodium chloride 70.000mg/1.000ml Nebuliser liquid Inhalation 12020200 2000

4088041000033117 17563811000001101 Sodium chloride 3% nebuliser liquid 20ml unit dose vials Sodium chloride 3% nebuliser liquid 20ml unit dose vials Sodium chloride 30.000mg/1.000ml Nebuliser liquid Inhalation 12020200 300

6066641000033116 17563611000001100 Sodium chloride 3% nebuliser liquid Sodium chloride 3% nebuliser liquid Sodium chloride 30.000mg/1.000ml Nebuliser liquid Inhalation 12020200 400

6066541000033117 17563711000001109 Sodium chloride 3% nebuliser liquid 10ml unit dose vials Sodium chloride 3% nebuliser liquid 10ml unit dose vials Sodium chloride 30.000mg/1.000ml Nebuliser liquid Inhalation 12020200 300

10252141000033118 22683811000001108 Sodium chloride 0.9% nebuliser liquid 2.5ml unit dose ampoules Sodium chloride 0.9% nebuliser liquid 2.5ml unit dose ampoules Sodium chloride 9.000mg/1.000ml Nebuliser liquid Inhalation 12020200 200000

1360341000033112 41748011000001106 Sodium chloride 0.9% nasal drops Sodium chloride 0.9% nasal drops Sodium chloride 9.000mg/1.000ml Nasal drops Nasal 2000000

1546041000033117 42197511000001100 Xylometazoline 0.1% nasal spray Xylometazoline 0.1% nasal spray Xylometazoline hydrochloride 1.000mg/1.000ml Spray Nasal 12020200 500000

1546141000033118 35368811000001107 Xylometazoline 0.05% nasal drops Xylometazoline 0.05% nasal drops Xylometazoline hydrochloride 500.000microgram/1.000ml Nasal drops Nasal 12020200 400000

1546241000033113 42197411000001104 Xylometazoline 0.1% nasal drops Xylometazoline 0.1% nasal drops Xylometazoline hydrochloride 1.000mg/1.000ml Nasal drops Nasal 12020200 200000

2969741000033114 729511000001106 Otradrops Adult Formula 0.1% nasal drops (Manx Healthcare Ltd) Otradrops Adult Formula 0.1% nasal drops Xylometazoline hydrochloride 1.000mg/1.000ml Nasal drops Nasal 30

2969841000033116 458311000001104 Otradrops Child nasal drops (Manx Healthcare Ltd) Otradrops Child nasal drops Xylometazoline hydrochloride 500.000microgram/1.000ml Nasal drops Nasal 50

2969941000033112 136011000001101 Otraspray nasal spray (Manx Healthcare Ltd) Otraspray nasal spray Xylometazoline hydrochloride 1.000mg/1.000ml Spray Nasal 50

3935041000033111 4935211000001106 Sudafed Blocked Nose 0.1% spray (McNeil Products Ltd) Sudafed Blocked Nose 0.1% spray Xylometazoline hydrochloride 1.000mg/1.000ml Spray Nasal 7000

12331241000033118 806311000001102 Otrivine Adult 0.1% nasal drops (Haleon UK Ltd) Otrivine Adult 0.1% nasal drops Xylometazoline hydrochloride 1.000mg/1.000ml Nasal drops Nasal 2000

12331341000033111 575711000001103 Otrivine Adult nasal spray (Haleon UK Ltd) Otrivine Adult nasal spray Xylometazoline hydrochloride 1.000mg/1.000ml Spray Nasal 6000

12331441000033117 355811000001106 Otrivine Child nasal drops (Haleon UK Ltd) Otrivine Child nasal drops Xylometazoline hydrochloride 500.000microgram/1.000ml Nasal drops Nasal 2000

14205641000033110 41565311000001104 Knoxzy 7% inhalation solution 4ml vials (Biovantic Pharma Ltd) Knoxzy 7% inhalation solution 4ml vials Sodium chloride 70.000mg/1.000ml 2

ProdCodeId DMDCode TermfromEMIS ProductName drugsubstancename substancestrength formulation routeofadministration bnfcode DrugIssues

2623141000033115 3112911000001102 Pulvinal Beclometasone Dipropionate 100micrograms/dose dry powder inhaler (Chiesi Ltd) Pulvinal Beclometasone Dipropionate 100micrograms/dose dry powder inhaler Beclometasone dipropionate 100.000microgram/1.000dose Inhalation powder Inhalation 20000

2623241000033110 3113411000001101 Pulvinal Beclometasone Dipropionate 200micrograms/dose dry powder inhaler (Chiesi Ltd) Pulvinal Beclometasone Dipropionate 200micrograms/dose dry powder inhaler Beclometasone dipropionate 200.000microgram/1.000dose Inhalation powder Inhalation 30000

2623341000033117 3175111000001103 Pulvinal Beclometasone Dipropionate 400micrograms/dose dry powder inhaler (Chiesi Ltd) Pulvinal Beclometasone Dipropionate 400micrograms/dose dry powder inhaler Beclometasone dipropionate 400.000microgram/1.000dose Inhalation powder Inhalation 10000

2725541000033116 3189711000001107 Beclometasone 100 Cyclocaps (Teva UK Ltd) Beclometasone 100 Cyclocaps Beclometasone dipropionate 100.000microgram Inhalation powder Inhalation 400

2725641000033115 3192611000001109 Beclometasone 200 Cyclocaps (Teva UK Ltd) Beclometasone 200 Cyclocaps Beclometasone dipropionate 200.000microgram Inhalation powder Inhalation 2000

2725741000033112 3194511000001100 Beclometasone 400 Cyclocaps (Teva UK Ltd) Beclometasone 400 Cyclocaps Beclometasone dipropionate 400.000microgram Inhalation powder Inhalation 1000

3080441000033114 35908811000001103 Beclometasone 250micrograms/dose inhaler Beclometasone 250micrograms/dose inhaler Beclometasone dipropionate 250.000microgram/1.000dose Pressurised inhalation Inhalation 3020000 3000000

3080541000033110 35908611000001102 Beclometasone 250micrograms/dose breath actuated inhaler Beclometasone 250micrograms/dose breath actuated inhaler Beclometasone dipropionate 250.000microgram/1.000dose Pressurised inhalation Inhalation 3020000 500000

3080641000033111 35909211000001109 Beclometasone 50micrograms/dose breath actuated inhaler Beclometasone 50micrograms/dose breath actuated inhaler Beclometasone dipropionate 50.000microgram/1.000dose Pressurised inhalation Inhalation 3020000 200000

3080741000033119 35907811000001102 Beclometasone 100micrograms/dose breath actuated inhaler Beclometasone 100micrograms/dose breath actuated inhaler Beclometasone dipropionate 100.000microgram/1.000dose Pressurised inhalation Inhalation 3020000 1000000

3081041000033114 35907711000001105 Beclometasone 100microgram inhalation powder blisters with device Beclometasone 100microgram inhalation powder blisters with device Beclometasone dipropionate 100.000microgram Inhalation powder Inhalation 3020000 20000

3081141000033113 35908311000001107 Beclometasone 200microgram inhalation powder blisters with device Beclometasone 200microgram inhalation powder blisters with device Beclometasone dipropionate 200.000microgram Inhalation powder Inhalation 3020000 70000

3081241000033118 35909011000001104 Beclometasone 400microgram inhalation powder blisters with device Beclometasone 400microgram inhalation powder blisters with device Beclometasone dipropionate 400.000microgram Inhalation powder Inhalation 3020000 30000

3081341000033111 35907611000001101 Beclometasone 100microgram inhalation powder blisters Beclometasone 100microgram inhalation powder blisters Beclometasone dipropionate 100.000microgram Inhalation powder Inhalation 3020000 50000

3081441000033117 35908211000001104 Beclometasone 200microgram inhalation powder blisters Beclometasone 200microgram inhalation powder blisters Beclometasone dipropionate 200.000microgram Inhalation powder Inhalation 3020000 200000

3081541000033116 35908911000001108 Beclometasone 400microgram inhalation powder blisters Beclometasone 400microgram inhalation powder blisters Beclometasone dipropionate 400.000microgram Inhalation powder Inhalation 3020000 200000

3081641000033115 35908511000001101 Beclometasone 200micrograms/dose inhaler Beclometasone 200micrograms/dose inhaler Beclometasone dipropionate 200.000microgram/1.000dose Pressurised inhalation Inhalation 3020000 3000000

3081841000033119 35909511000001107 Beclometasone 50micrograms/dose inhaler Beclometasone 50micrograms/dose inhaler Beclometasone dipropionate 50.000microgram/1.000dose Pressurised inhalation Inhalation 3020000 2000000

3081941000033110 39695711000001109 Beclometasone 400microgram inhalation powder capsules Beclometasone 400microgram inhalation powder capsules Beclometasone dipropionate 400.000microgram Inhalation powder Inhalation 3020000 70000

3082041000033116 39695511000001104 Beclometasone 100microgram inhalation powder capsules Beclometasone 100microgram inhalation powder capsules Beclometasone dipropionate 100.000microgram Inhalation powder Inhalation 3020000 30000

3082141000033117 39695611000001100 Beclometasone 200microgram inhalation powder capsules Beclometasone 200microgram inhalation powder capsules Beclometasone dipropionate 200.000microgram Inhalation powder Inhalation 3020000 90000

3082241000033112 35909411000001108 Beclometasone 50micrograms/dose dry powder inhaler Beclometasone 50micrograms/dose dry powder inhaler Beclometasone dipropionate 50.000microgram/1.000dose Inhalation powder Inhalation 3020000 6000

3082341000033119 35908011000001109 Beclometasone 100micrograms/dose dry powder inhaler Beclometasone 100micrograms/dose dry powder inhaler Beclometasone dipropionate 100.000microgram/1.000dose Inhalation powder Inhalation 3020000 60000

3082441000033113 35908711000001106 Beclometasone 250micrograms/dose dry powder inhaler Beclometasone 250micrograms/dose dry powder inhaler Beclometasone dipropionate 250.000microgram/1.000dose Inhalation powder Inhalation 3020000 30000

3082541000033114 35909111000001103 Beclometasone 400micrograms/dose dry powder inhaler Beclometasone 400micrograms/dose dry powder inhaler Beclometasone dipropionate 400.000microgram/1.000dose Inhalation powder Inhalation 3020000 20000

3082641000033110 35908411000001100 Beclometasone 200micrograms/dose dry powder inhaler Beclometasone 200micrograms/dose dry powder inhaler Beclometasone dipropionate 200.000microgram/1.000dose Inhalation powder Inhalation 3020000 90000

3082741000033118 35909611000001106 Beclometasone 50micrograms/dose inhaler CFC free Beclometasone 50micrograms/dose inhaler CFC free Beclometasone dipropionate 50.000microgram/1.000dose Pressurised inhalation Inhalation 3020000 300000

3082841000033111 35908111000001105 Beclometasone 100micrograms/dose inhaler CFC free Beclometasone 100micrograms/dose inhaler CFC free Beclometasone dipropionate 100.000microgram/1.000dose Pressurised inhalation Inhalation 3020000 800000

3082941000033115 35909311000001101 Beclometasone 50micrograms/dose breath actuated inhaler CFC free Beclometasone 50micrograms/dose breath actuated inhaler CFC free Beclometasone dipropionate 50.000microgram/1.000dose Pressurised inhalation Inhalation 3020000 50000

3083041000033113 35907911000001107 Beclometasone 100micrograms/dose breath actuated inhaler CFC free Beclometasone 100micrograms/dose breath actuated inhaler CFC free Beclometasone dipropionate 100.000microgram/1.000dose Pressurised inhalation Inhalation 3020000 300000

3085641000033118 39111911000001107 Beclometasone 50micrograms/dose nasal spray Beclometasone 50micrograms/dose nasal spray Beclometasone dipropionate 50.000microgram/1.000dose Spray Nasal 12020100 10000000

3343041000033119 9525111000001105 Easyhaler Beclometasone 200micrograms/dose dry powder inhaler (Orion Pharma (UK) Ltd) Easyhaler Beclometasone 200micrograms/dose dry powder inhaler Beclometasone dipropionate 200.000microgram/1.000dose Inhalation powder Inhalation 200000

3942841000033114 10621011000001101 Beclometasone 200micrograms/dose inhaler CFC free Beclometasone 200micrograms/dose inhaler CFC free Beclometasone dipropionate 200.000microgram/1.000dose Pressurised inhalation Inhalation 3020000 80000

3942941000033118 10621111000001100 Beclometasone 250micrograms/dose inhaler CFC free Beclometasone 250micrograms/dose inhaler CFC free Beclometasone dipropionate 250.000microgram/1.000dose Pressurised inhalation Inhalation 3020000 50000

4418141000033112 12911011000001100 Beclometasone 100micrograms/dose / Formoterol 6micrograms/dose inhaler CFC free Beclometasone 100micrograms/dose / Formoterol 6micrograms/dose inhaler CFC free Beclometasone dipropionate/ Formoterol fumarate dihydrate 100.000microgram/1.000dose + 6.000microgram/1.000dose Pressurised inhalation Inhalation 3020000 1000000

9531441000033113 26148711000001101 Beclometasone 100micrograms/dose / Formoterol 6micrograms/dose dry powder inhaler Beclometasone 100micrograms/dose / Formoterol 6micrograms/dose dry powder inhaler Beclometasone dipropionate/ Formoterol fumarate dihydrate 100.000microgram/1.000dose + 6.000microgram/1.000dose Inhalation powder Inhalation 3020000 100000

10735041000033115 31087511000001105 Beclometasone 200micrograms/dose / Formoterol 6micrograms/dose dry powder inhaler Beclometasone 200micrograms/dose / Formoterol 6micrograms/dose dry powder inhaler Beclometasone dipropionate/ Formoterol fumarate dihydrate 200.000microgram/1.000dose + 6.000microgram/1.000dose Inhalation powder Inhalation 3020000 60000

10740041000033111 31087411000001106 Beclometasone 200micrograms/dose / Formoterol 6micrograms/dose inhaler CFC free Beclometasone 200micrograms/dose / Formoterol 6micrograms/dose inhaler CFC free Beclometasone dipropionate/ Formoterol fumarate dihydrate 200.000microgram + 6.000microgram Pressurised inhalation Inhalation 3020000 90000

163741000033113 35912511000001101 Budesonide 200micrograms/dose inhaler Budesonide 200micrograms/dose inhaler Budesonide 200.000microgram/1.000dose Pressurised inhalation Inhalation 3020000 700000

163841000033115 35912811000001103 Budesonide 50micrograms/dose inhaler Budesonide 50micrograms/dose inhaler Budesonide 50.000microgram/1.000dose Pressurised inhalation Inhalation 3020000 200000

163941000033111 35912211000001104 Budesonide 100micrograms/dose nasal spray Budesonide 100micrograms/dose nasal spray Budesonide 100.000microgram/1.000dose Spray Nasal 12020100 400000

165241000033118 Budesonide Cream 0.025 % 200

169541000033119 Budesonide Nasal aerosol 50 micrograms/puff 10000

171241000033113 Budesonide Refill canister 200 micrograms/dose 50000

171341000033115 Budesonide Refill canister 50 micrograms/dose 10000

171641000033111 Budesonide Spacer inhaler 50 micrograms/dose 2000

171741000033119 Budesonide Spacer inhaler 200 micrograms/dose 1000

174541000033113 Budesonide Turbohaler 400 micrograms/dose 200

174641000033114 Budesonide Turbohaler 100 micrograms/dose 50

1579841000033116 Budesonide Breath-Actuated Dry Powder Inhaler 100 micrograms/dose 20

1579941000033112 Budesonide Breath-Actuated Dry Powder Inhaler 200 micrograms/dose 200

1915141000033110 3654511000001105 Budesonide 500micrograms/2ml nebuliser liquid unit dose vials Budesonide 500micrograms/2ml nebuliser liquid unit dose vials Budesonide 250.000microgram/1.000ml Nebuliser liquid Inhalation 3020000 100000

1915241000033115 3654611000001109 Budesonide 1mg/2ml nebuliser liquid unit dose vials Budesonide 1mg/2ml nebuliser liquid unit dose vials Budesonide 500.000microgram/1.000ml Nebuliser liquid Inhalation 3020000 100000

2725841000033119 3432911000001109 Budesonide 200microgram inhalation powder capsules Budesonide 200microgram inhalation powder capsules Budesonide 200.000microgram Inhalation powder Inhalation 3020000 1000

2725941000033110 3433011000001101 Budesonide 400microgram inhalation powder capsules Budesonide 400microgram inhalation powder capsules Budesonide 400.000microgram Inhalation powder Inhalation 3020000 200

2726041000033117 3197211000001105 Budesonide 200 Cyclocaps (Teva UK Ltd) Budesonide 200 Cyclocaps Budesonide 200.000microgram Inhalation powder Inhalation 10

2726141000033118 3198411000001108 Budesonide 400 Cyclocaps (Teva UK Ltd) Budesonide 400 Cyclocaps Budesonide 400.000microgram Inhalation powder Inhalation 8

2798441000033118 Budesonide Inhaler with spacer device 200 micrograms/dose 400

3075841000033110 7425611000001100 Budesonide 64micrograms/dose nasal spray Budesonide 64micrograms/dose nasal spray Budesonide 64.000microgram/1.000dose Spray Nasal 12020100 500000

3141341000033112 8024611000001102 Budesonide 200micrograms/dose dry powder inhalation cartridge with device Budesonide 200micrograms/dose dry powder inhalation cartridge with device Budesonide 200.000microgram/1.000dose Inhalation powder Inhalation 3020000 6000

3141441000033118 Novolizer Budesonide Inhalation Cartridge + Device 200 micrograms/dose, 100 doses 3000

3164341000033116 35912011000001109 Budesonide 100micrograms/dose / Formoterol 6micrograms/dose dry powder inhaler Budesonide 100micrograms/dose / Formoterol 6micrograms/dose dry powder inhaler Budesonide/ Formoterol fumarate dihydrate 100.000microgram/1.000dose + 6.000microgram/1.000dose Inhalation powder Inhalation 3020000 600000

3164441000033110 38896811000001103 Budesonide 200micrograms/dose / Formoterol 6micrograms/dose dry powder inhaler Budesonide 200micrograms/dose / Formoterol 6micrograms/dose dry powder inhaler Budesonide/ Formoterol fumarate dihydrate 200.000microgram/1.000dose + 6.000microgram/1.000dose Inhalation powder Inhalation 3020000 3000000

3164541000033111 4378111000001103 Budesonide 400micrograms/dose / Formoterol 12micrograms/dose dry powder inhaler Budesonide 400micrograms/dose / Formoterol 12micrograms/dose dry powder inhaler Budesonide/ Formoterol fumarate dihydrate 400.000microgram/1.000dose + 12.000microgram/1.000dose Inhalation powder Inhalation 3020000 2000000

3256341000033119 9117811000001107 Budesonide 200micrograms/dose dry powder inhalation cartridge Budesonide 200micrograms/dose dry powder inhalation cartridge Budesonide 200.000microgram/1.000dose Inhalation powder Inhalation 3020000 10000

3256441000033113 Novolizer Budesonide Inhalation Cartridge Refill 200 micrograms/dose, 100 doses 2000

3871541000033110 35912111000001105 Budesonide 100micrograms/dose dry powder inhaler Budesonide 100micrograms/dose dry powder inhaler Budesonide 100.000microgram/1.000dose Inhalation powder Inhalation 3020000 500000

3871641000033111 35912411000001100 Budesonide 200micrograms/dose dry powder inhaler Budesonide 200micrograms/dose dry powder inhaler Budesonide 200.000microgram/1.000dose Inhalation powder Inhalation 3020000 2000000

3871741000033119 35912711000001106 Budesonide 400micrograms/dose dry powder inhaler Budesonide 400micrograms/dose dry powder inhaler Budesonide 400.000microgram/1.000dose Inhalation powder Inhalation 3020000 900000

3871841000033112 10073911000001106 Easyhaler Budesonide 100micrograms/dose dry powder inhaler (Orion Pharma (UK) Ltd) Easyhaler Budesonide 100micrograms/dose dry powder inhaler Budesonide 100.000microgram/1.000dose Inhalation powder Inhalation 100000

3871941000033116 10074611000001102 Easyhaler Budesonide 200micrograms/dose dry powder inhaler (Orion Pharma (UK) Ltd) Easyhaler Budesonide 200micrograms/dose dry powder inhaler Budesonide 200.000microgram/1.000dose Inhalation powder Inhalation 60000

3872041000033110 10074411000001100 Easyhaler Budesonide 400micrograms/dose dry powder inhaler (Orion Pharma (UK) Ltd) Easyhaler Budesonide 400micrograms/dose dry powder inhaler Budesonide 400.000microgram/1.000dose Inhalation powder Inhalation 20000

4815041000033111 14959511000001107 Budesonide 100micrograms/dose inhaler CFC free Budesonide 100micrograms/dose inhaler CFC free Budesonide 100.000microgram/1.000dose Pressurised inhalation Inhalation 3020000 10000

4815141000033110 15374611000001106 Budesonide 200micrograms/dose inhaler CFC free Budesonide 200micrograms/dose inhaler CFC free Budesonide 200.000microgram/1.000dose Pressurised inhalation Inhalation 3020000 20000

11707241000033115 32960711000001105 Budesonide 200micrograms/dose / Formoterol 6micrograms/dose inhaler CFC free Budesonide 200micrograms/dose / Formoterol 6micrograms/dose inhaler CFC free Budesonide/ Formoterol fumarate dihydrate 200.000microgram/1.000dose + 6.000microgram/1.000dose Pressurised inhalation Inhalation 3020000 10000

13743741000033116 39133611000001108 Budesonide 100micrograms/dose / Formoterol 3micrograms/dose inhaler CFC free Budesonide 100micrograms/dose / Formoterol 3micrograms/dose inhaler CFC free Budesonide/ Formoterol fumarate dihydrate 100.000microgram/1.000dose + 3.000microgram/1.000dose Pressurised inhalation Inhalation 3020000 900

3227441000033112 9004511000001107 Ciclesonide 80micrograms/dose inhaler CFC free Ciclesonide 80micrograms/dose inhaler CFC free Ciclesonide 80.000microgram/1.000dose Pressurised inhalation Inhalation 3020000 40000

3227541000033113 Ciclesonide Cfc-free inhaler 160 micrograms/actuation, 120 doses 40000

3267641000033115 Ciclesonide Cfc-free inhaler 160 micrograms/actuation, 60 doses 10000

4823241000033113 9004411000001108 Ciclesonide 160micrograms/dose inhaler CFC free Ciclesonide 160micrograms/dose inhaler CFC free Ciclesonide 160.000microgram/1.000dose Pressurised inhalation Inhalation 3020000 90000

577141000033115 Fluticasone Propionate Accuhaler 100 micrograms/dose 10

577241000033110 Fluticasone Propionate Accuhaler 250 micrograms/dose 50

577441000033111 Fluticasone Propionate Accuhaler 500 micrograms/dose 90

577641000033113 Fluticasone Propionate Aqueous nasal spray 0.05 % (150 dose spray) 4000000

581641000033113 Fluticasone Propionate Disks with diskhaler 250 micrograms/dose 5

581741000033116 Fluticasone Propionate Disks with diskhaler 50 micrograms/dose 30

581941000033118 Fluticasone Propionate Disks with diskhaler 500 micrograms/puff 6

585941000033115 Fluticasone Propionate Inhaler 125 micrograms/puff 100000

586041000033113 36059111000001102 Fluticasone 25micrograms/dose inhaler Fluticasone 25micrograms/dose inhaler Fluticasone propionate 25.000microgram/1.000dose Pressurised inhalation Inhalation 3020000 60000

586141000033112 Fluticasone Propionate Inhaler 50 micrograms/puff 80000

586341000033110 Fluticasone Propionate Inhaler 250 micrograms/puff 400000

591141000033119 36059211000001108 Fluticasone propionate 100microgram inhalation powder blisters Fluticasone propionate 100microgram inhalation powder blisters Fluticasone propionate 100.000microgram Inhalation powder Inhalation 3020000 40000

591241000033114 36059511000001106 Fluticasone propionate 250microgram inhalation powder blisters Fluticasone propionate 250microgram inhalation powder blisters Fluticasone propionate 250.000microgram Inhalation powder Inhalation 3020000 90000

591341000033116 36059811000001109 Fluticasone propionate 50microgram inhalation powder blisters Fluticasone propionate 50microgram inhalation powder blisters Fluticasone propionate 50.000microgram Inhalation powder Inhalation 3020000 10000

591541000033111 36059611000001105 Fluticasone propionate 500microgram inhalation powder blisters Fluticasone propionate 500microgram inhalation powder blisters Fluticasone propionate 500.000microgram Inhalation powder Inhalation 3020000 20000

1730541000033114 39712511000001104 Fluticasone 2mg/2ml nebuliser liquid unit dose vials Fluticasone 2mg/2ml nebuliser liquid unit dose vials Fluticasone propionate 1.000mg/1.000ml Nebuliser liquid Inhalation 3020000 10000

1730641000033110 39712311000001105 Fluticasone 500micrograms/2ml nebuliser liquid unit dose vials Fluticasone 500micrograms/2ml nebuliser liquid unit dose vials Fluticasone propionate 250.000microgram/1.000ml Nebuliser liquid Inhalation 3020000 20000

1819841000033110 38894911000001100 Fluticasone 400microgram/unit dose nasal drops Fluticasone 400microgram/unit dose nasal drops Fluticasone propionate 400.000microgram Nasal drops Nasal 12020100 500000

1914341000033112 38897911000001106 Fluticasone propionate 50micrograms/dose dry powder inhaler Fluticasone propionate 50micrograms/dose dry powder inhaler Fluticasone propionate 50.000microgram/1.000dose Inhalation powder Inhalation 3020000 100000

1914441000033118 38897811000001101 Fluticasone propionate 100micrograms/dose dry powder inhaler Fluticasone propionate 100micrograms/dose dry powder inhaler Fluticasone propionate 100.000microgram/1.000dose Inhalation powder Inhalation 3020000 400000

1914541000033117 36565411000001101 Fluticasone propionate 250micrograms/dose dry powder inhaler Fluticasone propionate 250micrograms/dose dry powder inhaler Fluticasone propionate 250.000microgram/1.000dose Inhalation powder Inhalation 3020000 400000

1914641000033116 38897711000001109 Fluticasone propionate 500micrograms/dose dry powder inhaler Fluticasone propionate 500micrograms/dose dry powder inhaler Fluticasone propionate 500.000microgram/1.000dose Inhalation powder Inhalation 3020000 200000

1914741000033113 36059911000001104 Fluticasone propionate 50microgram inhalation powder blisters with device Fluticasone propionate 50microgram inhalation powder blisters with device Fluticasone propionate 50.000microgram Inhalation powder Inhalation 3020000 4000

1914841000033115 36059311000001100 Fluticasone propionate 100microgram inhalation powder blisters with device Fluticasone propionate 100microgram inhalation powder blisters with device Fluticasone propionate 100.000microgram Inhalation powder Inhalation 3020000 10000

1914941000033111 36059411000001107 Fluticasone propionate 250microgram inhalation powder blisters with device Fluticasone propionate 250microgram inhalation powder blisters with device Fluticasone propionate 250.000microgram Inhalation powder Inhalation 3020000 20000

1915041000033111 36059711000001101 Fluticasone propionate 500microgram inhalation powder blisters with device Fluticasone propionate 500microgram inhalation powder blisters with device Fluticasone propionate 500.000microgram Inhalation powder Inhalation 3020000 7000

2067741000033114 39111211000001103 Fluticasone 125micrograms/dose inhaler CFC free Fluticasone 125micrograms/dose inhaler CFC free Fluticasone propionate 125.000microgram/1.000dose Pressurised inhalation Inhalation 3020000 700000

2067841000033116 39111411000001104 Fluticasone 250micrograms/dose inhaler CFC free Fluticasone 250micrograms/dose inhaler CFC free Fluticasone propionate 250.000microgram/1.000dose Pressurised inhalation Inhalation 3020000 1000000

2148441000033115 39110911000001100 Fluticasone 50micrograms/dose inhaler CFC free Fluticasone 50micrograms/dose inhaler CFC free Fluticasone propionate 50.000microgram/1.000dose Pressurised inhalation Inhalation 3020000 700000

3163741000033118 39111011000001108 Fluticasone 50micrograms/dose / Salmeterol 25micrograms/dose inhaler CFC free Fluticasone 50micrograms/dose / Salmeterol 25micrograms/dose inhaler CFC free Fluticasone propionate/ Salmeterol xinafoate 50.000microgram/1.000dose + 25.000microgram/1.000dose Pressurised inhalation Inhalation 3020000 1000000

3163841000033111 39111111000001109 Fluticasone 125micrograms/dose / Salmeterol 25micrograms/dose inhaler CFC free Fluticasone 125micrograms/dose / Salmeterol 25micrograms/dose inhaler CFC free Fluticasone propionate/ Salmeterol xinafoate 125.000microgram/1.000dose + 25.000microgram/1.000dose Pressurised inhalation Inhalation 3020000 3000000

3163941000033115 39111311000001106 Fluticasone 250micrograms/dose / Salmeterol 25micrograms/dose inhaler CFC free Fluticasone 250micrograms/dose / Salmeterol 25micrograms/dose inhaler CFC free Fluticasone propionate/ Salmeterol xinafoate 250.000microgram/1.000dose + 25.000microgram/1.000dose Pressurised inhalation Inhalation 3020000 4000000

3164041000033118 38897611000001100 Fluticasone propionate 100micrograms/dose / Salmeterol 50micrograms/dose dry powder inhaler Fluticasone propionate 100micrograms/dose / Salmeterol 50micrograms/dose dry powder inhaler Fluticasone propionate/ Salmeterol xinafoate 100.000microgram/1.000dose + 50.000microgram/1.000dose Inhalation powder Inhalation 3020000 600000

3164141000033119 38897511000001104 Fluticasone propionate 250micrograms/dose / Salmeterol 50micrograms/dose dry powder inhaler Fluticasone propionate 250micrograms/dose / Salmeterol 50micrograms/dose dry powder inhaler Fluticasone propionate/ Salmeterol xinafoate 250.000microgram/1.000dose + 50.000microgram/1.000dose Inhalation powder Inhalation 3020000 1000000

3164241000033114 38897411000001103 Fluticasone propionate 500micrograms/dose / Salmeterol 50micrograms/dose dry powder inhaler Fluticasone propionate 500micrograms/dose / Salmeterol 50micrograms/dose dry powder inhaler Fluticasone propionate/ Salmeterol xinafoate 500.000microgram/1.000dose + 50.000microgram/1.000dose Inhalation powder Inhalation 3020000 2000000

4164541000033114 Fluticasone Propionate Aqueous nasal spray 50 micrograms/dose, 60 doses 20000

4435341000033113 39110511000001107 Fluticasone propionate 50micrograms/dose nasal spray Fluticasone propionate 50micrograms/dose nasal spray Fluticasone propionate 50.000microgram/1.000dose Spray Nasal 12020100 2000000

4892741000033116 42189811000001109 Fluticasone furoate 27.5micrograms/dose nasal spray Fluticasone furoate 27.5micrograms/dose nasal spray Fluticasone furoate 27.500microgram/1.000dose Spray Nasal 12020100 2000000

8101641000033112 21113911000001100 Fluticasone 50micrograms/dose / Formoterol 5micrograms/dose inhaler CFC free Fluticasone 50micrograms/dose / Formoterol 5micrograms/dose inhaler CFC free Fluticasone propionate/ Formoterol fumarate dihydrate 50.000microgram/1.000dose + 5.000microgram/1.000dose Pressurised inhalation Inhalation 3020000 50000

8101741000033115 21113711000001102 Fluticasone 125micrograms/dose / Formoterol 5micrograms/dose inhaler CFC free Fluticasone 125micrograms/dose / Formoterol 5micrograms/dose inhaler CFC free Fluticasone propionate/ Formoterol fumarate dihydrate 125.000microgram/1.000dose + 5.000microgram/1.000dose Pressurised inhalation Inhalation 3020000 200000

8101841000033113 21113811000001105 Fluticasone 250micrograms/dose / Formoterol 10micrograms/dose inhaler CFC free Fluticasone 250micrograms/dose / Formoterol 10micrograms/dose inhaler CFC free Fluticasone propionate/ Formoterol fumarate dihydrate 250.000microgram/1.000dose + 10.000microgram/1.000dose Pressurised inhalation Inhalation 3020000 200000

8265741000033116 21994411000001108 Fluticasone propionate 50micrograms/dose / Azelastine 137micrograms/dose nasal spray Fluticasone propionate 50micrograms/dose / Azelastine 137micrograms/dose nasal spray Azelastine hydrochloride/ Fluticasone propionate 137.000microgram/1.000dose + 50.000microgram/1.000dose Spray Nasal 12020100 300000

8946841000033112 23661311000001105 Fluticasone furoate 184micrograms/dose / Vilanterol 22micrograms/dose dry powder inhaler Fluticasone furoate 184micrograms/dose / Vilanterol 22micrograms/dose dry powder inhaler Fluticasone furoate/ Vilanterol 184.000microgram/1.000dose + 22.000microgram/1.000dose Inhalation powder Inhalation 3020000 50000

8946941000033116 23661411000001103 Fluticasone furoate 92micrograms/dose / Vilanterol 22micrograms/dose dry powder inhaler Fluticasone furoate 92micrograms/dose / Vilanterol 22micrograms/dose dry powder inhaler Fluticasone furoate/ Vilanterol 92.000microgram/1.000dose + 22.000microgram/1.000dose Inhalation powder Inhalation 3020000 200000

12634941000033116 35647511000001107 Fluticasone 125micrograms/dose / Formoterol 5micrograms/dose breath actuated inhaler CFC free Fluticasone 125micrograms/dose / Formoterol 5micrograms/dose breath actuated inhaler CFC free Fluticasone propionate/ Formoterol fumarate dihydrate 125.000microgram/1.000dose + 5.000microgram/1.000dose Pressurised inhalation Inhalation 3020000 3000

12635041000033116 35647611000001106 Fluticasone 50micrograms/dose / Formoterol 5micrograms/dose breath actuated inhaler CFC free Fluticasone 50micrograms/dose / Formoterol 5micrograms/dose breath actuated inhaler CFC free Fluticasone propionate/ Formoterol fumarate dihydrate 50.000microgram/1.000dose + 5.000microgram/1.000dose Pressurised inhalation Inhalation 3020000 2000

13740941000033111 38960111000001101 Fluticasone 500micrograms/2ml nebuliser liquid unit dose vials (Imported) Fluticasone 500micrograms/2ml nebuliser liquid unit dose vials Fluticasone propionate 250.000microgram/1.000ml Nebuliser liquid 2

13980041000033111 40455711000001101 Fluticasone propionate 100micrograms/dose / Salmeterol 12.75micrograms/dose dry powder inhaler Fluticasone propionate 100micrograms/dose / Salmeterol 12.75micrograms/dose dry powder inhaler Fluticasone propionate/ Salmeterol xinafoate 100.000microgram/1.000dose + 12.750microgram/1.000dose Inhalation powder Inhalation 3020000 6

589041000033118 42189611000001105 Flunisolide 25micrograms/dose nasal spray Flunisolide 25micrograms/dose nasal spray Flunisolide 25.000microgram/1.000dose Spray Nasal 12020100 200000

576741000033118 3184311000001107 Flixotide 100micrograms/dose Accuhaler (GlaxoSmithKline UK Ltd) Flixotide 100micrograms/dose Accuhaler Fluticasone propionate 100.000microgram/1.000dose Inhalation powder Inhalation 200000

576841000033111 3184911000001108 Flixotide 250micrograms/dose Accuhaler (GlaxoSmithKline UK Ltd) Flixotide 250micrograms/dose Accuhaler Fluticasone propionate 250.000microgram/1.000dose Inhalation powder Inhalation 200000

576941000033115 3183811000001101 Flixotide 50micrograms/dose Accuhaler (GlaxoSmithKline UK Ltd) Flixotide 50micrograms/dose Accuhaler Fluticasone propionate 50.000microgram/1.000dose Inhalation powder Inhalation 70000

577041000033119 3185211000001103 Flixotide 500micrograms/dose Accuhaler (GlaxoSmithKline UK Ltd) Flixotide 500micrograms/dose Accuhaler Fluticasone propionate 500.000microgram/1.000dose Inhalation powder Inhalation 90000

577541000033112 480211000001100 Flixonase 50micrograms/dose aqueous nasal spray (GlaxoSmithKline UK Ltd) Flixonase 50micrograms/dose aqueous nasal spray Fluticasone propionate 50.000microgram/1.000dose Spray Nasal 3000000

581241000033110 3098611000001101 Flixotide 100microgram disks with Diskhaler (GlaxoSmithKline UK Ltd) Flixotide 100microgram disks with Diskhaler Fluticasone propionate 100.000microgram Inhalation powder Inhalation 20000

581341000033117 3106311000001102 Flixotide 250microgram disks with Diskhaler (GlaxoSmithKline UK Ltd) Flixotide 250microgram disks with Diskhaler Fluticasone propionate 250.000microgram Inhalation powder Inhalation 40000

581441000033111 3097711000001109 Flixotide 50microgram disks with Diskhaler (GlaxoSmithKline UK Ltd) Flixotide 50microgram disks with Diskhaler Fluticasone propionate 50.000microgram Inhalation powder Inhalation 10000

581841000033114 3108411000001105 Flixotide 500microgram disks with Diskhaler (GlaxoSmithKline UK Ltd) Flixotide 500microgram disks with Diskhaler Fluticasone propionate 500.000microgram Inhalation powder Inhalation 8000

585741000033118 4856011000001103 Flixotide 25micrograms/dose inhaler (GlaxoSmithKline UK Ltd) Flixotide 25micrograms/dose inhaler Fluticasone propionate 25.000microgram/1.000dose Pressurised inhalation Inhalation 30000

590841000033115 3099611000001105 Flixotide 100microgram disks (GlaxoSmithKline UK Ltd) Flixotide 100microgram disks Fluticasone propionate 100.000microgram Inhalation powder Inhalation 30000

590941000033111 3103511000001100 Flixotide 250microgram disks (GlaxoSmithKline UK Ltd) Flixotide 250microgram disks Fluticasone propionate 250.000microgram Inhalation powder Inhalation 70000

591041000033118 3100811000001109 Flixotide 50microgram disks (GlaxoSmithKline UK Ltd) Flixotide 50microgram disks Fluticasone propionate 50.000microgram Inhalation powder Inhalation 10000

591441000033110 3110511000001108 Flixotide 500microgram disks (GlaxoSmithKline UK Ltd) Flixotide 500microgram disks Fluticasone propionate 500.000microgram Inhalation powder Inhalation 20000

1730341000033119 3397211000001103 Flixotide 2mg/2ml Nebules (GlaxoSmithKline UK Ltd) Flixotide 2mg/2ml Nebules Fluticasone propionate 1.000mg/1.000ml Nebuliser liquid Inhalation 7000

1730441000033113 3389111000001102 Flixotide 0.5mg/2ml Nebules (GlaxoSmithKline UK Ltd) Flixotide 0.5mg/2ml Nebules Fluticasone propionate 250.000microgram/1.000ml Nebuliser liquid Inhalation 7000

1752141000033117 3186911000001100 Seretide 100 Accuhaler (GlaxoSmithKline UK Ltd) Seretide 100 Accuhaler Fluticasone propionate/ Salmeterol xinafoate 100.000microgram/1.000dose + 50.000microgram/1.000dose Inhalation powder Inhalation 1000000

1752241000033112 3187211000001106 Seretide 250 Accuhaler (GlaxoSmithKline UK Ltd) Seretide 250 Accuhaler Fluticasone propionate/ Salmeterol xinafoate 250.000microgram/1.000dose + 50.000microgram/1.000dose Inhalation powder Inhalation 2000000

1752341000033119 3188311000001102 Seretide 500 Accuhaler (GlaxoSmithKline UK Ltd) Seretide 500 Accuhaler Fluticasone propionate/ Salmeterol xinafoate 500.000microgram/1.000dose + 50.000microgram/1.000dose Inhalation powder Inhalation 4000000

1820041000033116 4380511000001101 Flixonase Nasule 400microgram/unit dose nasal drops (GlaxoSmithKline UK Ltd) Flixonase Nasule 400microgram/unit dose nasal drops Fluticasone propionate 400.000microgram Nasal drops Nasal 400000

2067541000033118 398511000001105 Flixotide 125micrograms/dose Evohaler (GlaxoSmithKline UK Ltd) Flixotide 125micrograms/dose Evohaler Fluticasone propionate 125.000microgram/1.000dose Pressurised inhalation Inhalation 200000

2067641000033117 2831211000001109 Flixotide 250micrograms/dose Evohaler (GlaxoSmithKline UK Ltd) Flixotide 250micrograms/dose Evohaler Fluticasone propionate 250.000microgram/1.000dose Pressurised inhalation Inhalation 300000

2147241000033117 453611000001102 Seretide 50 Evohaler (GlaxoSmithKline UK Ltd) Seretide 50 Evohaler Fluticasone propionate/ Salmeterol xinafoate 50.000microgram/1.000dose + 25.000microgram/1.000dose Pressurised inhalation Inhalation 2000000

2147341000033110 810211000001105 Seretide 125 Evohaler (GlaxoSmithKline UK Ltd) Seretide 125 Evohaler Fluticasone propionate/ Salmeterol xinafoate 125.000microgram/1.000dose + 25.000microgram/1.000dose Pressurised inhalation Inhalation 4000000

2147441000033116 539811000001106 Seretide 250 Evohaler (GlaxoSmithKline UK Ltd) Seretide 250 Evohaler Fluticasone propionate/ Salmeterol xinafoate 250.000microgram/1.000dose + 25.000microgram/1.000dose Pressurised inhalation Inhalation 4000000

2148541000033119 726611000001102 Flixotide 50micrograms/dose Evohaler (GlaxoSmithKline UK Ltd) Flixotide 50micrograms/dose Evohaler Fluticasone propionate 50.000microgram/1.000dose Pressurised inhalation Inhalation 200000

3290841000033117 9310711000001107 Nasofan 50micrograms/dose aqueous nasal spray (Teva UK Ltd) Nasofan 50micrograms/dose aqueous nasal spray Fluticasone propionate 50.000microgram/1.000dose Spray Nasal 400000

4164641000033110 11177911000001100 Nasofan Allergy 50micrograms/dose nasal spray (Teva UK Ltd) Nasofan Allergy 50micrograms/dose nasal spray Fluticasone propionate 50.000microgram/1.000dose Spray Nasal 20000

4892841000033114 15046311000001109 Avamys 27.5micrograms/dose nasal spray (GlaxoSmithKline UK Ltd) Avamys 27.5micrograms/dose nasal spray Fluticasone furoate 27.500microgram/1.000dose Spray Nasal 3000000

6136441000033119 4365311000001103 Pirinase Hayfever 0.05% nasal spray (Haleon UK Ltd) Pirinase Hayfever 0.05% nasal spray Fluticasone propionate 50.000microgram/1.000dose Spray Nasal 10000

8101941000033117 21020611000001104 Flutiform 50micrograms/dose / 5micrograms/dose inhaler (Napp Pharmaceuticals Ltd) Flutiform 50micrograms/dose / 5micrograms/dose inhaler Fluticasone propionate/ Formoterol fumarate dihydrate 50.000microgram/1.000dose + 5.000microgram/1.000dose Pressurised inhalation Inhalation 200000

8102041000033111 21019411000001101 Flutiform 125micrograms/dose / 5micrograms/dose inhaler (Napp Pharmaceuticals Ltd) Flutiform 125micrograms/dose / 5micrograms/dose inhaler Fluticasone propionate/ Formoterol fumarate dihydrate 125.000microgram/1.000dose + 5.000microgram/1.000dose Pressurised inhalation Inhalation 600000

8102141000033110 21019711000001107 Flutiform 250micrograms/dose / 10micrograms/dose inhaler (Napp Pharmaceuticals Ltd) Flutiform 250micrograms/dose / 10micrograms/dose inhaler Fluticasone propionate/ Formoterol fumarate dihydrate 250.000microgram/1.000dose + 10.000microgram/1.000dose Pressurised inhalation Inhalation 500000

8265841000033114 21976811000001105 Dymista 137micrograms/dose / 50micrograms/dose nasal spray (Viatris UK Healthcare Ltd) Dymista 137micrograms/dose / 50micrograms/dose nasal spray Azelastine hydrochloride/ Fluticasone propionate 137.000microgram/1.000dose + 50.000microgram/1.000dose Spray Nasal 500000

8947041000033115 23621711000001102 Relvar Ellipta 184micrograms/dose / 22micrograms/dose dry powder inhaler (GlaxoSmithKline UK Ltd) Relvar Ellipta 184micrograms/dose / 22micrograms/dose dry powder inhaler Fluticasone furoate/ Vilanterol 184.000microgram/1.000dose + 22.000microgram/1.000dose Inhalation powder Inhalation 500000

8947141000033116 23622011000001107 Relvar Ellipta 92micrograms/dose / 22micrograms/dose dry powder inhaler (GlaxoSmithKline UK Ltd) Relvar Ellipta 92micrograms/dose / 22micrograms/dose dry powder inhaler Fluticasone furoate/ Vilanterol 92.000microgram/1.000dose + 22.000microgram/1.000dose Inhalation powder Inhalation 1000000

10387141000033115 29782111000001107 Sirdupla 25micrograms/dose / 125micrograms/dose inhaler (Viatris UK Healthcare Ltd) Sirdupla 25micrograms/dose / 125micrograms/dose inhaler Fluticasone propionate/ Salmeterol xinafoate 125.000microgram/1.000dose + 25.000microgram/1.000dose Pressurised inhalation Inhalation 500000

10387241000033110 29782511000001103 Sirdupla 25micrograms/dose / 250micrograms/dose inhaler (Viatris UK Healthcare Ltd) Sirdupla 25micrograms/dose / 250micrograms/dose inhaler Fluticasone propionate/ Salmeterol xinafoate 250.000microgram/1.000dose + 25.000microgram/1.000dose Pressurised inhalation Inhalation 600000

10715041000033111 30950311000001106 AirFluSal Forspiro 50micrograms/dose / 500micrograms/dose dry powder inhaler (Sandoz Ltd) AirFluSal Forspiro 50micrograms/dose / 500micrograms/dose dry powder inhaler Fluticasone propionate/ Salmeterol xinafoate 500.000microgram/1.000dose + 50.000microgram/1.000dose Inhalation powder Inhalation 100000

11026941000033116 31572011000001107 Pirinase Hayfever Relief for Adults 0.05% nasal spray (Haleon UK Ltd) Pirinase Hayfever Relief for Adults 0.05% nasal spray Fluticasone propionate 50.000microgram/1.000dose Spray Nasal 800

11899741000033117 33679711000001103 Aerivio Spiromax 50micrograms/dose / 500micrograms/dose dry powder inhaler (Teva UK Ltd) Aerivio Spiromax 50micrograms/dose / 500micrograms/dose dry powder inhaler Fluticasone propionate/ Salmeterol xinafoate 500.000microgram/1.000dose + 50.000microgram/1.000dose Inhalation powder Inhalation 10000

12046641000033114 34023611000001101 Sereflo 25micrograms/dose / 125micrograms/dose inhaler (Cipla EU Ltd) Sereflo 25micrograms/dose / 125micrograms/dose inhaler Fluticasone propionate/ Salmeterol xinafoate 125.000microgram/1.000dose + 25.000microgram/1.000dose Pressurised inhalation Inhalation 200000

12046741000033117 34023811000001102 Sereflo 25micrograms/dose / 250micrograms/dose inhaler (Cipla EU Ltd) Sereflo 25micrograms/dose / 250micrograms/dose inhaler Fluticasone propionate/ Salmeterol xinafoate 250.000microgram/1.000dose + 25.000microgram/1.000dose Pressurised inhalation Inhalation 200000

12197841000033113 34215311000001107 AirFluSal 25micrograms/dose / 125micrograms/dose inhaler (Sandoz Ltd) AirFluSal 25micrograms/dose / 125micrograms/dose inhaler Fluticasone propionate/ Salmeterol xinafoate 125.000microgram/1.000dose + 25.000microgram/1.000dose Pressurised inhalation Inhalation 100000

12197941000033117 34215511000001101 AirFluSal 25micrograms/dose / 250micrograms/dose inhaler (Sandoz Ltd) AirFluSal 25micrograms/dose / 250micrograms/dose inhaler Fluticasone propionate/ Salmeterol xinafoate 250.000microgram/1.000dose + 25.000microgram/1.000dose Pressurised inhalation Inhalation 90000

12370841000033110 34677011000001107 Aloflute 25micrograms/dose / 125micrograms/dose inhaler (Viatris UK Healthcare Ltd) Aloflute 25micrograms/dose / 125micrograms/dose inhaler Fluticasone propionate/ Salmeterol xinafoate 125.000microgram/1.000dose + 25.000microgram/1.000dose Pressurised inhalation Inhalation 4000

12370941000033119 34675711000001103 Aloflute 25micrograms/dose / 250micrograms/dose inhaler (Viatris UK Healthcare Ltd) Aloflute 25micrograms/dose / 250micrograms/dose inhaler Fluticasone propionate/ Salmeterol xinafoate 250.000microgram/1.000dose + 25.000microgram/1.000dose Pressurised inhalation Inhalation 5000

12431141000033117 34952211000001104 Trelegy Ellipta 92micrograms/dose / 55micrograms/dose / 22micrograms/dose dry powder inhaler (GlaxoSmithKline UK Ltd) Trelegy Ellipta 92micrograms/dose / 55micrograms/dose / 22micrograms/dose dry powder inhaler Fluticasone furoate/ Umeclidinium bromide/ Vilanterol trifenatate 92.000microgram/1.000dose + 55.000microgram/1.000dose + 22.000microgram/1.000dose Inhalation powder Inhalation 1000000

12529241000033119 35515511000001100 Fusacomb Easyhaler 50micrograms/dose / 250micrograms/dose dry powder inhaler (Orion Pharma (UK) Ltd) Fusacomb Easyhaler 50micrograms/dose / 250micrograms/dose dry powder inhaler Fluticasone propionate/ Salmeterol xinafoate 250.000microgram/1.000dose + 50.000microgram/1.000dose Inhalation powder Inhalation 5000

12529341000033112 35515311000001106 Fusacomb Easyhaler 50micrograms/dose / 500micrograms/dose dry powder inhaler (Orion Pharma (UK) Ltd) Fusacomb Easyhaler 50micrograms/dose / 500micrograms/dose dry powder inhaler Fluticasone propionate/ Salmeterol xinafoate 500.000microgram/1.000dose + 50.000microgram/1.000dose Inhalation powder Inhalation 3000

12579341000033113 35594011000001105 Combisal 25micrograms/dose / 50micrograms/dose inhaler (Aspire Pharma Ltd) Combisal 25micrograms/dose / 50micrograms/dose inhaler Fluticasone propionate/ Salmeterol xinafoate 50.000microgram/1.000dose + 25.000microgram/1.000dose Pressurised inhalation Inhalation 40000

12579441000033119 35594211000001100 Combisal 25micrograms/dose / 125micrograms/dose inhaler (Aspire Pharma Ltd) Combisal 25micrograms/dose / 125micrograms/dose inhaler Fluticasone propionate/ Salmeterol xinafoate 125.000microgram/1.000dose + 25.000microgram/1.000dose Pressurised inhalation Inhalation 40000

12579541000033118 35594411000001101 Combisal 25micrograms/dose / 250micrograms/dose inhaler (Aspire Pharma Ltd) Combisal 25micrograms/dose / 250micrograms/dose inhaler Fluticasone propionate/ Salmeterol xinafoate 250.000microgram/1.000dose + 25.000microgram/1.000dose Pressurised inhalation Inhalation 40000

12635141000033117 35647311000001101 Flutiform K-haler 125micrograms/dose / 5micrograms/dose breath actuated inhaler (Napp Pharmaceuticals Ltd) Flutiform K-haler 125micrograms/dose / 5micrograms/dose breath actuated inhaler Fluticasone propionate/ Formoterol fumarate dihydrate 125.000microgram/1.000dose + 5.000microgram/1.000dose Pressurised inhalation Inhalation 4000

12635241000033112 35650811000001109 Flutiform K-haler 50micrograms/dose / 5micrograms/dose breath actuated inhaler (Napp Pharmaceuticals Ltd) Flutiform K-haler 50micrograms/dose / 5micrograms/dose breath actuated inhaler Fluticasone propionate/ Formoterol fumarate dihydrate 50.000microgram/1.000dose + 5.000microgram/1.000dose Pressurised inhalation Inhalation 2000

12901741000033113 36604711000001102 Stalpex 50micrograms/dose / 500micrograms/dose dry powder inhaler (Glenmark Pharmaceuticals Europe Ltd) Stalpex 50micrograms/dose / 500micrograms/dose dry powder inhaler Fluticasone propionate/ Salmeterol xinafoate 500.000microgram/1.000dose + 50.000microgram/1.000dose Inhalation powder Inhalation 9000

13815341000033114 39567811000001100 Fixkoh Airmaster 50micrograms/dose / 100micrograms/dose dry powder inhaler (Genus Pharmaceuticals Ltd) Fixkoh Airmaster 50micrograms/dose / 100micrograms/dose dry powder inhaler Fluticasone propionate/ Salmeterol xinafoate 100.000microgram/1.000dose + 50.000microgram/1.000dose Inhalation powder Inhalation 200

13815441000033115 39567611000001104 Fixkoh Airmaster 50micrograms/dose / 250micrograms/dose dry powder inhaler (Genus Pharmaceuticals Ltd) Fixkoh Airmaster 50micrograms/dose / 250micrograms/dose dry powder inhaler Fluticasone propionate/ Salmeterol xinafoate 250.000microgram/1.000dose + 50.000microgram/1.000dose Inhalation powder Inhalation 200

13815541000033119 39567411000001102 Fixkoh Airmaster 50micrograms/dose / 500micrograms/dose dry powder inhaler (Genus Pharmaceuticals Ltd) Fixkoh Airmaster 50micrograms/dose / 500micrograms/dose dry powder inhaler Fluticasone propionate/ Salmeterol xinafoate 500.000microgram/1.000dose + 50.000microgram/1.000dose Inhalation powder Inhalation 200

13933441000033119 40040711000001101 Avenor 25micrograms/dose / 125micrograms/dose inhaler (Zentiva Pharma UK Ltd) Avenor 25micrograms/dose / 125micrograms/dose inhaler Fluticasone propionate/ Salmeterol xinafoate 125.000microgram/1.000dose + 25.000microgram/1.000dose Pressurised inhalation Inhalation 200

13933541000033118 40040911000001104 Avenor 25micrograms/dose / 250micrograms/dose inhaler (Zentiva Pharma UK Ltd) Avenor 25micrograms/dose / 250micrograms/dose inhaler Fluticasone propionate/ Salmeterol xinafoate 250.000microgram/1.000dose + 25.000microgram/1.000dose Pressurised inhalation Inhalation 60

13933641000033117 40034211000001104 Avenor 25micrograms/dose / 50micrograms/dose inhaler (Zentiva Pharma UK Ltd) Avenor 25micrograms/dose / 50micrograms/dose inhaler Fluticasone propionate/ Salmeterol xinafoate 50.000microgram/1.000dose + 25.000microgram/1.000dose Pressurised inhalation Inhalation 500

13980441000033119 40445111000001100 Seffalair Spiromax 12.75micrograms/dose / 100micrograms/dose dry powder inhaler (Teva UK Ltd) Seffalair Spiromax 12.75micrograms/dose / 100micrograms/dose dry powder inhaler Fluticasone propionate/ Salmeterol xinafoate 100.000microgram/1.000dose + 12.750microgram/1.000dose Inhalation powder Inhalation 6

13980541000033118 40444911000001101 Seffalair Spiromax 12.75micrograms/dose / 202micrograms/dose dry powder inhaler (Teva UK Ltd) Seffalair Spiromax 12.75micrograms/dose / 202micrograms/dose dry powder inhaler Fluticasone propionate/ Salmeterol xinafoate 202.000microgram/1.000dose + 12.750microgram/1.000dose Inhalation powder Inhalation 20

14005841000033118 40504911000001103 Sereflo Ciphaler 50micrograms/dose / 250micrograms/dose dry powder inhaler (Cipla EU Ltd) Sereflo Ciphaler 50micrograms/dose / 250micrograms/dose dry powder inhaler Fluticasone propionate/ Salmeterol xinafoate 250.000microgram/1.000dose + 50.000microgram/1.000dose Inhalation powder Inhalation 100

14116741000033110 41158211000001107 Campona Airmaster 50micrograms/dose / 100micrograms/dose dry powder inhaler (Genesis Pharmaceuticals Ltd) Campona Airmaster 50micrograms/dose / 100micrograms/dose dry powder inhaler Fluticasone propionate/ Salmeterol xinafoate 100.000microgram/1.000dose + 50.000microgram/1.000dose Inhalation powder Inhalation 3

14116941000033113 41158811000001108 Campona Airmaster 50micrograms/dose / 250micrograms/dose dry powder inhaler (Genesis Pharmaceuticals Ltd) Campona Airmaster 50micrograms/dose / 250micrograms/dose dry powder inhaler Fluticasone propionate/ Salmeterol xinafoate 250.000microgram/1.000dose + 50.000microgram/1.000dose Inhalation powder Inhalation 8

14117041000033114 41159011000001107 Campona Airmaster 50micrograms/dose / 500micrograms/dose dry powder inhaler (Genesis Pharmaceuticals Ltd) Campona Airmaster 50micrograms/dose / 500micrograms/dose dry powder inhaler Fluticasone propionate/ Salmeterol xinafoate 500.000microgram/1.000dose + 50.000microgram/1.000dose Inhalation powder Inhalation 2

14192141000033111 41619211000001108 Sereflo Ciphaler 50micrograms/dose / 500micrograms/dose dry powder inhaler (Cipla EU Ltd) Sereflo Ciphaler 50micrograms/dose / 500micrograms/dose dry powder inhaler Fluticasone propionate/ Salmeterol xinafoate 500.000microgram/1.000dose + 50.000microgram/1.000dose Inhalation powder Inhalation 10

3227641000033114 9003911000001102 Alvesco 80 inhaler (Zentiva Pharma UK Ltd) Alvesco 80 inhaler Ciclesonide 80.000microgram/1.000dose Pressurised inhalation Inhalation 10000

4823341000033115 9004211000001109 Alvesco 160 inhaler (Zentiva Pharma UK Ltd) Alvesco 160 inhaler Ciclesonide 160.000microgram/1.000dose Pressurised inhalation Inhalation 30000

20141000033118 3604911000001105 AeroBec 50 Autohaler (Meda Pharmaceuticals Ltd) AeroBec 50 Autohaler Beclometasone dipropionate 50.000microgram/1.000dose Pressurised inhalation Inhalation 20000

20241000033113 3182411000001107 AeroBec Forte 250 Autohaler (Meda Pharmaceuticals Ltd) AeroBec Forte 250 Autohaler Beclometasone dipropionate 250.000microgram/1.000dose Pressurised inhalation Inhalation 40000

20341000033115 3181411000001102 AeroBec 100 Autohaler (Meda Pharmaceuticals Ltd) AeroBec 100 Autohaler Beclometasone dipropionate 100.000microgram/1.000dose Pressurised inhalation Inhalation 200000

121641000033112 3180211000001107 Becloforte 250micrograms/dose inhaler (GlaxoSmithKline UK Ltd) Becloforte 250micrograms/dose inhaler Beclometasone dipropionate 250.000microgram/1.000dose Pressurised inhalation Inhalation 1000000

123241000033110 3181711000001108 Beclazone 100 Easi-Breathe inhaler (Teva UK Ltd) Beclazone 100 Easi-Breathe inhaler Beclometasone dipropionate 100.000microgram/1.000dose Pressurised inhalation Inhalation 500000

123341000033117 3604611000001104 Beclazone 50 Easi-Breathe inhaler (Teva UK Ltd) Beclazone 50 Easi-Breathe inhaler Beclometasone dipropionate 50.000microgram/1.000dose Pressurised inhalation Inhalation 50000

123441000033111 3182611000001105 Beclazone 250 Easi-Breathe inhaler (Teva UK Ltd) Beclazone 250 Easi-Breathe inhaler Beclometasone dipropionate 250.000microgram/1.000dose Pressurised inhalation Inhalation 200000

126641000033118 3103211000001103 Becloforte 400microgram disks with Diskhaler (GlaxoSmithKline UK Ltd) Becloforte 400microgram disks with Diskhaler Beclometasone dipropionate 400.000microgram Inhalation powder Inhalation 6000

126741000033110 3088611000001100 Becodisks 100microgram with Diskhaler (GlaxoSmithKline UK Ltd) Becodisks 100microgram with Diskhaler Beclometasone dipropionate 100.000microgram Inhalation powder Inhalation 40000

126841000033117 3096011000001109 Becodisks 200microgram with Diskhaler (GlaxoSmithKline UK Ltd) Becodisks 200microgram with Diskhaler Beclometasone dipropionate 200.000microgram Inhalation powder Inhalation 70000

126941000033113 3102211000001109 Becodisks 400microgram with Diskhaler (GlaxoSmithKline UK Ltd) Becodisks 400microgram with Diskhaler Beclometasone dipropionate 400.000microgram Inhalation powder Inhalation 40000

129041000033117 3178811000001101 Beclazone 100 inhaler (Teva UK Ltd) Beclazone 100 inhaler Beclometasone dipropionate 100.000microgram/1.000dose Pressurised inhalation Inhalation 400000

129141000033118 3176411000001104 Beclazone 50 inhaler (Teva UK Ltd) Beclazone 50 inhaler Beclometasone dipropionate 50.000microgram/1.000dose Pressurised inhalation Inhalation 90000

129241000033113 3180611000001109 Beclazone 250 inhaler (Teva UK Ltd) Beclazone 250 inhaler Beclometasone dipropionate 250.000microgram/1.000dose Pressurised inhalation Inhalation 200000

129841000033112 3178611000001100 Becotide 100 inhaler (GlaxoSmithKline UK Ltd) Becotide 100 inhaler Beclometasone dipropionate 100.000microgram/1.000dose Pressurised inhalation Inhalation 2000000

129941000033116 3176211000001103 Becotide 50 inhaler (GlaxoSmithKline UK Ltd) Becotide 50 inhaler Beclometasone dipropionate 50.000microgram/1.000dose Pressurised inhalation Inhalation 700000

131441000033112 3179311000001104 Becotide 200 inhaler (GlaxoSmithKline UK Ltd) Becotide 200 inhaler Beclometasone dipropionate 200.000microgram/1.000dose Pressurised inhalation Inhalation 500000

133941000033115 2940411000001108 Beconase Aqueous 50micrograms/dose nasal spray (GlaxoSmithKline UK Ltd) Beconase Aqueous 50micrograms/dose nasal spray Beclometasone dipropionate 50.000microgram/1.000dose Spray Nasal 6000000

136641000033115 3105311000001107 Becloforte 400microgram disks (GlaxoSmithKline UK Ltd) Becloforte 400microgram disks Beclometasone dipropionate 400.000microgram Inhalation powder Inhalation 7000

136941000033110 3086011000001108 Becodisks 100microgram (GlaxoSmithKline UK Ltd) Becodisks 100microgram Beclometasone dipropionate 100.000microgram Inhalation powder Inhalation 90000

137041000033111 3099811000001109 Becodisks 200microgram (GlaxoSmithKline UK Ltd) Becodisks 200microgram Beclometasone dipropionate 200.000microgram Inhalation powder Inhalation 200000

137241000033115 3104911000001109 Becodisks 400microgram (GlaxoSmithKline UK Ltd) Becodisks 400microgram Beclometasone dipropionate 400.000microgram Inhalation powder Inhalation 300000

137641000033117 3190311000001103 Becotide 100microgram Rotacaps (GlaxoSmithKline UK Ltd) Becotide 100microgram Rotacaps Beclometasone dipropionate 100.000microgram Inhalation powder Inhalation 100000

137741000033114 3192111000001101 Becotide 200microgram Rotacaps (GlaxoSmithKline UK Ltd) Becotide 200microgram Rotacaps Beclometasone dipropionate 200.000microgram Inhalation powder Inhalation 500000

138041000033110 3194011000001108 Becotide 400microgram Rotacaps (GlaxoSmithKline UK Ltd) Becotide 400microgram Rotacaps Beclometasone dipropionate 400.000microgram Inhalation powder Inhalation 200000

576141000033117 3177911000001105 Filair 100 inhaler (Meda Pharmaceuticals Ltd) Filair 100 inhaler Beclometasone dipropionate 100.000microgram/1.000dose Pressurised inhalation Inhalation 40000

576241000033112 3175211000001109 Filair 50 inhaler (Meda Pharmaceuticals Ltd) Filair 50 inhaler Beclometasone dipropionate 50.000microgram/1.000dose Pressurised inhalation Inhalation 6000

576341000033119 4753511000001101 Filair Forte 250micrograms/dose inhaler (Meda Pharmaceuticals Ltd) Filair Forte 250micrograms/dose inhaler Beclometasone dipropionate 250.000microgram/1.000dose Pressurised inhalation Inhalation 20000

948441000033117 2940611000001106 Nasobec Aqueous 50micrograms/dose nasal spray (Teva UK Ltd) Nasobec Aqueous 50micrograms/dose nasal spray Beclometasone dipropionate 50.000microgram/1.000dose Spray Nasal 100000

1505841000033112 3292811000001106 Ventide inhaler (GlaxoSmithKline UK Ltd) Ventide inhaler Beclometasone dipropionate/ Salbutamol 50.000microgram/1.000dose + 100.000microgram/1.000dose Pressurised inhalation Inhalation 200000

1510841000033119 3200511000001109 Ventide Paediatric Rotacaps (GlaxoSmithKline UK Ltd) Ventide Paediatric Rotacaps Beclometasone dipropionate/ Salbutamol sulfate 100.000microgram + 200.000microgram Inhalation powder Inhalation 1000

1511041000033117 3202211000001107 Ventide Rotacaps (GlaxoSmithKline UK Ltd) Ventide Rotacaps Beclometasone dipropionate/ Salbutamol sulfate 200.000microgram + 400.000microgram Inhalation powder Inhalation 10000

1571041000033110 3112511000001109 Asmabec 100 Clickhaler (Focus Pharmaceuticals Ltd) Asmabec 100 Clickhaler Beclometasone dipropionate 100.000microgram/1.000dose Inhalation powder Inhalation 100000

1571141000033114 3174111000001102 Asmabec 250 Clickhaler (Focus Pharmaceuticals Ltd) Asmabec 250 Clickhaler Beclometasone dipropionate 250.000microgram/1.000dose Inhalation powder Inhalation 50000

1571241000033119 3111911000001108 Asmabec 50 Clickhaler (Focus Pharmaceuticals Ltd) Asmabec 50 Clickhaler Beclometasone dipropionate 50.000microgram/1.000dose Inhalation powder Inhalation 10000

1577941000033118 3179511000001105 Beclazone 200 inhaler (Teva UK Ltd) Beclazone 200 inhaler Beclometasone dipropionate 200.000microgram/1.000dose Pressurised inhalation Inhalation 50000

1671041000033116 3176811000001102 Qvar 50 inhaler (Teva UK Ltd) Qvar 50 inhaler Beclometasone dipropionate 50.000microgram/1.000dose Pressurised inhalation Inhalation 2000000

1671141000033117 3175611000001106 Qvar 100 inhaler (Teva UK Ltd) Qvar 100 inhaler Beclometasone dipropionate 100.000microgram/1.000dose Pressurised inhalation Inhalation 4000000

1671241000033112 3177411000001102 Qvar 50 Autohaler (Teva UK Ltd) Qvar 50 Autohaler Beclometasone dipropionate 50.000microgram/1.000dose Pressurised inhalation Inhalation 400000

1671341000033119 3177711000001108 Qvar 100 Autohaler (Teva UK Ltd) Qvar 100 Autohaler Beclometasone dipropionate 100.000microgram/1.000dose Pressurised inhalation Inhalation 700000

2916141000033116 4636511000001101 Rino Clenil 50micrograms/dose nasal spray (Chiesi Ltd) Rino Clenil 50micrograms/dose nasal spray Beclometasone dipropionate 50.000microgram/1.000dose Spray Nasal 800

3199241000033112 8159511000001107 Qvar 50micrograms/dose Easi-Breathe inhaler (Teva UK Ltd) Qvar 50micrograms/dose Easi-Breathe inhaler Beclometasone dipropionate 50.000microgram/1.000dose Pressurised inhalation Inhalation 400000

3199341000033119 8159711000001102 Qvar 100micrograms/dose Easi-Breathe inhaler (Teva UK Ltd) Qvar 100micrograms/dose Easi-Breathe inhaler Beclometasone dipropionate 100.000microgram/1.000dose Pressurised inhalation Inhalation 600000

3943041000033111 10617711000001103 Clenil Modulite 50micrograms/dose inhaler (Chiesi Ltd) Clenil Modulite 50micrograms/dose inhaler Beclometasone dipropionate 50.000microgram/1.000dose Pressurised inhalation Inhalation 4000000

3943341000033113 10618211000001109 Clenil Modulite 100micrograms/dose inhaler (Chiesi Ltd) Clenil Modulite 100micrograms/dose inhaler Beclometasone dipropionate 100.000microgram/1.000dose Pressurised inhalation Inhalation 10000000

3943441000033119 10619311000001107 Clenil Modulite 200micrograms/dose inhaler (Chiesi Ltd) Clenil Modulite 200micrograms/dose inhaler Beclometasone dipropionate 200.000microgram/1.000dose Pressurised inhalation Inhalation 3000000

3943641000033117 10619611000001102 Clenil Modulite 250micrograms/dose inhaler (Chiesi Ltd) Clenil Modulite 250micrograms/dose inhaler Beclometasone dipropionate 250.000microgram/1.000dose Pressurised inhalation Inhalation 800000

4158841000033110 2942511000001108 Beconase Hayfever 50micrograms/dose nasal spray (Omega Pharma Ltd) Beconase Hayfever 50micrograms/dose nasal spray Beclometasone dipropionate 50.000microgram/1.000dose Spray Nasal 40000

4418241000033117 12906411000001100 Fostair 100micrograms/dose / 6micrograms/dose inhaler (Chiesi Ltd) Fostair 100micrograms/dose / 6micrograms/dose inhaler Beclometasone dipropionate/ Formoterol fumarate dihydrate 100.000microgram/1.000dose + 6.000microgram/1.000dose Pressurised inhalation Inhalation 9000000

9537441000033115 26112111000001106 Fostair NEXThaler 100micrograms/dose / 6micrograms/dose dry powder inhaler (Chiesi Ltd) Fostair NEXThaler 100micrograms/dose / 6micrograms/dose dry powder inhaler Beclometasone dipropionate/ Formoterol fumarate dihydrate 100.000microgram/1.000dose + 6.000microgram/1.000dose Inhalation powder Inhalation 1000000

10735141000033116 31063111000001106 Fostair NEXThaler 200micrograms/dose / 6micrograms/dose dry powder inhaler (Chiesi Ltd) Fostair NEXThaler 200micrograms/dose / 6micrograms/dose dry powder inhaler Beclometasone dipropionate/ Formoterol fumarate dihydrate 200.000microgram/1.000dose + 6.000microgram/1.000dose Inhalation powder Inhalation 400000

10740141000033110 31063411000001101 Fostair 200micrograms/dose / 6micrograms/dose inhaler (Chiesi Ltd) Fostair 200micrograms/dose / 6micrograms/dose inhaler Beclometasone dipropionate/ Formoterol fumarate dihydrate 200.000microgram + 6.000microgram Pressurised inhalation Inhalation 2000000

12364641000033110 34681611000001100 Trimbow 87micrograms/dose / 5micrograms/dose / 9micrograms/dose inhaler (Chiesi Ltd) Trimbow 87micrograms/dose / 5micrograms/dose / 9micrograms/dose inhaler Beclometasone dipropionate/ Formoterol fumarate dihydrate/ Glycopyrronium bromide 87.000microgram/1.000dose + 5.000microgram/1.000dose + 9.000microgram/1.000dose Pressurised inhalation Inhalation 2000000

12530441000033112 35430311000001103 Kelhale 100micrograms/dose inhaler (Cipla EU Ltd) Kelhale 100micrograms/dose inhaler Beclometasone dipropionate 100.000microgram/1.000dose Pressurised inhalation Inhalation 80000

12530541000033113 35430111000001100 Kelhale 50micrograms/dose inhaler (Cipla EU Ltd) Kelhale 50micrograms/dose inhaler Beclometasone dipropionate 50.000microgram/1.000dose Pressurised inhalation Inhalation 40000

12901341000033112 36603211000001106 Soprobec 50micrograms/dose inhaler (Glenmark Pharmaceuticals Europe Ltd) Soprobec 50micrograms/dose inhaler Beclometasone dipropionate 50.000microgram/1.000dose Pressurised inhalation Inhalation 50000

12901441000033118 36603611000001108 Soprobec 100micrograms/dose inhaler (Glenmark Pharmaceuticals Europe Ltd) Soprobec 100micrograms/dose inhaler Beclometasone dipropionate 100.000microgram/1.000dose Pressurised inhalation Inhalation 200000

12901541000033117 36603411000001105 Soprobec 200micrograms/dose inhaler (Glenmark Pharmaceuticals Europe Ltd) Soprobec 200micrograms/dose inhaler Beclometasone dipropionate 200.000microgram/1.000dose Pressurised inhalation Inhalation 40000

12901641000033116 36603811000001107 Soprobec 250micrograms/dose inhaler (Glenmark Pharmaceuticals Europe Ltd) Soprobec 250micrograms/dose inhaler Beclometasone dipropionate 250.000microgram/1.000dose Pressurised inhalation Inhalation 4000

13860241000033114 39817511000001103 Luforbec 100micrograms/dose / 6micrograms/dose inhaler (Lupin Healthcare (UK) Ltd) Luforbec 100micrograms/dose / 6micrograms/dose inhaler Beclometasone dipropionate/ Formoterol fumarate dihydrate 100.000microgram/1.000dose + 6.000microgram/1.000dose Pressurised inhalation Inhalation 100000

13912841000033115 39993311000001105 Trimbow NEXThaler 88micrograms/dose / 5micrograms/dose / 9micrograms/dose dry powder inhaler (Chiesi Ltd) Trimbow NEXThaler 88micrograms/dose / 5micrograms/dose / 9micrograms/dose dry powder inhaler Beclometasone dipropionate/ Formoterol fumarate dihydrate/ Glycopyrronium bromide 88.000microgram/1.000dose + 5.000microgram/1.000dose + 9.000microgram/1.000dose Inhalation powder Inhalation 80000

14045741000033118 40752211000001109 Trimbow 172micrograms/dose / 5micrograms/dose / 9micrograms/dose inhaler (Chiesi Ltd) Trimbow 172micrograms/dose / 5micrograms/dose / 9micrograms/dose inhaler Beclometasone dipropionate/ Formoterol fumarate dihydrate/ Glycopyrronium bromide 172.000microgram/1.000dose + 5.000microgram/1.000dose + 9.000microgram/1.000dose Pressurised inhalation Inhalation 20000

14060041000033117 40852311000001103 Luforbec 200micrograms/dose / 6micrograms/dose inhaler (Lupin Healthcare (UK) Ltd) Luforbec 200micrograms/dose / 6micrograms/dose inhaler Beclometasone dipropionate/ Formoterol fumarate dihydrate 200.000microgram + 6.000microgram Pressurised inhalation Inhalation 20000

14174141000033112 41475411000001100 Beclu 100micrograms/dose inhaler (Lupin Healthcare (UK) Ltd) Beclu 100micrograms/dose inhaler Beclometasone dipropionate 100.000microgram/1.000dose Pressurised inhalation Inhalation 20

14212241000033118 41963811000001107 Bibecfo 100micrograms/dose / 6micrograms/dose inhaler (Cipla EU Ltd) Bibecfo 100micrograms/dose / 6micrograms/dose inhaler Beclometasone dipropionate/ Formoterol fumarate dihydrate 100.000microgram/1.000dose + 6.000microgram/1.000dose Pressurised inhalation Inhalation 4

1143041000033117 2924111000001109 Pulmicort 200micrograms/dose inhaler (AstraZeneca UK Ltd) Pulmicort 200micrograms/dose inhaler Budesonide 200.000microgram/1.000dose Pressurised inhalation Inhalation 400000

1143141000033118 3240911000001108 Pulmicort LS 50micrograms/dose inhaler (AstraZeneca UK Ltd) Pulmicort LS 50micrograms/dose inhaler Budesonide 50.000microgram/1.000dose Pressurised inhalation Inhalation 100000

1144441000033111 3635411000001106 Pulmicort 0.5mg Respules (AstraZeneca UK Ltd) Pulmicort 0.5mg Respules Budesonide 250.000microgram/1.000ml Nebuliser liquid Inhalation 80000

1144541000033112 3636511000001103 Pulmicort 1mg Respules (AstraZeneca UK Ltd) Pulmicort 1mg Respules Budesonide 500.000microgram/1.000ml Nebuliser liquid Inhalation 100000

1145141000033119 3112411000001105 Pulmicort 200 Turbohaler (AstraZeneca UK Ltd) Pulmicort 200 Turbohaler Budesonide 200.000microgram/1.000dose Inhalation powder Inhalation 1000000

1145241000033114 3228711000001106 Pulmicort 400 Turbohaler (AstraZeneca UK Ltd) Pulmicort 400 Turbohaler Budesonide 400.000microgram/1.000dose Inhalation powder Inhalation 600000

1145341000033116 3113111000001106 Pulmicort 100 Turbohaler (AstraZeneca UK Ltd) Pulmicort 100 Turbohaler Budesonide 100.000microgram/1.000dose Inhalation powder Inhalation 400000

1168741000033118 544311000001102 Rhinocort Aqua 100micrograms/dose nasal spray (AstraZeneca UK Ltd) Rhinocort Aqua 100micrograms/dose nasal spray Budesonide 100.000microgram/1.000dose Spray Nasal 300000

2587541000033118 3294211000001101 Symbicort 100/6 Turbohaler (AstraZeneca UK Ltd) Symbicort 100/6 Turbohaler Budesonide/ Formoterol fumarate dihydrate 100.000microgram/1.000dose + 6.000microgram/1.000dose Inhalation powder Inhalation 1000000

2587641000033117 3294611000001104 Symbicort 200/6 Turbohaler (AstraZeneca UK Ltd) Symbicort 200/6 Turbohaler Budesonide/ Formoterol fumarate dihydrate 200.000microgram/1.000dose + 6.000microgram/1.000dose Inhalation powder Inhalation 6000000

2798341000033112 4860811000001104 Pulmicort 200micrograms/dose inhaler with Nebuchamber (AstraZeneca UK Ltd) Pulmicort 200micrograms/dose inhaler with Nebuchamber Budesonide 200.000microgram/1.000dose Pressurised inhalation Inhalation 400

2905641000033115 4373811000001100 Symbicort 400/12 Turbohaler (AstraZeneca UK Ltd) Symbicort 400/12 Turbohaler Budesonide/ Formoterol fumarate dihydrate 400.000microgram/1.000dose + 12.000microgram/1.000dose Inhalation powder Inhalation 2000000

3075941000033119 7401811000001109 Rhinocort Aqua 64 nasal spray (McNeil Products Ltd) Rhinocort Aqua 64 nasal spray Budesonide 64.000microgram/1.000dose Spray Nasal 200000

4815241000033115 14951111000001102 Pulmicort 100micrograms/dose inhaler CFC free (AstraZeneca UK Ltd) Pulmicort 100micrograms/dose inhaler CFC free Budesonide 100.000microgram/1.000dose Pressurised inhalation Inhalation 4000

4815341000033113 15358411000001102 Pulmicort 200micrograms/dose inhaler CFC free (AstraZeneca UK Ltd) Pulmicort 200micrograms/dose inhaler CFC free Budesonide 200.000microgram/1.000dose Pressurised inhalation Inhalation 5000

5393341000033118 8031811000001102 Budelin Novolizer 200micrograms/dose inhalation powder (Viatris UK Healthcare Ltd) Budelin Novolizer 200micrograms/dose inhalation powder Budesonide 200.000microgram/1.000dose Inhalation powder Inhalation 4000

5393441000033112 9111811000001100 Budelin Novolizer 200micrograms/dose inhalation powder refill (Viatris UK Healthcare Ltd) Budelin Novolizer 200micrograms/dose inhalation powder refill Budesonide 200.000microgram/1.000dose Inhalation powder Inhalation 3000

9342741000033111 25254111000001105 DuoResp Spiromax 160micrograms/dose / 4.5micrograms/dose dry powder inhaler (Teva UK Ltd) DuoResp Spiromax 160micrograms/dose / 4.5micrograms/dose dry powder inhaler Budesonide/ Formoterol fumarate dihydrate 200.000microgram/1.000dose + 6.000microgram/1.000dose Inhalation powder Inhalation 1000000

9342841000033118 25254711000001106 DuoResp Spiromax 320micrograms/dose / 9micrograms/dose dry powder inhaler (Teva UK Ltd) DuoResp Spiromax 320micrograms/dose / 9micrograms/dose dry powder inhaler Budesonide/ Formoterol fumarate dihydrate 400.000microgram/1.000dose + 12.000microgram/1.000dose Inhalation powder Inhalation 800000

11707341000033113 32926011000001100 Symbicort 200micrograms/dose / 6micrograms/dose pressurised inhaler (AstraZeneca UK Ltd) Symbicort 200micrograms/dose / 6micrograms/dose pressurised inhaler Budesonide/ Formoterol fumarate dihydrate 200.000microgram/1.000dose + 6.000microgram/1.000dose Pressurised inhalation Inhalation 70000

12403741000033110 34812111000001106 Fobumix Easyhaler 320micrograms/dose / 9micrograms/dose dry powder inhaler (Orion Pharma (UK) Ltd) Fobumix Easyhaler 320micrograms/dose / 9micrograms/dose dry powder inhaler Budesonide/ Formoterol fumarate dihydrate 400.000microgram/1.000dose + 12.000microgram/1.000dose Inhalation powder Inhalation 20000

12430741000033111 34950311000001108 Fobumix Easyhaler 160micrograms/dose / 4.5micrograms/dose dry powder inhaler (Orion Pharma (UK) Ltd) Fobumix Easyhaler 160micrograms/dose / 4.5micrograms/dose dry powder inhaler Budesonide/ Formoterol fumarate dihydrate 200.000microgram/1.000dose + 6.000microgram/1.000dose Inhalation powder Inhalation 100000

12434441000033111 34950611000001103 Fobumix Easyhaler 80micrograms/dose / 4.5micrograms/dose dry powder inhaler (Orion Pharma (UK) Ltd) Fobumix Easyhaler 80micrograms/dose / 4.5micrograms/dose dry powder inhaler Budesonide/ Formoterol fumarate dihydrate 100.000microgram/1.000dose + 6.000microgram/1.000dose Inhalation powder Inhalation 20000

12476741000033110 35089811000001104 Benacort 64micrograms nasal spray (McNeil Products Ltd) Benacort 64micrograms nasal spray Budesonide 64.000microgram/1.000dose Spray Nasal 400

13743941000033118 39105811000001102 Symbicort 100micrograms/dose / 3micrograms/dose pressurised inhaler (AstraZeneca UK Ltd) Symbicort 100micrograms/dose / 3micrograms/dose pressurised inhaler Budesonide/ Formoterol fumarate dihydrate 100.000microgram/1.000dose + 3.000microgram/1.000dose Pressurised inhalation Inhalation 3000

13774841000033112 39327311000001104 Trixeo Aerosphere 5micrograms/dose / 7.2micrograms/dose / 160micrograms/dose pressurised inhaler (AstraZeneca UK Ltd) Trixeo Aerosphere 5micrograms/dose / 7.2micrograms/dose / 160micrograms/dose pressurised inhaler Budesonide/ Formoterol fumarate dihydrate/ Glycopyrronium bromide 160.000microgram/1.000dose + 5.000microgram/1.000dose + 7.200microgram/1.000dose Pressurised inhalation Inhalation 30000

13915941000033118 40106011000001102 WockAIR 160micrograms/dose / 4.5micrograms/dose dry powder inhaler (Wockhardt UK Ltd) WockAIR 160micrograms/dose / 4.5micrograms/dose dry powder inhaler Budesonide/ Formoterol fumarate dihydrate 200.000microgram/1.000dose + 6.000microgram/1.000dose Inhalation powder Inhalation 100

13916041000033111 40106211000001107 WockAIR 320micrograms/dose / 9micrograms/dose dry powder inhaler (Wockhardt UK Ltd) WockAIR 320micrograms/dose / 9micrograms/dose dry powder inhaler Budesonide/ Formoterol fumarate dihydrate 400.000microgram/1.000dose + 12.000microgram/1.000dose Inhalation powder Inhalation 20

1401741000033111 656911000001100 Syntaris 0.025% nasal spray (Teva UK Ltd) Syntaris 0.025% nasal spray Flunisolide 25.000microgram/1.000dose Spray Nasal 200000

931141000033119 39109911000001109 Mometasone 50micrograms/dose nasal spray Mometasone 50micrograms/dose nasal spray Mometasone furoate 50.000microgram/1.000dose Spray Nasal 12020100 10000000

953341000033116 123711000001109 Nasonex 50micrograms/dose nasal spray (Organon Pharma (UK) Ltd) Nasonex 50micrograms/dose nasal spray Mometasone furoate 50.000microgram/1.000dose Spray Nasal 2000000

5811241000033111 38895411000001109 Mometasone 200micrograms/dose dry powder inhaler Mometasone 200micrograms/dose dry powder inhaler Mometasone furoate 200.000microgram/1.000dose Inhalation powder Inhalation 3020000 7000

5811341000033118 38895611000001107 Mometasone 400micrograms/dose dry powder inhaler Mometasone 400micrograms/dose dry powder inhaler Mometasone furoate 400.000microgram/1.000dose Inhalation powder Inhalation 3020000 10000

5811441000033112 4045711000001107 Asmanex 200micrograms/dose Twisthaler (Organon Pharma (UK) Ltd) Asmanex 200micrograms/dose Twisthaler Mometasone furoate 200.000microgram/1.000dose Inhalation powder Inhalation 4000

5811541000033113 4043811000001103 Asmanex 400micrograms/dose Twisthaler (Organon Pharma (UK) Ltd) Asmanex 400micrograms/dose Twisthaler Mometasone furoate 400.000microgram/1.000dose Inhalation powder Inhalation 4000

13741241000033114 39116311000001103 Indacaterol 125micrograms/dose / Mometasone 127.5micrograms/dose inhalation powder capsules with device Indacaterol 125micrograms/dose / Mometasone 127.5micrograms/dose inhalation powder capsules with device Indacaterol acetate/ Mometasone furoate 125.000microgram/1.000dose + 127.500microgram/1.000dose Inhalation powder Inhalation 3020000 6

13741441000033110 39116511000001109 Indacaterol 125micrograms/dose / Mometasone 62.5micrograms/dose inhalation powder capsules with device Indacaterol 125micrograms/dose / Mometasone 62.5micrograms/dose inhalation powder capsules with device Indacaterol acetate/ Mometasone furoate 125.000microgram/1.000dose + 62.500microgram/1.000dose Inhalation powder Inhalation 3020000 40

13741541000033111 39115411000001101 Atectura Breezhaler 125micrograms/127.5micrograms inhalation powder capsules with device (Novartis Pharmaceuticals UK Ltd) Atectura Breezhaler 125micrograms/127.5micrograms inhalation powder capsules with device Indacaterol acetate/ Mometasone furoate 125.000microgram/1.000dose + 127.500microgram/1.000dose Inhalation powder Inhalation 80

13741641000033112 39115911000001109 Atectura Breezhaler 125micrograms/260micrograms inhalation powder capsules with device (Novartis Pharmaceuticals UK Ltd) Atectura Breezhaler 125micrograms/260micrograms inhalation powder capsules with device Indacaterol acetate/ Mometasone furoate 125.000microgram/1.000dose + 260.000microgram/1.000dose Inhalation powder Inhalation 60

13741741000033115 39114511000001103 Atectura Breezhaler 125micrograms/62.5micrograms inhalation powder capsules with device (Novartis Pharmaceuticals UK Ltd) Atectura Breezhaler 125micrograms/62.5micrograms inhalation powder capsules with device Indacaterol acetate/ Mometasone furoate 125.000microgram/1.000dose + 62.500microgram/1.000dose Inhalation powder Inhalation 200

13744141000033117 39134711000001102 Enerzair Breezhaler 114micrograms/dose / 46micrograms/dose / 136micrograms/dose inhalation powder capsules with device (Novartis Pharmaceuticals UK Ltd) Enerzair Breezhaler 114micrograms/dose / 46micrograms/dose / 136micrograms/dose inhalation powder capsules with device Glycopyrronium bromide/ Indacaterol acetate/ Mometasone furoate 46.000microgram/1.000dose + 114.000microgram/1.000dose + 136.000microgram/1.000dose Inhalation powder Inhalation 3000

13909941000033118 40015611000001105 Mometasone 25micrograms/dose / Olopatadine 600micrograms/dose nasal spray Mometasone 25micrograms/dose / Olopatadine 600micrograms/dose nasal spray Mometasone furoate/ Olopatadine hydrochloride 25.000microgram/1.000dose + 600.000microgram/1.000dose Spray Nasal 12020100 1000

13910041000033114 39970511000001103 Ryaltris 25micrograms/dose / 600micrograms/dose nasal spray (Glenmark Pharmaceuticals Europe Ltd) Ryaltris 25micrograms/dose / 600micrograms/dose nasal spray Mometasone furoate/ Olopatadine hydrochloride 25.000microgram/1.000dose + 600.000microgram/1.000dose Spray Nasal 2000

948541000033116 269011000001101 Nasacort 55micrograms/dose nasal spray (Sanofi Consumer Healthcare) Nasacort 55micrograms/dose nasal spray Triamcinolone acetonide 55.000microgram/1.000dose Spray Nasal 400000

1453241000033112 38750811000001108 Triamcinolone 55micrograms/dose nasal spray Triamcinolone 55micrograms/dose nasal spray Triamcinolone acetonide 55.000microgram/1.000dose Spray Nasal 12020100 800000

4954841000033119 15249411000001104 Nasacort Allergy 55micrograms/dose nasal spray (Sanofi Consumer Healthcare) Nasacort Allergy 55micrograms/dose nasal spray Triamcinolone acetonide 55.000microgram/1.000dose Spray Nasal 2000

585641000033110 Flixotide Inhaler 125 micrograms/puff 91206

585841000033111 Flixotide Inhaler 50 micrograms/puff 44873

586241000033117 Flixotide Inhaler 250 micrograms/puff 211399

1510941000033110 852211000001102 Ventide Rotahaler (GlaxoSmithKline UK Ltd) Ventide Rotahaler 2621

3081741000033112 42292311000001104 Beclometasone 100micrograms/dose inhaler Beclometasone 100micrograms/dose inhaler Beclometasone dipropionate 100.000microgram/1.000dose Pressurised inhalation Inhalation 3020000 9962332

5707041000033111 17313711000001108 Indacaterol 150microgram inhalation powder capsules with device Indacaterol 150microgram inhalation powder capsules with device Indacaterol maleate 150.000microgram Inhalation powder Inhalation 3010101 62094

5707141000033110 17313811000001104 Indacaterol 300microgram inhalation powder capsules with device Indacaterol 300microgram inhalation powder capsules with device Indacaterol maleate 300.000microgram Inhalation powder Inhalation 3010101 13081

9851941000033116 28049611000001104 Indacaterol 85micrograms/dose / Glycopyrronium bromide 54micrograms/dose inhalation powder capsules with device Indacaterol 85micrograms/dose / Glycopyrronium bromide 54micrograms/dose inhalation powder capsules with device Glycopyrronium bromide/ Indacaterol maleate 54.000microgram/1.000dose + 85.000microgram/1.000dose Inhalation powder Inhalation 3010400 29885

14174241000033116 41476211000001104 Beclu 200micrograms/dose inhaler (Lupin Healthcare (UK) Ltd) Beclu 200micrograms/dose inhaler Beclometasone dipropionate 200.000microgram/1.000dose Pressurised inhalation Inhalation 381

14212341000033112 41966111000001104 Bibecfo 200micrograms/dose / 6micrograms/dose inhaler (Cipla EU Ltd) Bibecfo 200micrograms/dose / 6micrograms/dose inhaler Beclometasone dipropionate/ Formoterol fumarate dihydrate 200.000microgram + 6.000microgram Pressurised inhalation Inhalation 18

14265341000033116 42490511000001104 Fluticasone 400microgram/unit dose nasal drops (Imported (Spain)) Fluticasone 400microgram/unit dose nasal drops Fluticasone propionate 400.000microgram Nasal drops 2

ProdCodeId DMDCode TermfromEMIS ProductName drugsubstancename substancestrength formulation routeofadministration bnfcode DrugIssues

8056941000033114 38893611000001108 Aclidinium bromide 375micrograms/dose dry powder inhaler Aclidinium bromide 375micrograms/dose dry powder inhaler Aclidinium bromide 375.000microgram/1.000dose Inhalation powder Inhalation 3010200 300000

8057341000033112 20985511000001101 Eklira 322micrograms/dose Genuair (Zentiva Pharma UK Ltd) Eklira 322micrograms/dose Genuair Aclidinium bromide 375.000microgram/1.000dose Inhalation powder Inhalation 600000

9995641000033112 28365011000001100 Aclidinium bromide 396micrograms/dose / Formoterol 11.8micrograms/dose dry powder inhaler Aclidinium bromide 396micrograms/dose / Formoterol 11.8micrograms/dose dry powder inhaler Aclidinium bromide/ Formoterol fumarate dihydrate 396.000microgram/1.000dose + 11.800microgram/1.000dose Inhalation powder Inhalation 3010400 40000

9995741000033115 28357211000001106 Duaklir 340micrograms/dose / 12micrograms/dose Genuair (Zentiva Pharma UK Ltd) Duaklir 340micrograms/dose / 12micrograms/dose Genuair Aclidinium bromide/ Formoterol fumarate dihydrate 396.000microgram/1.000dose + 11.800microgram/1.000dose Inhalation powder Inhalation 400000

13768041000033118 39329111000001107 Bevespi Aerosphere 7.2micrograms/dose / 5micrograms/dose pressurised inhaler (AstraZeneca UK Ltd) Bevespi Aerosphere 7.2micrograms/dose / 5micrograms/dose pressurised inhaler Formoterol fumarate dihydrate/ Glycopyrronium bromide 5.000microgram/1.000dose + 7.200microgram/1.000dose Pressurised inhalation Inhalation 10000

13767941000033116 39343611000001104 Glycopyrronium 7.2micrograms/dose / Formoterol 5micrograms/dose inhaler CFC free Glycopyrronium 7.2micrograms/dose / Formoterol 5micrograms/dose inhaler CFC free Formoterol fumarate dihydrate/ Glycopyrronium bromide 5.000microgram/1.000dose + 7.200microgram/1.000dose Pressurised inhalation Inhalation 3010400 1000

9852041000033114 28007211000001102 Ultibro Breezhaler 85microgram/43microgram inhalation powder capsules with device (Novartis Pharmaceuticals UK Ltd) Ultibro Breezhaler 85microgram/43microgram inhalation powder capsules with device Glycopyrronium bromide/ Indacaterol maleate 54.000microgram/1.000dose + 85.000microgram/1.000dose Inhalation powder Inhalation 400000

9851941000033115 28049611000001104 Indacaterol 85micrograms/dose / Glycopyrronium bromide 54micrograms/dose inhalation powder capsules with device Indacaterol 85micrograms/dose / Glycopyrronium bromide 54micrograms/dose inhalation powder capsules with device Glycopyrronium bromide/ Indacaterol maleate 54.000microgram/1.000dose + 85.000microgram/1.000dose Inhalation powder Inhalation 3010400 30000

8141741000033112 21496211000001102 Glycopyrronium bromide 55microgram inhalation powder capsules with device Glycopyrronium bromide 55microgram inhalation powder capsules with device Glycopyrronium bromide 55.000microgram Inhalation powder Inhalation 3010200 200000

8141841000033119 21495411000001107 Seebri Breezhaler 44microgram inhalation powder capsules with device (Novartis Pharmaceuticals UK Ltd) Seebri Breezhaler 44microgram inhalation powder capsules with device Glycopyrronium bromide 55.000microgram Inhalation powder Inhalation 800000

88241000033118 24011000001103 Atrovent 20micrograms/dose inhaler (Boehringer Ingelheim Ltd) Atrovent 20micrograms/dose inhaler Ipratropium bromide 20.000microgram/1.000dose Pressurised inhalation Inhalation 600000

88341000033111 3235911000001100 Atrovent 40microgram Aerocaps (Boehringer Ingelheim Ltd) Atrovent 40microgram Aerocaps Ipratropium bromide 40.000microgram Inhalation powder Inhalation 20000

88441000033117 3238911000001106 Atrovent 40microgram Aerocaps with Aerohaler (Boehringer Ingelheim Ltd) Atrovent 40microgram Aerocaps with Aerohaler Ipratropium bromide 40.000microgram Inhalation powder Inhalation 10000

88541000033116 351411000001106 Atrovent 20micrograms/dose Autohaler (Boehringer Ingelheim Ltd) Atrovent 20micrograms/dose Autohaler Ipratropium bromide 20.000microgram/1.000dose Pressurised inhalation Inhalation 100000

89441000033110 347611000001103 Atrovent Forte 40micrograms/dose inhaler (Boehringer Ingelheim Ltd) Atrovent Forte 40micrograms/dose inhaler Ipratropium bromide 40.000microgram/1.000dose Pressurised inhalation Inhalation 100000

90041000033110 3222411000001102 Atrovent 500micrograms/2ml nebuliser liquid UDVs (Boehringer Ingelheim Ltd) Atrovent 500micrograms/2ml nebuliser liquid UDVs Ipratropium bromide 250.000microgram/1.000ml Nebuliser liquid Inhalation 200000

330241000033113 3348611000001107 Combivent inhaler (Boehringer Ingelheim Ltd) Combivent inhaler Ipratropium bromide/ Salbutamol sulfate 20.000microgram/1.000dose + 100.000microgram/1.000dose Pressurised inhalation Inhalation 2000000

485841000033117 2964011000001104 Duovent Autohaler (Boehringer Ingelheim Ltd) Duovent Autohaler Fenoterol hydrobromide/ Ipratropium bromide 100.000microgram/1.000dose + 40.000microgram/1.000dose Pressurised inhalation Inhalation 50000

488041000033119 2923111000001107 Duovent inhaler (Boehringer Ingelheim Ltd) Duovent inhaler Fenoterol hydrobromide/ Ipratropium bromide 100.000microgram/1.000dose + 40.000microgram/1.000dose Pressurised inhalation Inhalation 600000

491541000033117 4192411000001109 Duovent UDVs nebuliser liquid 4ml (Boehringer Ingelheim Ltd) Duovent UDVs nebuliser liquid 4ml Fenoterol hydrobromide/ Ipratropium bromide 312.500microgram/1.000ml + 125.000microgram/1.000ml Nebuliser liquid Inhalation 6000

772541000033111 36049411000001105 Ipratropium bromide 40micrograms/dose inhaler Ipratropium bromide 40micrograms/dose inhaler Ipratropium bromide 40.000microgram/1.000dose Pressurised inhalation Inhalation 3010200 300000

772641000033112 36049311000001103 Ipratropium bromide 20micrograms/dose inhaler Ipratropium bromide 20micrograms/dose inhaler Ipratropium bromide 20.000microgram/1.000dose Pressurised inhalation Inhalation 3010200 1000000

772841000033113 36049211000001106 Ipratropium bromide 20micrograms/dose breath actuated inhaler Ipratropium bromide 20micrograms/dose breath actuated inhaler Ipratropium bromide 20.000microgram/1.000dose Pressurised inhalation Inhalation 3010200 200000

772941000033117 39717411000001100 Ipratropium bromide 40microgram inhalation powder capsules Ipratropium bromide 40microgram inhalation powder capsules Ipratropium bromide 40.000microgram Inhalation powder Inhalation 3010200 20000

773041000033110 39717311000001107 Ipratropium bromide 40microgram inhalation powder capsules with device Ipratropium bromide 40microgram inhalation powder capsules with device Ipratropium bromide 40.000microgram Inhalation powder Inhalation 3010200 20000

773341000033112 38896611000001102 Ipratropium bromide 21micrograms/dose nasal spray Ipratropium bromide 21micrograms/dose nasal spray Ipratropium bromide 21.000microgram/1.000dose Spray Nasal 12020200 400000

773741000033113 3227211000001103 Ipratropium 250micrograms/1ml nebuliser liquid Steri-Neb unit dose vials (Teva UK Ltd) Ipratropium 250micrograms/1ml nebuliser liquid Steri-Neb unit dose vials Ipratropium bromide 250.000microgram/1.000ml Nebuliser liquid Inhalation 60000

773841000033115 3223111000001101 Ipratropium 500micrograms/2ml nebuliser liquid Steri-Neb unit dose vials (Teva UK Ltd) Ipratropium 500micrograms/2ml nebuliser liquid Steri-Neb unit dose vials Ipratropium bromide 250.000microgram/1.000ml Nebuliser liquid Inhalation 60000

773941000033111 39717511000001101 Ipratropium bromide 500micrograms/2ml nebuliser liquid unit dose vials Ipratropium bromide 500micrograms/2ml nebuliser liquid unit dose vials Ipratropium bromide 250.000microgram/1.000ml Nebuliser liquid Inhalation 3010200 800000

1672141000033113 3227011000001108 Respontin 250micrograms/1ml Nebules (GlaxoSmithKline UK Ltd) Respontin 250micrograms/1ml Nebules Ipratropium bromide 250.000microgram/1.000ml Nebuliser liquid Inhalation 4000

1672241000033118 3222911000001105 Respontin 500micrograms/2ml Nebules (GlaxoSmithKline UK Ltd) Respontin 500micrograms/2ml Nebules Ipratropium bromide 250.000microgram/1.000ml Nebuliser liquid Inhalation 4000

1702541000033116 3225811000001107 Tropiovent 250micrograms/1ml nebuliser liquid unit dose Steripoule vials (Ashbourne Pharmaceuticals Ltd) Tropiovent 250micrograms/1ml nebuliser liquid unit dose Steripoule vials Ipratropium bromide 250.000microgram/1.000ml Nebuliser liquid Inhalation 50

1702641000033115 3221911000001104 Tropiovent 500micrograms/2ml nebuliser liquid unit dose Steripoule vials (Ashbourne Pharmaceuticals Ltd) Tropiovent 500micrograms/2ml nebuliser liquid unit dose Steripoule vials Ipratropium bromide 250.000microgram/1.000ml Nebuliser liquid Inhalation 500

1818641000033117 3406011000001104 Combivent nebuliser liquid 2.5ml UDVs (Boehringer Ingelheim Ltd) Combivent nebuliser liquid 2.5ml UDVs Ipratropium bromide/ Salbutamol sulfate 200.000microgram/1.000ml + 1.000mg/1.000ml Nebuliser liquid Inhalation 600000

1860741000033110 36066611000001106 Fenoterol 100micrograms/dose / Ipratropium 40micrograms/dose inhaler Fenoterol 100micrograms/dose / Ipratropium 40micrograms/dose inhaler Fenoterol hydrobromide/ Ipratropium bromide 100.000microgram/1.000dose + 40.000microgram/1.000dose Pressurised inhalation Inhalation 3010400 80000

3075441000033112 38894511000001107 Ipratropium bromide 20micrograms/dose inhaler CFC free Ipratropium bromide 20micrograms/dose inhaler CFC free Ipratropium bromide 20.000microgram/1.000dose Pressurised inhalation Inhalation 3010200 4000000

3075541000033113 7389911000001105 Atrovent 20micrograms/dose inhaler CFC free (Boehringer Ingelheim Ltd) Atrovent 20micrograms/dose inhaler CFC free Ipratropium bromide 20.000microgram/1.000dose Pressurised inhalation Inhalation 800000

3163441000033113 35936311000001102 Salbutamol 100micrograms/dose / Ipratropium 20micrograms/dose inhaler Salbutamol 100micrograms/dose / Ipratropium 20micrograms/dose inhaler Ipratropium bromide/ Salbutamol sulfate 20.000microgram/1.000dose + 100.000microgram/1.000dose Pressurised inhalation Inhalation 3010400 500000

3163541000033114 39710511000001100 Salbutamol 2.5mg/2.5ml / Ipratropium bromide 500micrograms/2.5ml nebuliser liquid unit dose vials Salbutamol 2.5mg/2.5ml / Ipratropium bromide 500micrograms/2.5ml nebuliser liquid unit dose vials Ipratropium bromide/ Salbutamol sulfate 200.000microgram/1.000ml + 1.000mg/1.000ml Nebuliser liquid Inhalation 3010400 200000

3163641000033110 39711511000001107 Fenoterol 1.25mg/4ml / Ipratropium 500micrograms/4ml nebuliser liquid unit dose vials Fenoterol 1.25mg/4ml / Ipratropium 500micrograms/4ml nebuliser liquid unit dose vials Fenoterol hydrobromide/ Ipratropium bromide 312.500microgram/1.000ml + 125.000microgram/1.000ml Nebuliser liquid Inhalation 3010400 1000

4023941000033117 10927511000001104 Ipramol nebuliser solution 2.5ml Steri-Neb unit dose vials (Teva UK Ltd) Ipramol nebuliser solution 2.5ml Steri-Neb unit dose vials Ipratropium bromide/ Salbutamol sulfate 200.000microgram/1.000ml + 1.000mg/1.000ml Nebuliser liquid Inhalation 5000

4519441000033116 9039611000001103 Ipratropium 250micrograms/1ml nebuliser liquid unit dose Steripoule vials (Galen Ltd) Ipratropium 250micrograms/1ml nebuliser liquid unit dose Steripoule vials Ipratropium bromide 250.000microgram/1.000ml Nebuliser liquid Inhalation 1000

4958441000033114 39717211000001104 Ipratropium bromide 250micrograms/1ml nebuliser liquid unit dose vials Ipratropium bromide 250micrograms/1ml nebuliser liquid unit dose vials Ipratropium bromide 250.000microgram/1.000ml Nebuliser liquid Inhalation 3010200 100000

4958541000033110 3226411000001101 Atrovent 250micrograms/1ml nebuliser liquid UDVs (Boehringer Ingelheim Ltd) Atrovent 250micrograms/1ml nebuliser liquid UDVs Ipratropium bromide 250.000microgram/1.000ml Nebuliser liquid Inhalation 40000

5072941000033118 9039811000001104 Ipratropium 500micrograms/2ml nebuliser liquid unit dose Steripoule vials (Galen Ltd) Ipratropium 500micrograms/2ml nebuliser liquid unit dose Steripoule vials Ipratropium bromide 250.000microgram/1.000ml Nebuliser liquid Inhalation 700

5098441000033118 15534911000001100 Salbutamol 2.5mg/2.5ml / Ipratropium bromide 500micrograms/2.5ml nebuliser liquid ampoules Salbutamol 2.5mg/2.5ml / Ipratropium bromide 500micrograms/2.5ml nebuliser liquid ampoules Ipratropium bromide/ Salbutamol sulfate 200.000microgram/1.000ml + 1.000mg/1.000ml Nebuliser liquid Inhalation 3010400 5000

5098641000033116 32898611000001109 Salipraneb 0.5mg/2.5mg nebuliser solution 2.5ml ampoules (Actavis UK Ltd) Salipraneb 0.5mg/2.5mg nebuliser solution 2.5ml ampoules Ipratropium bromide/ Salbutamol sulfate 200.000microgram/1.000ml + 1.000mg/1.000ml Nebuliser liquid Inhalation 20

12430241000033117 34954811000001109 Inhalvent 20micrograms/dose inhaler (Alissa Healthcare Research Ltd) Inhalvent 20micrograms/dose inhaler Ipratropium bromide 20.000microgram/1.000dose Pressurised inhalation Inhalation 100

13283241000033112 37842911000001109 Ipravent 20micrograms/dose inhaler CFC free (Cipla EU Ltd) Ipravent 20micrograms/dose inhaler CFC free Ipratropium bromide 20.000microgram/1.000dose Pressurised inhalation Inhalation 100

13557241000033117 38617811000001107 Combiprasal 0.5mg/2.5mg nebuliser solution 2.5ml unit dose vials (TriOn Pharma Ltd) Combiprasal 0.5mg/2.5mg nebuliser solution 2.5ml unit dose vials Ipratropium bromide/ Salbutamol sulfate 200.000microgram/1.000ml + 1.000mg/1.000ml Nebuliser liquid Inhalation 6

13771741000033116 37693111000001103 Xylometazoline 70micrograms/dose / Ipratropium bromide 84micrograms/dose nasal spray Xylometazoline 70micrograms/dose / Ipratropium bromide 84micrograms/dose nasal spray Ipratropium bromide/ Xylometazoline hydrochloride 84.000microgram/1.000dose + 70.000microgram/1.000dose Spray Nasal 12020200 70

14030141000033114 3346411000001108 Rinaspray 21micrograms/dose nasal spray (Sanofi Consumer Healthcare) Rinaspray 21micrograms/dose nasal spray Ipratropium bromide 21.000microgram/1.000dose Spray Nasal 3000

1022141000033115 2947411000001107 Oxivent 100micrograms/dose Autohaler (Boehringer Ingelheim Ltd) Oxivent 100micrograms/dose Autohaler Oxitropium bromide 100.000microgram/1.000dose Pressurised inhalation Inhalation 20000

1024041000033114 36022911000001105 Oxitropium bromide 100micrograms/dose inhaler Oxitropium bromide 100micrograms/dose inhaler Oxitropium bromide 100.000microgram/1.000dose Pressurised inhalation Inhalation 3010200 200000

1024141000033113 2944211000001101 Oxivent 100micrograms/dose inhaler (Boehringer Ingelheim Ltd) Oxivent 100micrograms/dose inhaler Oxitropium bromide 100.000microgram/1.000dose Pressurised inhalation Inhalation 200000

2793541000033114 9479011000001103 Tiotropium bromide 18microgram inhalation powder capsules with device Tiotropium bromide 18microgram inhalation powder capsules with device Tiotropium bromide 18.000microgram Inhalation powder Inhalation 3010200 2000000

2793641000033110 9478911000001107 Tiotropium bromide 18microgram inhalation powder capsules Tiotropium bromide 18microgram inhalation powder capsules Tiotropium bromide 18.000microgram Inhalation powder Inhalation 3010200 10000000

2793741000033118 3378211000001106 Spiriva 18microgram inhalation powder capsules with HandiHaler (Boehringer Ingelheim Ltd) Spiriva 18microgram inhalation powder capsules with HandiHaler Tiotropium bromide 18.000microgram Inhalation powder Inhalation 400000

2793841000033111 3380011000001106 Spiriva 18microgram inhalation powder capsules (Boehringer Ingelheim Ltd) Spiriva 18microgram inhalation powder capsules Tiotropium bromide 18.000microgram Inhalation powder Inhalation 1000000

4270641000033112 12197411000001102 Tiotropium bromide 2.5micrograms/dose solution for inhalation cartridge with device CFC free Tiotropium bromide 2.5micrograms/dose solution for inhalation cartridge with device CFC free Tiotropium bromide 2.500microgram/1.000dose Inhalation solution Inhalation 3010200 1000000

4270741000033115 12146911000001103 Spiriva Respimat 2.5micrograms/dose inhalation solution cartridge with device (Boehringer Ingelheim Ltd) Spiriva Respimat 2.5micrograms/dose inhalation solution cartridge with device Tiotropium bromide 2.500microgram/1.000dose Inhalation solution Inhalation 2000000

10589141000033112 29987211000001108 Tiotropium bromide 2.5micrograms/dose / Olodaterol 2.5micrograms/dose solution for inhalation cartridge with device CFC free Tiotropium bromide 2.5micrograms/dose / Olodaterol 2.5micrograms/dose solution for inhalation cartridge with device CFC free Olodaterol hydrochloride/ Tiotropium bromide 2.500microgram/1.000dose + 2.500microgram/1.000dose Inhalation solution Inhalation 3010400 40000

10589241000033117 29971311000001100 Spiolto Respimat 2.5micrograms/dose / 2.5micrograms/dose inhalation solution cartridge with device (Boehringer Ingelheim Ltd) Spiolto Respimat 2.5micrograms/dose / 2.5micrograms/dose inhalation solution cartridge with device Olodaterol hydrochloride/ Tiotropium bromide 2.500microgram/1.000dose + 2.500microgram/1.000dose Inhalation solution Inhalation 400000

11788941000033110 33596311000001107 Tiotropium bromide 10microgram inhalation powder capsules with device Tiotropium bromide 10microgram inhalation powder capsules with device Tiotropium bromide 10.000microgram Inhalation powder Inhalation 3010200 100000

11789041000033118 33594911000001100 Braltus 10microgram inhalation powder capsules with Zonda inhaler (Teva UK Ltd) Braltus 10microgram inhalation powder capsules with Zonda inhaler Tiotropium bromide 10.000microgram Inhalation powder Inhalation 2000000

13178541000033111 37692511000001100 Tiotropium bromide 2.5micrograms/dose / Olodaterol 2.5micrograms/dose inhalation solution cartridge CFC free Tiotropium bromide 2.5micrograms/dose / Olodaterol 2.5micrograms/dose inhalation solution cartridge CFC free Olodaterol hydrochloride/ Tiotropium bromide 2.500microgram/1.000dose + 2.500microgram/1.000dose Inhalation solution Inhalation 3010400 6000

13178641000033112 37678011000001103 Spiolto Respimat 2.5micrograms/dose / 2.5micrograms/dose inhalation solution refill cartridge (Boehringer Ingelheim Ltd) Spiolto Respimat 2.5micrograms/dose / 2.5micrograms/dose inhalation solution refill cartridge Olodaterol hydrochloride/ Tiotropium bromide 2.500microgram/1.000dose + 2.500microgram/1.000dose Inhalation solution Inhalation 80000

13178741000033115 37692711000001105 Tiotropium bromide 2.5micrograms/dose inhalation solution cartridge CFC free Tiotropium bromide 2.5micrograms/dose inhalation solution cartridge CFC free Tiotropium bromide 2.500microgram/1.000dose Inhalation solution Inhalation 3010200 50000

13178841000033113 37677711000001102 Spiriva Respimat 2.5micrograms/dose inhalation solution refill cartridge (Boehringer Ingelheim Ltd) Spiriva Respimat 2.5micrograms/dose inhalation solution refill cartridge Tiotropium bromide 2.500microgram/1.000dose Inhalation solution Inhalation 200000

13179141000033113 37540311000001105 Yanimo Respimat 2.5micrograms/dose / 2.5micrograms/dose inhalation solution cartridge with device (Boehringer Ingelheim Ltd) Yanimo Respimat 2.5micrograms/dose / 2.5micrograms/dose inhalation solution cartridge with device Olodaterol hydrochloride/ Tiotropium bromide 2.500microgram/1.000dose + 2.500microgram/1.000dose Inhalation solution Inhalation 40

13844541000033111 39605711000001106 Tiogiva 18microgram inhalation powder capsules with device (Glenmark Pharmaceuticals Europe Ltd) Tiogiva 18microgram inhalation powder capsules with device Tiotropium bromide 18.000microgram Inhalation powder Inhalation 6000

13844641000033112 39606211000001105 Tiogiva 18microgram inhalation powder capsules (Glenmark Pharmaceuticals Europe Ltd) Tiogiva 18microgram inhalation powder capsules Tiotropium bromide 18.000microgram Inhalation powder Inhalation 3000

13859141000033118 39666311000001103 Acopair 18microgram inhalation powder capsules with NeumoHaler (Viatris UK Healthcare Ltd) Acopair 18microgram inhalation powder capsules with NeumoHaler Tiotropium bromide 18.000microgram Inhalation powder Inhalation 1000

9293241000033114 24645511000001105 Umeclidinium bromide 65micrograms/dose / Vilanterol 22micrograms/dose dry powder inhaler Umeclidinium bromide 65micrograms/dose / Vilanterol 22micrograms/dose dry powder inhaler Umeclidinium bromide/ Vilanterol trifenatate 65.000microgram/1.000dose + 22.000microgram/1.000dose Inhalation powder Inhalation 3010400 80000

9293341000033116 24644611000001108 Anoro Ellipta 55micrograms/dose / 22micrograms/dose dry powder inhaler (GlaxoSmithKline UK Ltd) Anoro Ellipta 55micrograms/dose / 22micrograms/dose dry powder inhaler Umeclidinium bromide/ Vilanterol trifenatate 65.000microgram/1.000dose + 22.000microgram/1.000dose Inhalation powder Inhalation 1000000

9703441000033119 27890611000001109 Umeclidinium bromide 65micrograms/dose dry powder inhaler Umeclidinium bromide 65micrograms/dose dry powder inhaler Umeclidinium bromide 65.000microgram/1.000dose Inhalation powder Inhalation 3010200 100000

9703641000033117 27567911000001101 Incruse Ellipta 55micrograms/dose dry powder inhaler (GlaxoSmithKline UK Ltd) Incruse Ellipta 55micrograms/dose dry powder inhaler Umeclidinium bromide 65.000microgram/1.000dose Inhalation powder Inhalation 1000000

12431141000033117 34952211000001104 Trelegy Ellipta 92micrograms/dose / 55micrograms/dose / 22micrograms/dose dry powder inhaler (GlaxoSmithKline UK Ltd) Trelegy Ellipta 92micrograms/dose / 55micrograms/dose / 22micrograms/dose dry powder inhaler Fluticasone furoate/ Umeclidinium bromide/ Vilanterol trifenatate 92.000microgram/1.000dose + 55.000microgram/1.000dose + 22.000microgram/1.000dose Inhalation powder Inhalation 1000000

567541000033119 4558411000001107 Fenoterol 200micrograms/dose inhaler Fenoterol 200micrograms/dose inhaler Fenoterol hydrobromide 200.000microgram/1.000dose Pressurised inhalation Inhalation 3010101 14579

1221441000033112 35936411000001108 Salbutamol 100micrograms/dose breath actuated inhaler Salbutamol 100micrograms/dose breath actuated inhaler Salbutamol sulfate 100.000microgram/1.000dose Pressurised inhalation Inhalation 3010101 1418661

1222341000033110 39113611000001104 Salbutamol 100micrograms/dose inhaler CFC free Salbutamol 100micrograms/dose inhaler CFC free Salbutamol sulfate 100.000microgram/1.000dose Pressurised inhalation Inhalation 3010101 80442755

1228841000033115 35936711000001104 Salbutamol 200microgram inhalation powder blisters with device Salbutamol 200microgram inhalation powder blisters with device Salbutamol sulfate 200.000microgram Inhalation powder Inhalation 3010101 72441

1228941000033111 35936911000001100 Salbutamol 400microgram inhalation powder blisters with device Salbutamol 400microgram inhalation powder blisters with device Salbutamol sulfate 400.000microgram Inhalation powder Inhalation 3010101 47834

1241441000033110 35936511000001108 Salbutamol 100micrograms/dose inhaler Salbutamol 100micrograms/dose inhaler Salbutamol 100.000microgram/1.000dose Pressurised inhalation Inhalation 3010101 9001627

1245741000033117 39710011000001104 Salbutamol 5mg/2.5ml nebuliser liquid unit dose vials Salbutamol 5mg/2.5ml nebuliser liquid unit dose vials Salbutamol sulfate 2.000mg/1.000ml Nebuliser liquid Inhalation 3010101 1657628

1246041000033112 39709611000001112 Salbutamol 2.5mg/2.5ml nebuliser liquid unit dose vials Salbutamol 2.5mg/2.5ml nebuliser liquid unit dose vials Salbutamol sulfate 1.000mg/1.000ml Nebuliser liquid Inhalation 3010101 2428466

1250141000033112 35936611000001108 Salbutamol 200microgram inhalation powder blisters Salbutamol 200microgram inhalation powder blisters Salbutamol sulfate 200.000microgram Inhalation powder Inhalation 3010101 165247

1250241000033117 35936811000001104 Salbutamol 400microgram inhalation powder blisters Salbutamol 400microgram inhalation powder blisters Salbutamol sulfate 400.000microgram Inhalation powder Inhalation 3010101 347814

1250341000033110 39709511000001104 Salbutamol 5mg/ml nebuliser liquid Salbutamol 5mg/ml nebuliser liquid Salbutamol sulfate 5.000mg/1.000ml Nebuliser liquid Inhalation 3010101 6033

1546041000033117 42197511000001104 Xylometazoline 0.1% nasal spray Xylometazoline 0.1% nasal spray Xylometazoline hydrochloride 1.000mg/1.000ml Spray Nasal 12020200 514134

1546141000033118 35368811000001108 Xylometazoline 0.05% nasal drops Xylometazoline 0.05% nasal drops Xylometazoline hydrochloride 500.000microgram/1.000ml Nasal drops Nasal 12020200 367392

1546241000033113 42197411000001104 Xylometazoline 0.1% nasal drops Xylometazoline 0.1% nasal drops Xylometazoline hydrochloride 1.000mg/1.000ml Nasal drops Nasal 12020200 227048

1751341000033111 39112711000001104 Salbutamol 100micrograms/dose breath actuated inhaler CFC free Salbutamol 100micrograms/dose breath actuated inhaler CFC free Salbutamol sulfate 100.000microgram/1.000dose Pressurised inhalation Inhalation 3010101 7001074

1905641000033119 39709711000001104 Salbutamol 200microgram inhalation powder capsules Salbutamol 200microgram inhalation powder capsules Salbutamol sulfate 200.000microgram Inhalation powder Inhalation 3010101 74980

1905741000033111 39709811000001104 Salbutamol 400microgram inhalation powder capsules Salbutamol 400microgram inhalation powder capsules Salbutamol sulfate 400.000microgram Inhalation powder Inhalation 3010101 203114

1907541000033112 39113311000001104 Salbutamol 200micrograms/dose dry powder inhaler Salbutamol 200micrograms/dose dry powder inhaler Salbutamol 200.000microgram/1.000dose Inhalation powder Inhalation 3010101 1609123

2174141000033117 35937211000001108 Salbutamol 95micrograms/dose dry powder inhaler Salbutamol 95micrograms/dose dry powder inhaler Salbutamol sulfate 95.000microgram/1.000dose Inhalation powder Inhalation 3010101 208987

2622741000033114 3384111000001103 Pulvinal Salbutamol 200micrograms/dose dry powder inhaler (Chiesi Ltd) Pulvinal Salbutamol 200micrograms/dose dry powder inhaler Salbutamol 200.000microgram/1.000dose Inhalation powder Inhalation 82031

2726241000033113 3214611000001103 Salbutamol 200 Cyclocaps (Teva UK Ltd) Salbutamol 200 Cyclocaps Salbutamol sulfate 200.000microgram Inhalation powder Inhalation 3718

2726341000033115 3217611000001109 Salbutamol 400 Cyclocaps (Teva UK Ltd) Salbutamol 400 Cyclocaps Salbutamol sulfate 400.000microgram Inhalation powder Inhalation 7922

3198541000033119 9205211000001104 Easyhaler Salbutamol sulfate 100micrograms/dose dry powder inhaler (Orion Pharma (UK) Ltd) Easyhaler Salbutamol sulfate 100micrograms/dose dry powder inhaler Salbutamol 100.000microgram/1.000dose Inhalation powder Inhalation 1140865

3198641000033118 9204911000001108 Easyhaler Salbutamol sulfate 200micrograms/dose dry powder inhaler (Orion Pharma (UK) Ltd) Easyhaler Salbutamol sulfate 200micrograms/dose dry powder inhaler Salbutamol 200.000microgram/1.000dose Inhalation powder Inhalation 228057

3343941000033118 9207411000001106 Salbutamol 100micrograms/dose dry powder inhaler Salbutamol 100micrograms/dose dry powder inhaler Salbutamol 100.000microgram/1.000dose Inhalation powder Inhalation 3010101 173025

4498841000033119 13566211000001104 Salbutamol 100micrograms/dose dry powder inhalation cartridge with device Salbutamol 100micrograms/dose dry powder inhalation cartridge with device Salbutamol sulfate 100.000microgram/1.000dose Inhalation powder Inhalation 3010101 4942

4498941000033110 13566111000001108 Salbutamol 100micrograms/dose dry powder inhalation cartridge Salbutamol 100micrograms/dose dry powder inhalation cartridge Salbutamol sulfate 100.000microgram/1.000dose Inhalation powder Inhalation 3010101 8059

5707041000033111 17313711000001108 Indacaterol 150microgram inhalation powder capsules with device Indacaterol 150microgram inhalation powder capsules with device Indacaterol maleate 150.000microgram Inhalation powder Inhalation 3010101 62094

5707141000033110 17313811000001104 Indacaterol 300microgram inhalation powder capsules with device Indacaterol 300microgram inhalation powder capsules with device Indacaterol maleate 300.000microgram Inhalation powder Inhalation 3010101 13081

6116941000033117 9040011000001104 Salbutamol 2.5mg/2.5ml nebuliser liquid unit dose Steripoule vials (Galen Ltd) Salbutamol 2.5mg/2.5ml nebuliser liquid unit dose Steripoule vials Salbutamol sulfate 1.000mg/1.000ml Nebuliser liquid Inhalation 1779

6117041000033116 9040211000001108 Salbutamol 5mg/2.5ml nebuliser liquid unit dose Steripoule vials (Galen Ltd) Salbutamol 5mg/2.5ml nebuliser liquid unit dose Steripoule vials Salbutamol sulfate 2.000mg/1.000ml Nebuliser liquid Inhalation 1585

13741241000033114 39116311000001104 Indacaterol 125micrograms/dose / Mometasone 127.5micrograms/dose inhalation powder capsules with device Indacaterol 125micrograms/dose / Mometasone 127.5micrograms/dose inhalation powder capsules with device Indacaterol acetate/ Mometasone furoate 125.000microgram/1.000dose + 127.500microgram/1.000dose Inhalation powder Inhalation 3020000 8

13741441000033110 39116511000001112 Indacaterol 125micrograms/dose / Mometasone 62.5micrograms/dose inhalation powder capsules with device Indacaterol 125micrograms/dose / Mometasone 62.5micrograms/dose inhalation powder capsules with device Indacaterol acetate/ Mometasone furoate 125.000microgram/1.000dose + 62.500microgram/1.000dose Inhalation powder Inhalation 3020000 44

ProdCodeId DMDCode TermfromEMIS ProductName drugsubstancename substancestrength formulation routeofadministration bnfcode DrugIssues

1296441000033114 719811000001104 Singulair 10mg tablets (Organon Pharma (UK) Ltd) Singulair 10mg tablets Montelukast sodium 10.000mg Tablet Oral 300000

1325141000033113 892311000001104 Singulair Paediatric 5mg chewable tablets (Organon Pharma (UK) Ltd) Singulair Paediatric 5mg chewable tablets Montelukast sodium 5.000mg Chewable tablet Oral 70000

2272541000033115 863011000001109 Singulair Paediatric 4mg chewable tablets (Organon Pharma (UK) Ltd) Singulair Paediatric 4mg chewable tablets Montelukast sodium 4.000mg Chewable tablet Oral 40000

3037941000033113 6741711000001104 Singulair Paediatric 4mg granules sachets (Organon Pharma (UK) Ltd) Singulair Paediatric 4mg granules sachets Montelukast sodium 4.000mg Granules Oral 20000

1567141000033111 408011000001106 Accolate 20mg tablets (AstraZeneca UK Ltd) Accolate 20mg tablets Zafirlukast 20.000mg Tablet Oral 30000

961241000033118 36030411000001101 Nedocromil 2mg/dose inhaler with spacer Nedocromil 2mg/dose inhaler with spacer Nedocromil sodium 2.000mg/1.000dose Pressurised inhalation Inhalation 3030100 1000

1436841000033115 4107011000001100 Tilade 2mg/dose inhaler (Sanofi) Tilade 2mg/dose inhaler Nedocromil sodium 2.000mg/1.000dose Pressurised inhalation Inhalation 50000

1443341000033116 4163411000001103 Tilade 2mg/dose Syncroner with spacer (Sanofi) Tilade 2mg/dose Syncroner with spacer Nedocromil sodium 2.000mg/1.000dose Pressurised inhalation Inhalation 5000

3835141000033118 9720211000001107 Nedocromil 2mg/dose inhaler CFC free Nedocromil 2mg/dose inhaler CFC free Nedocromil sodium 2.000mg/1.000dose Pressurised inhalation Inhalation 3030100 20000

3835241000033113 9714111000001103 Tilade 2mg/dose inhaler CFC free (Sanofi) Tilade 2mg/dose inhaler CFC free Nedocromil sodium 2.000mg/1.000dose Pressurised inhalation Inhalation 6000

948941000033110 3842011000001101 Nalcrom 100mg capsules (Sanofi) Nalcrom 100mg capsules Sodium cromoglicate 100.000mg Capsule Oral 20000

1520841000033112 3841611000001102 Vividrin 2% nasal spray (Bausch & Lomb UK Ltd) Vividrin 2% nasal spray Sodium cromoglicate 20.000mg/1.000ml Spray Nasal 2000

3107041000033110 42196411000001101 Sodium cromoglicate 2% nasal spray Sodium cromoglicate 2% nasal spray Sodium cromoglicate 20.000mg/1.000ml Spray Nasal 12020100 40000

3107141000033114 42196511000001102 Sodium cromoglicate 4% nasal spray Sodium cromoglicate 4% nasal spray Sodium cromoglicate 40.000mg/1.000ml Spray Nasal 12020100 200000

13790041000033119 39418311000001103 Sodium cromoglicate 100mg/5ml oral solution 5ml unit dose ampoules sugar free Sodium cromoglicate 100mg/5ml oral solution 5ml unit dose ampoules sugar free Sodium cromoglicate 20.000mg/1.000ml Oral solution Oral 1050400 200

1204741000033114 920711000001103 Rynacrom 4% nasal spray (Sanofi) Rynacrom 4% nasal spray Sodium cromoglicate 40.000mg/1.000ml Spray Nasal 100000

3105741000033118 35926711000001101 Sodium cromoglicate 5mg/dose inhaler Sodium cromoglicate 5mg/dose inhaler Sodium cromoglicate 5.000mg/1.000dose Pressurised inhalation Inhalation 3030100 200000

3105841000033111 4161311000001109 Sodium cromoglicate 5mg/dose inhaler with spacer Sodium cromoglicate 5mg/dose inhaler with spacer Sodium cromoglicate 5.000mg/1.000dose Pressurised inhalation Inhalation 3030100 6000

3106241000033117 3631611000001106 Sodium cromoglicate 20mg/2ml nebuliser liquid unit dose vials Sodium cromoglicate 20mg/2ml nebuliser liquid unit dose vials Sodium cromoglicate 10.000mg/1.000ml Nebuliser liquid Inhalation 3030100 4000

3106341000033110 39712711000001109 Sodium cromoglicate 20mg inhalation powder capsules Sodium cromoglicate 20mg inhalation powder capsules Sodium cromoglicate 20.000mg Inhalation powder Inhalation 3030100 60000

3107241000033119 317421001 Sodium cromoglicate 100mg capsules 1050400 50000

4571341000033117 13801911000001104 Sodium cromoglicate 5mg/dose inhaler CFC free Sodium cromoglicate 5mg/dose inhaler CFC free Sodium cromoglicate 5.000mg/1.000dose Pressurised inhalation Inhalation 3030100 20000
