# Supplementary material for: Burden of allergic rhinitis in the United Kingdom
Source: Front Allergy. 2025 Nov 4;6:1676574. doi: 10.3389/falgy.2025.1676574 (PMC12631609; doi:10.3389/falgy.2025.1676574)
Supplement: Supplementary file 9 [file Table9.docx]

MedCodeId Observations OriginalReadCode CleansedReadCode Term SnomedCTConceptId SnomedCTDescriptionId EmisCodeCategoryId

94731013 20 H35y7 H35y700 Wood asthma 56968009 497732010 32

98546013 900 H3311 H331100 Life threatening acute exacerbation of intrinsic asthma 1086711000000100 2722261000000119 32

104872017 10000 H3300 H330000 Extrinsic asthma without status asthmaticus 63088003 104872017 32

151338014 2000 H3301 H330100 Life threatening acute exacerbation of allergic asthma 1086701000000102 2722231000000112 32

216186011 2000000 663m 663m.00 Emergency asthma patient visit since last encounter 708373002 3034176014 1

264540018 1000000 663N 663N.00 Asthma disturbing sleep 170631002 264540018 1

264541019 300000 663N0 663N000 Asthma causing night waking 170632009 264541019 1

264542014 300000 663N1 663N100 Asthma disturbs sleep weekly 170633004 264542014 1

264543016 300000 663N2 663N200 Asthma disturbs sleep frequently 170634005 264543016 1

264546012 2000000 663P 663P.00 Asthma limiting activities 170637003 264546012 1

264550017 7000000 663U 663U.00 Asthma management 406162001 2157868010 1

264556011 300000 663W 663W.00 Asthma prophylactic medication used 170647000 264556011 1

264565016 100000 663d 663d.00 Emergency asthma admission since last encounter 708358003 3033971010 1

264566015 300000 663e 663e.00 Asthma restricts exercise 170655007 264566015 1

264567012 1000000 663e0 663e000 Asthma sometimes restricts exercise 170656008 264567012 1

264568019 80000 663e1 663e100 Asthma severely restricts exercise 170657004 264568019 1

282488010 100000 8793 8793.00 Asthma control step 0 182726007 282488010 32

282489019 500000 8794 8794.00 Asthma control step 1 182727003 282489019 32

282490011 1000000 8795 8795.00 Asthma control step 2 182728008 282490011 32

282491010 700000 8796 8796.00 Asthma control step 3 182729000 282491010 32

282493013 10000 8798 8798.00 Asthma control step 5 182731009 282493013 32

282492015 80000 8797 8797.00 Asthma control step 4 182730005 282492015 32

283550015 100000 8H2P 8H2P.00 Emergency hospital admission for asthma 183478001 3082895019 40

285727014 100000 9OJ1 9OJ1.00 Attends asthma monitoring 185728001 285727014 37

285728016 80000 9OJ2 9OJ2.00 Asthma monitoring refused 763221007 3638224019 37

285729012 100000 9OJ3 9OJ3.00 Asthma monitor offer default 185730004 285729012 37

285891014 50000 9Q21 9Q21.00 Patient in asthma study 185940009 285891014 37

301450011 9000 H3120 H312000 Chronic asthmatic bronchitis 195949008 301450011 32

301480018 100000 H33-1 H33..11 Bronchial asthma 195967001 301480018 32

301485011 30000000 H33 H33..00 Asthma 195967001 301485011 32

301499010 10000 H330z H330z00 Extrinsic asthma 424643009 2648327010 32

301511014 100000 H33z H33z.00 Asthma unspecified 195967001 301485011 32

331445016 60 TJF7z TJF7z00 Adverse reaction to antiasthmatic 219036002 3300421019 5

331437014 30 TJF7 TJF7.00 Adverse reaction to antiasthmatics 219036002 331437014 5

324412011 6 SLF7z SLF7z00 Poisoning by antiasthmatic 2935001 5971013 32

301509017 10000 H332 H332.00 Mixed asthma 195977004 301509017 32

338238011 10000 H334 H334.00 Brittle asthma 225057002 338238011 32

350147016 90000 H330-2 H330.12 Childhood asthma 233678006 350147016 32

350148014 30000 H331-1 H331.11 Late onset asthma 233679003 350148014 32

350149018 10000 H33z2 H33z200 Late-onset asthma 233679003 350148014 32

350151019 4000 H3301-1 H330111 Acute exacerbation of allergic asthma 708093000 3083642013 32

350152014 6000 H33zz-2 H33zz12 Allergic asthma NEC 389145006 1483199016 32

350154010 20000 H330-4 H330.14 Pollen asthma 233683003 350154010 15

350153016 10000 H3300-1 H330011 Hay fever with asthma 233683003 350153016 15

396118011 10000 H33z0 H33z000 Life threatening acute exacerbation of asthma 734904007 3511368015 32

396119015 200000 H33z1 H33z100 Asthma attack 708038006 3032734012 32

396120014 800000 H33zz H33zz00 Asthma NOS 195967001 301485011 32

405054019 30000 9OJA-1 9OJA.11 Asthma monitored 270442000 405054019 37

405055018 5000000 9OJA 9OJA.00 Asthma monitoring check done 270442000 405055018 37

409865018 10000 H33z1-1 H33z111 Asthma attack NOS 708038006 3032734012 32

411877015 2000000 663-1 663..11 Asthma monitoring 275908000 411877015 1

419211018 2000000 H333 H333.00 Acute exacerbation of asthma 708038006 3032747019 32

456163018 70000 663j 663j.00 Asthma - currently active 312453004 456163018 1

1208954011 2000000 663t 663t.00 Asthma causes daytime symptoms 1 to 2 times per month 370202007 1208954011 1

1208955012 2000000 663u 663u.00 Asthma causes daytime symptoms 1 to 2 times per week 370203002 1208955012 1

1208956013 2000000 663v 663v.00 Asthma causes daytime symptoms most days 370204008 1208956013 1

1208957016 300000 663r 663r.00 Asthma causes night symptoms 1 to 2 times per month 370205009 1208957016 1

1208958014 40000 663x 663x.00 Asthma limits walking on the flat 370206005 1208958014 1

1208959018 200000 663w 663w.00 Asthma limits walking up hills or stairs 370207001 1208959018 1

1208969012 200000 663V1 663V100 Mild asthma 370218001 1208969012 1

1208970013 70000 663V2 663V200 Moderate asthma 370219009 1208970013 1

1208971012 60000 663V0 663V000 Occasional asthma 370220003 1208971012 1

1208972017 20000 663V3 663V300 Severe asthma 370221004 1208972017 1

1208976019 200000 663p 663p.00 Asthma treatment compliance unsatisfactory 370225008 1208976019 1

1208977011 900000 663n 663n.00 Asthma treatment compliance satisfactory 370226009 1208977011 1

1212342016 2000000 663q 663q.00 Asthma daytime symptoms 373899003 1212342016 1

1226348019 4 SLF7 SLF7.00 Antiasthmatic poisoning 2935001 1226348019 32

1483199016 60000 H330-1 H330.11 Allergic asthma 389145006 1483199016 32

1484905010 40000 66Y5 66Y5.00 Change in asthma management plan 390872009 1484905010 1

1484910014 200000 66Y9 66Y9.00 Step up change in asthma management plan 390877003 1484910014 1

1484911013 100000 66YA 66YA.00 Step down change in asthma management plan 390878008 1484911013 1

1484953014 400000 66YC 66YC.00 Absent from work or school due to asthma 390921001 1484953014 1

1484970018 60000 66YE 66YE.00 Asthma monitoring due 390940007 1484970018 1

1488422012 2000000 66YK 66YK.00 Asthma follow-up 394701000 1488422012 1

1488436019 6000000 8B3j 8B3j.00 Asthma medication review 394720003 1488436019 32

1488668018 500000 1J70 1J70.00 Suspected asthma 394967008 1488668018 27

1488722011 200000 66YP 66YP.00 Asthma night-time symptoms 395022009 1488722011 1

1780194017 500000 178 178..00 Asthma trigger 400987003 1780194017 27

1780378019 2000000 66YQ 66YQ.00 Asthma monitoring by nurse 401182001 1780378019 1

1780379010 1000000 66YR 66YR.00 Asthma monitoring by doctor 401183006 1780379010 1

1780388018 80000 1O2 1O2..00 Asthma confirmed 401193004 1780388018 27

2474332015 200000 8CR0 8CR0.00 Asthma clinical management plan 736056000 3516216012 32

2533402016 20000 8HTT 8HTT.00 Referral to asthma clinic 415265005 2533402016 40

3505219018 100 ^ESCT1164318 Asthma monitoring using asthma symptom diary 734346005 3505219018 31

3511374015 10000 ^ESCT1164869 Moderate acute exacerbation of asthma 734905008 3511374015 32

3514315010 40 ^ESCT1170560 Asthma in pregnancy 72301000119103 3514315010 32

3514925011 8 ^ESCT1165691 Acute severe exacerbation of asthma co-occurrent and due to allergic asthma 735587000 3514925011 31

3637387011 2000 ^ESCT1169266 Exacerbation of allergic asthma 762521001 3637387011 32

21611000000113 300000 9OJ 9OJ..00 Asthma monitoring administration 713701000000108 1564941000000112 37

26101000000112 20000 9OJ-1 9OJ..11 Asthma clinic administration 713711000000105 1564951000000110 37

145961000006117 50000 H33z0-1 H33z011 Acute severe exacerbation of asthma 708090002 3033029011 32

149741000006116 2 H35y6 H35y600 Sequoiosis (red-cedar asthma) 23315001 39131015 15

305611000000115 200000 1786 1786.00 Asthma trigger: animals 201051000000101 636211000000110 27

351281000006112 10 U60F6-A U60F61A [X] Adverse reaction to antiasthmatic NOS 219036002 3300421019 5

351291000006110 6 U60F6-1 U60F611 [X] Adverse reaction to antiasthmatics 219036002 331437014 5

471931000006114 300 TJF73 TJF7300 Adverse reaction to theophylline (asthma) 292617004 432760019 5

496311000006117 5000000 9OJ4 9OJ4.00 Asthma monitoring call first letter 185731000 285730019 37

496321000006113 2000000 9OJ5 9OJ5.00 Asthma monitoring call second letter 185732007 285731015 37

496331000006111 1000000 9OJ6 9OJ6.00 Asthma monitoring call third letter 185734008 285734011 37

496351000006116 400000 9OJ8 9OJ8.00 Asthma monitoring call telephone invite 185736005 285736013 37

496361000006119 300000 9OJ7 9OJ7.00 Asthma monitoring call verbal invite 185735009 285735012 37

636291000000118 400000 1789 1789.00 Asthma trigger - respiratory infection 201031000000108 305571000000112 27

636311000000117 200000 1787 1787.00 Asthma trigger - seasonal 201041000000104 305591000000111 27

660351000006118 400000 H330 H330.00 Allergic atopic asthma 389145006 1493645010 32

817361000006114 70000 H330-3 H330.13 Hay fever with asthma 233683003 350153016 15

856031000006110 2000 EMISASC1 Asthma stable < 3 months 856031000006106 856031000006110 1

856051000006115 4000 EMISASC3 Asthma control unsatisfactory 856051000006104 856051000006115 1

857481000006115 200000 EMISNO6 No H/O: asthma 857481000006104 857481000006115 32

857551000006112 4000000 EMISNOFH7 No FH: asthma 857551000006108 857551000006112 7

885291000006115 20000 H330-99 H330.99 Extrinsic asthma - atopy 389145006 885291000006115 32

970911000006110 20 EMISNQRE34 Referral to Asthma clinic 970911000006106 970911000006110 40

1012731000006119 90 ALLERGY3.3 Adverse reaction to Drugs For Prophylaxis Of Asthma 1012731000006103 1012731000006119 5

1052641000006113 2 ALLERGY3501NEMIS Adverse reaction to Asthmahaler Mist 1052641000006109 1052641000006113 5

1139131000000117 2000 9NNX 9NNX.00 Under care of asthma specialist nurse 698509001 2974554010 37

1722421000000110 300000 66Yq 66Yq.00 Asthma causes night time symptoms 1 to 2 times per week 771901000000100 1722421000000110 1

1722501000000119 200000 66Yr 66Yr.00 Asthma causes symptoms most nights 771941000000102 1722501000000119 1

1722581000000112 200000 663P0 663P000 Asthma limits activities 1 to 2 times per month 771981000000105 1722581000000112 1

1722651000000119 200000 663P1 663P100 Asthma limits activities 1 to 2 times per week 772011000000107 1722651000000119 1

1722731000000116 100000 663P2 663P200 Asthma limits activities most days 772051000000106 1722731000000116 1

1739131000000111 200000 178A 178A.00 Asthma trigger - airborne dust 340891000000106 1739131000000111 27

1739151000000116 200000 1781 1781.00 Asthma trigger - pollen 340911000000109 1739151000000116 27

1739161000000118 50000 1782 1782.00 Asthma trigger - tobacco smoke 340921000000103 1739161000000118 27

1807891000006111 1 JHCAS1 Asthma causes daytime asthma symptoms less than weekly 1807891000006107 1807891000006111 1

1807901000006110 1 JHCAS2 Asthma causes daytime symptoms more than weekly, less than daily 1807901000006106 1807901000006110 1

1807911000006113 1 JHCAS3 Asthma causes daytime asthma symptoms daily 1807911000006109 1807911000006113 1

1807921000006117 1 JHCAS4 Asthma causes night time symptoms less than 2 times per month 1807921000006101 1807921000006117 1

1807931000006119 1 JHCAS5 Asthma causes night time symp more than 2 times a month,not wkly 1807931000006103 1807931000006119 1

1807941000006112 1 JHCAS6 Asthma causes night time asthma symptoms weekly or more often 1807941000006108 1807941000006112 1

1811901000006116 40000 66Yu 66Yu.00 Number of days absent from school due to asthma in past 6 months 811151000000105 2116171000000118 1

1821501000006116 1 JHCFR1 Frequent night time asthma symptoms 1821501000006100 1821501000006116 1

1821511000006118 1 JHCIN17 Infrequent asthma exacerbations 1821511000006102 1821511000006118 1

1821531000006112 1 JHCFR2 Frequent asthma exacerbations 1821531000006108 1821531000006112 1

1855521000006111 7000 EMISNQAS34 Asthma monitoring in primary care 1855521000006107 1855521000006111 1

1855531000006114 300 EMISNQAS35 Asthma monitoring in secondary care 1855531000006105 1855531000006114 1

1856351000006118 2000 EMISNQFO32 Follow-up asthma assessment 1856351000006102 1856351000006118 1

1859261000006117 5000 EMISNQDA36 Date of asthma diagnosis 1859261000006101 1859261000006117 1

1880001000006114 60000 EMISNQNO108 No change in asthma management plan 1880001000006105 1880001000006114 1

1927091000006116 2000000 9OJB 9OJB.00 Asthma monitoring invitation SMS (short message service) text message 928451000000107 2379671000000115 37

1947591000006113 600000 9OJB0 9OJB000 Asthma monitoring SMS (short message service) text message first invitation 959401000000101 2445551000000116 37

1947601000006117 200000 9OJB1 9OJB100 Asthma monitoring SMS (short message service) text message second invitation 959421000000105 2445611000000116 37

1947611000006119 60000 9OJB2 9OJB200 Asthma monitoring SMS (short message service) text message third invitation 959441000000103 2445651000000117 37

1948051000006112 50000 H3B H3B..00 Asthma-chronic obstructive pulmonary disease overlap syndrome 10692761000119107 3046456013 32

2009981000006110 100 EMISNQDI256 Difficult asthma 2009981000006106 2009981000006110 1

2010041000006114 6000 EMISNQAC877 Acute non-infective exacerbation of asthma 2010041000006105 2010041000006114 32

2240591000000119 10000 H335 H335.00 Chronic asthma with fixed airflow obstruction 866881000000101 2240591000000119 32

2423691000000114 4000 66Yz5 66Yz500 Telehealth asthma monitoring 715191006 3301824014 1

2446671000000118 500 ^ESCT1171175 Asthma action plan 959901000000107 2446671000000118 31

2460311000000112 8000 14Ok0 14Ok000 At risk of severe asthma exacerbation 966011000000109 2460311000000112 32

5054341000006110 10 ^ESCTLA505434 Late-onset asthma 233679003 350149018 32

4781531000006113 200 ^ESCTAS478153 Asthmatic 195967001 301479016 32

5054401000006116 2 ^ESCTFA505440 Factitious asthma 233690008 350163012 31

5649191000006110 40000 ^ESCTEX564919 Exacerbation of asthma 281239006 419210017 32

5904331000006116 400 ^ESCTAS590433 Asthma control steps 302220000 443801018 31

5979441000006111 4000 ^ESCTAS597944 Asthma monitoring call 308500005 451860019 37

5979451000006113 1000 ^ESCTAS597945 Asthma monitoring status 308501009 451861015 1

6512381000006116 100 ^ESCTAT651238 Atopic asthma 389145006 1493644014 32

6550131000006111 1 ^ESCTNO655013 Nocturnal asthma 395022009 2956617010 1

6724151000006111 70 ^ESCTAS672415 Asthma care 406162001 2163236010 1

6782071000006115 500 ^ESCTCO678207 Cough variant asthma 409663006 2471432015 32

7030311000006117 700 ^ESCTIG703031 IgE-mediated allergic asthma 424643009 2644386013 32

7030321000006113 2 ^ESCTIG703032 IgE mediated asthma 424643009 2648326018 32

7030341000006118 1 ^ESCTIG703034 IgE mediated allergic asthma 424643009 2648328017 32

7052221000006118 2000 ^ESCTEX705222 Exacerbation of intermittent asthma 425969006 2674141011 32

7062421000006112 30 ^ESCTSE706242 Severe persistent asthma 426656000 2674140012 32

7067211000006115 3000 ^ESCTMI706721 Mild persistent asthma 426979002 2674138019 32

7076331000006112 4000 ^ESCTIN707633 Intermittent asthma 427603009 2674136015 32

7071891000006112 20 ^ESCTMO707189 Moderate persistent asthma 427295004 2674139010 32

7077481000006119 10000 ^ESCTMI707748 Mild intermittent asthma 427679007 2674137012 32

7306161000006113 10 ^ESCTAS730616 Asthma nurse specialist 445313000 2872677016 31

7307941000006113 3000 ^ESCTSE730794 Seasonal asthma 445427006 2872543019 32

7617601000006114 1 ^ESCTUN761760 Uncomplicated asthma 707444001 3032638017 32

7617611000006112 2000 ^ESCTEX761761 Exacerbation of mild persistent asthma 707445000 3030146012 32

7617621000006116 400 ^ESCTEX761762 Exacerbation of moderate persistent asthma 707446004 3030142014 32

7617631000006118 100 ^ESCTEX761763 Exacerbation of severe persistent asthma 707447008 3030154014 32

7618791000006110 90 ^ESCTUN761879 Uncomplicated mild persistent asthma 707511009 3032641014 32

7618811000006114 1 ^ESCTUN761881 Uncomplicated severe persistent asthma 707513007 3032636018 32

7626521000006110 8 ^ESCTAC762652 Acute severe exacerbation of severe persistent asthma 707979007 3032643012 32

7626531000006113 30 ^ESCTAC762653 Acute severe exacerbation of moderate persistent asthma 707980005 3032646016 32

7626541000006115 200 ^ESCTAC762654 Acute severe exacerbation of mild persistent asthma 707981009 3032647013 32

7628271000006114 3 ^ESCTAC762827 Acute exacerbation of extrinsic asthma 708093000 3032949014 32

7628301000006111 3 ^ESCTAC762830 Acute severe exacerbation of allergic asthma 708095007 3083689010 32

7628321000006118 7 ^ESCTAC762832 Acute severe exacerbation of immunoglobin E-mediated allergic asthma 708095007 3083685016 32

7687651000006111 1 ^ESCTAC768765 Acute asthma control management 711388008 3078166012 31

7688501000006112 600 ^ESCTRE768850 Recent asthma management 711442001 3078609015 31

7961281000006114 20 ^ESCTAC796128 Acute severe asthma 733858005 3474969012 32

7966291000006118 2000 ^ESCTIN796629 Intermittent asthma well controlled 641000119106 3041227011 1

7970131000006117 700 ^ESCTIN797013 Intermittent asthma uncontrolled 1741000119102 3041058017 32

7970171000006119 50 ^ESCTAC797017 Acute exacerbation of chronic obstructive airways disease with asthma 1751000119100 2967380018 32

8031201000006110 100 ^ESCTAC803120 Acute exacerbation of asthma co-occurrent with allergic rhinitis 99031000119107 3040730014 32

8038931000006119 6 ^ESCTSE803893 Severe persistent asthma co-occurrent with allergic rhinitis 124991000119109 3038312015 32

8038941000006112 3 ^ESCTMO803894 Moderate persistent asthma co-occurrent with allergic rhinitis 125001000119103 3043951015 32

8038961000006111 90 ^ESCTMI803896 Mild persistent asthma co-occurrent with allergic rhinitis 125011000119100 3040894010 32

8038971000006116 80 ^ESCTIN803897 Intermittent asthma co-occurrent with allergic rhinitis 125021000119107 3041241019 32

8042571000006111 20 ^ESCTAC804257 Acute exacerbation of moderate persistent asthma 135171000119106 3043548019 32

8042581000006114 40 ^ESCTAC804258 Acute exacerbation of mild persistent asthma 135181000119109 3043530011 32

8060811000006111 70000 ^ESCTAS806081 Asthma trigger - seasonal 201041000000104 636311000000117 27

8109001000006118 70000 ^ESCTAS810900 Asthma trigger - airborne dust 340891000000106 634981000000119 27

8109041000006116 100000 ^ESCTAS810904 Asthma trigger - pollen 340911000000109 635021000000115 27

9314961000006112 10 ^ESCTAC931496 Acute severe exacerbation of asthma co-occurrent with allergic rhinitis 10674711000119105 3043983014 32

9315101000006110 1 ^ESCTAC931510 Acute severe exacerbation of severe persistent allergic asthma 10675471000119109 3043806016 32

9315111000006113 2 ^ESCTAC931511 Acute severe exacerbation of severe persistent asthma co-occurrent with allergic rhinitis 10675551000119104 3043762011 32

9315121000006117 1 ^ESCTSE931512 Severe persistent allergic asthma controlled 10675591000119109 3083393013 1

9315141000006112 1 ^ESCTSE931514 Severe persistent asthma controlled co-occurrent with allergic rhinitis 10675631000119109 3083256016 32

9315181000006118 30 ^ESCTSE931518 Severe uncontrolled persistent asthma 10675751000119107 3424789015 32

9315201000006117 1000 ^ESCTMI931520 Mild persistent allergic asthma 10675871000119106 3043169011 32

9315221000006110 10 ^ESCTAC931522 Acute severe exacerbation of mild persistent allergic asthma 10675911000119109 3043234015 32

9315231000006113 10 ^ESCTAC931523 Acute severe exacerbation of mild persistent allergic asthma co-occurrent with allergic rhinitis 10675991000119100 3043192011 32

9315251000006118 800 ^ESCTMI931525 Mild persistent allergic asthma controlled 10676031000119106 3083332018 1

9315261000006116 10000 ^ESCTMI931526 Mild persistent asthma controlled 10676071000119109 3083485012 1

9315281000006114 100 ^ESCTMI931528 Mild persistent asthma controlled co-occurrent with allergic rhinitis 10676111000119102 3083630015 32

9315291000006112 40 ^ESCTMI931529 Mild persistent allergic asthma uncontrolled 10676151000119101 3083309017 1

9315301000006113 300 ^ESCTMI931530 Mild persistent asthma uncontrolled 10676191000119106 3083629013 1

9315321000006115 2 ^ESCTMI931532 Mild persistent asthma uncontrolled co-occurrent with allergic rhinitis 10676231000119102 3083628017 32

9315341000006110 60 ^ESCTMO931534 Moderate persistent asthma controlled 10676351000119103 3083637017 1

9315371000006119 3 ^ESCTAC931537 Acute severe exacerbation of moderate persistent allergic asthma 10676431000119103 3043819017 32

9315351000006112 5 ^ESCTMO931535 Moderate persistent allergic asthma 10676391000119108 3043556016 32

9315391000006118 7 ^ESCTAC931539 Acute severe exacerbation of moderate persistent asthma co-occurrent with allergic rhinitis 10676511000119109 3043960011 32

9315401000006116 2 ^ESCTMO931540 Moderate persistent allergic asthma controlled 10676551000119105 3083626018 1

9315431000006112 2 ^ESCTMO931543 Moderate persistent allergic asthma uncontrolled 10676631000119100 3083365014 1

9315441000006119 1 ^ESCTMO931544 Moderate persistent asthma uncontrolled co-occurrent with allergic rhinitis 10676671000119102 3083625019 32

9315461000006115 100 ^ESCTMO931546 Moderate persistent asthma uncontrolled 10676711000119103 3083432017 1

9317311000006119 10 ^ESCTCH931731 Chronic obstructive asthma co-occurrent with acute exacerbation of asthma 10692721000119102 3038325011 32

9317331000006113 30 ^ESCTAS931733 Asthma-COPD overlap syndrome (ACOS) 10692761000119107 3046475015 32

9317341000006115 200 ^ESCTAC931734 ACOS - asthma-chronic obstructive pulmonary disease overlap syndrome 10692761000119107 2496071000000117 32

9934311000006111 20 ^ESCTOR993431 Oral steroid-dependent asthma 16584951000119101 3427554014 32

11922851000006115 4000 ^ESCT1192285 Acute asthma 304527002 446841017 32

13619851000006113 30 ^ESCT1361985 Acute severe exacerbation of allergic asthma 782513000 3754324015 32

13619961000006119 600 ^ESCT1361996 Exacerbation of allergic asthma due to infection 782520007 3754350016 32

13659841000006117 10 ^ESCT1365984 Near fatal asthma 786836003 3772564015 32

13775501000006113 90 ^ESCT1377550 Allergic asthma without status asthmaticus 63088003 3754351017 32

13979511000006116 1 ^ESCT1397951 Asthma medication declined 9521000175103 3964118015 31

13997031000006117 300 ^ESCT1399703 Intermittent allergic asthma 10674991000119104 3777826016 32

14504061000006110 30000 ^ESCT1450406 Acute asthma 281239006 4535308012 32

14504071000006115 600 ^ESCT1450407 Asthma attack 281239006 4535229017 32

15060791000006116 1 ^ESCT1506079 Acute exacerbation of moderate persistent allergic asthma 10676271000119104 5089452010 32
